# Tracing the geographic origin of Atlantic cod products using stable isotope analysis

**DOI:** 10.1002/rcm.9861

**Published:** 2024-07-22

**Authors:** Juliet S. E. Wilson, Rona A. R. McGill, Petur Steingrund, Clive N. Trueman

**Affiliations:** ^1^ Ocean and Earth Science University of Southampton Southampton UK; ^2^ Marine Management Organisation, Environment Agency Romsey Office Romsey UK; ^3^ National Environmental Isotope Facility Scottish Universities Environmental Research Centre Glasgow UK; ^4^ Faroe Marine Research Institute Faroe Islands Denmark

## Abstract

**Rationale:**

Increasing demand for fish and seafood means that the traceability of marine products is becoming ever more important for consumers, producers and regulators. Highly complex and globalised supply networks create challenges for verifying a stated catch region. Atlantic cod is one of the most commercially important species in the northeast Atlantic. Several regional fisheries supply cod into the trade network, of which some are at greater risk of overexploitation than others. Tools allowing retrospective testing of spatial origin would significantly assist sustainable harvesting of fish, reducing incentives for illegal fishing and fraud.

**Methods:**

Here, we investigate whether stable isotope ratios of carbon, nitrogen and sulphur can be used to retrospectively identify the catch region of Atlantic cod ( *Gadus morhua* ). We measured the isotopic composition of muscle tissue from 377 cod from 10 catch regions across the northeast Atlantic and then applied three different assignment methods to classify cod by region of most likely origin. The assignment method developed was subsequently tested using independently sourced, known‐origin samples.

**Results:**

Individual cod could be traced back to their true origin with an average assignment accuracy of 70–79% and over 90% accuracy for certain regions. Assignment success rates comparable to those using genetic techniques were achieved when assigning among restricted and pre‐selected regions. However, assignment accuracy to the fishery region estimated from independent samples across the whole geographic range of cod averaged ~25% overall, highlighting the need for careful application of isotope‐based approaches.

**Conclusion:**

Stable isotope techniques can provide effective tools to test for origin in Atlantic cod, but not all catch regions are isotopically distinct. Stable isotopes could be combined with genetic techniques to result in higher assignment accuracy than could be achieved using either method independently. Assignment potential can be estimated from reference datasets, but estimates of realistic assignment accuracy require independently collected data.

## 1 INTRODUCTION

Traceability of marine products throughout retail chains is increasingly important for consumers, producers and regulators.^1^, ^2^, ^3^, ^4^ Food fraud is known to be a serious global issue,^5^, ^6^, ^7^, ^8^, ^9^ whether substituting the species in a product, the undeclared use of additives, fraudulent use of a brand name or concealing the true geographic origin.^8^ Fish and seafood are particularly vulnerable to incorrect claims of geographic origin due to the spatially based management of global fisheries together with a highly globalised seafood market and complex trade network,^1^ leading to a risk of both deliberate fraud for financial gain and accidental mislabelling of a product.

Genetic testing has revealed widespread species substitution and mislabelling across some sectors of the seafood trade network,^5^, ^6^, ^10^ including for whitefish.^11^, ^12^ A review of 51 peer‐reviewed papers investigating seafood species mislabelling using genetic methods including 4500 samples collected globally^5^ found on average 30% of samples tested were mislabelled with regard to species. However, in Europe, the incidence of species mislabelling may have reduced since the widespread use of DNA tests.^6^ A recent meta‐analysis using Bayesian methods suggests the true mislabelling rate is ~8% on average.^13^

Verifying the catch location is much more challenging than testing species' identity,^14^, ^15^, ^16^ because a tracer is required that shows variations between multiple different regions on a suitable spatial scale. EU legislation requires all commercially landed fish to be labelled with the catch location,^17^ but there are no widely accepted forensic tests for spatial origin of seafood products, and no equivalent estimates for the extent of geographic origin mislabelling within retail networks. Current traceability relies mainly on document‐based records at each stage in the supply chain. Vessel monitoring systems are also now used in many countries and have improved traceability at first landing, but these systems are often not used on smaller vessels, and vessel‐based monitoring with paper (or electronic) chain of custody data cannot address accidental or deliberate mis‐association of products to specific vessels.

In marine systems, the stable isotopic composition of structural elements in phytoplankton organic tissues varies spatially due to both variations in the isotopic composition of dissolved inorganic nutrients (e.g. CO_2_, NO_2_, SO_4_), as well as isotopic fractionation associated with nutrient fixation. The spatially varying isotopic composition of primary producers is subsequently transferred through the food chain and becomes incorporated into the tissues of consumers, acting as a natural marker for animals feeding in different locations. Therefore the isotopic composition of food products can be used to differentiate among those from different locations and to infer origin.^18^, ^19^, ^20^

Stable isotope compositions are used extensively to identify origin and movement in humans as well as terrestrial animals and food products.^21^ In marine systems, the increased practical difficulty of obtaining spatially explicit reference samples, together with the relative spatial homogeneity of stable isotope ratios of strontium and oxygen in marine water means that stable isotope ratios have not been widely used to trace the origin of wild‐caught marine food products. To date, isotope‐based studies of spatial origin of wild‐caught seafood have been carried out on sea bass,^22^, ^23^ turbot,^24^ tuna^25^, ^26^ and hake^27^ as well as commercially important invertebrates such as shrimp,^28^, ^29^ scampi (*Nephrops*),^30^ scallops,^31^, ^32^ jumbo squid,^33^ Manila clams^34^ and sea cucumbers.^35^, ^36^ These studies suggest that stable isotope analysis is a promising method for determining origin in some wild‐caught species.

Atlantic cod (*Gadus morhua*, cod from now on) is one of the most commercially important fish species in the northeast Atlantic, with Europe's cod fishery production totalling 1.2 million tonnes in 2020.^37^ Management of cod fisheries and fishery quotas involves several fishery states including Norway, Russia, the Faroes, the United Kingdom and the EU. The United Kingdom imports large amounts of cod, with a value of £425 million in 2021,^38^ mainly caught in the north Norwegian, Barents Sea and Iceland fisheries,^39^ which are certified as sustainable by the Marine Stewardship Council (MSC).^40^ However, several other regional fisheries also supply cod into the market and some are more at risk of overexploitation. The eastern Baltic Sea had a zero recommended catch limit for cod in 2023 since the spawning stock biomass has decreased and the stock status is poor.^41^ North Sea cod was awarded MSC certification in 2017,^40^ but the stock has since deteriorated to below safe biological limits.^42^ Consequently, its MSC certification was suspended in 2019^40^ and the International Council for the Exploration of the Sea (ICES) recommended a significant reduction in the total allowable catch.^42^ Establishing or verifying the catch location of cod in the retail market is of vital importance to the sustainability and future management of cod fisheries. Since Ogden^43^ initiated the concept of using natural tracers for fish forensics in 2008, genetic techniques have been successfully applied to determine the origin of Atlantic cod from several populations on a relatively large spatial scale. Nielsen et al^44^ used single nucleotide polymorphisms (SNPs) to discriminate among Atlantic cod from North Sea, Baltic Sea and northeast Arctic populations, and traced individuals to these *a priori*‐defined populations of origin with 98–100% correct classification. Genetic markers clearly offer a powerful tool for assessing geographic origin among populations that are truly reproductively isolated, but this potential is reduced for species with mixed populations, for fisheries operating at the margins of discrete populations, or fisheries targeting common feeding grounds used by multiple reproductively isolated populations.^43^

Assigning a likely origin to a sample based on a natural tracer involves assessing the relative similarity between the composition of the test sample and reference datasets defining the chemical composition across one of a number of distinct regions.^43^ Known‐origin reference samples must therefore be obtained from each of these regions. Samples can only be assigned to one of the discrete regions contained in the reference dataset, so any test samples originating from outside of these regions will be incorrectly assigned. Any tool proposed to verify claimed catch location or establish catch location at any point through the supply chain must meet accuracy and precision standards. For terrestrial food products, 95% confidence limits are often used for authenticity testing of commercial samples.^45^ Falsely high estimates of accuracy and precision can result if the reference samples do not cover the full range of the fish, the reference regions are decided *a priori* based on expected differences in the tracer used, or if the reference dataset is not independent from the test samples used to assess accuracy.^43^

The aim of the study reported here was to investigate whether the stable isotope composition of Atlantic cod muscle can provide geographic discrimination at a level that has practical use in fisheries management, to compare levels of spatial differentiation expressed in tissue isotope compositions with previously assessed genetic markers and to assess the contribution that stable isotope markers can make as a forensic tool for verifying the spatial origin of traded fish products. We measured the carbon, nitrogen and sulphur stable isotope ratios in 377 Atlantic cod muscle tissue samples of known geographic origin to construct a reference dataset of fish from a wide range of catch locations across the northeast Atlantic. We then used this to determine the accuracy with which individuals can be traced back to their true origin region.

## 2 EXPERIMENTAL

### 2.1 Cod muscle samples

Atlantic cod (*G. morhua*) was chosen for the study due to its very high commercial importance in Europe and around the world, and because it has a wide distributional range from Greenland to North Carolina in the northwest Atlantic and from the Barents Sea to the Bay of Biscay in the northeast Atlantic. It is also a well‐studied species with existing data on the effectiveness of genetic techniques for spatial traceability of cod populations in the northeast Atlantic.^44^

**FIGURE 1:**
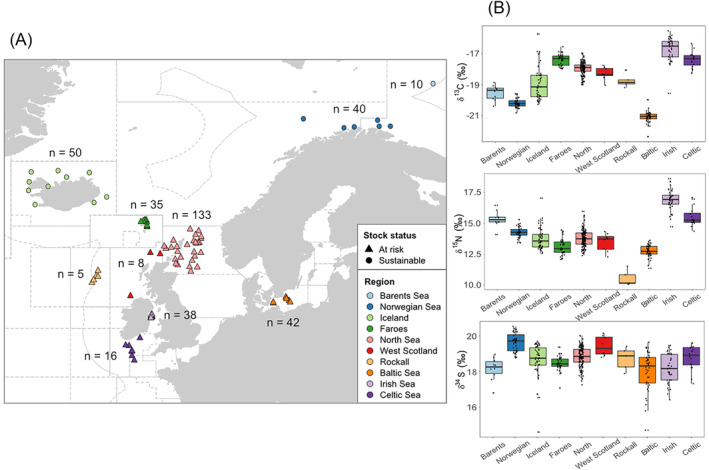
(A) Locations of all stations where samples of Atlantic cod were obtained, coloured according to the geographic regions. The total number of samples (*n*) collected from each region is shown and the symbols indicate whether the stock is sustainable or at risk (as determined from the ICES 2022 advice for each stock,^56^ based on the spawning stock biomass relative to the limit where reproduction to the stock is impaired (B_lim_)). ICES subarea boundaries are indicated by the dotted grey lines. (B) Lipid‐corrected δ^13^C, δ^15^N and δ^34^S values measured in the Atlantic cod muscle tissue samples from each geographic region [Color figure can be viewed at wileyonlinelibrary.com]

Samples of Atlantic cod were collected between February and December 2018 from nine regions in the northeast Atlantic Ocean, and multiple stations were sampled within each region (Figure 1A and Data S3). The sample suite was extended with additional samples provided by Young's Seafood Ltd from the Barents Sea caught in 2017. The sampling regions were selected to cover as many of the main commercial fishery areas as possible over the full geographic range of Atlantic cod, including regions broadly comparable to those sampled by Nielsen et al,^44^ but also based on the availability of known‐origin samples. The regions also correspond to ICES subareas within FAO major fishing area 27. Stations for each region are contained within one subarea, with the exception of the region referred to as the Norwegian Sea which consists of stations close to the Norwegian coast falling either side of the boundary between ICES subareas 1 and 2 (Barents and Norwegian Sea subareas) (Figure 1A).

To ensure authenticity of the catch locations, samples were obtained from research fisheries surveys or fish tagging studies carried out by the relevant research organisation in the area of interest, except those provided by Young's Seafood Ltd. The research organisations and sources who supplied the samples are listed in Table S1.

We aimed to sample at least 50 individuals from each of the regions to estimate the natural variability of wild populations; however, this was not always possible. The total numbers of samples collected from each region are shown in Figure 1A. Sampling from multiple stations ensured that the samples were not all derived from one haul, which may catch individuals travelling as a group and consequently with very similar isotope compositions. Ideally, a maximum of five individuals were sampled from each station, although this was not always possible due to sampling constraints. The body sizes of the fish sampled were not measured, so that they would be representative of typical samples likely to be encountered in the context of traceability. However, only individuals of commercial size were used (>35 cm).

### 2.2 Sample preparation

A small sample of white muscle tissue (approximately 3 cm^3^) was removed from each individual with catch location, species, date collected and station number (haul) recorded. The muscle tissue samples were placed immediately into a −20°C freezer for transport and were kept frozen until processing in the laboratory. The only exception were samples from the Faroe Islands, which were instead preserved in 70% ethanol due to the transportation time back to the United Kingdom.

### 2.3 Stable isotope analysis

The frozen cod muscle samples were freeze‐dried at −55°C for 24 h, and then homogenised into a powder. Care was taken not to include any skin with the muscle tissue. Between 2.5 and 2.8 mg of each powdered sample was weighed into tin capsules for analysis. A total of 377 samples were analysed for bulk carbon, nitrogen and sulphur stable isotope ratios. Most samples were analysed at the Life Sciences Mass Spectrometry Facility (LSMSF) in East Kilbride, United Kingdom, but a minority of samples were measured at the University of Southampton based at the National Oceanography Centre. Samples were analysed at both laboratories using an Elementar vario PYRO cube elemental analyser coupled with an Isoprime visION isotope ratio mass spectrometer. Accuracy and precision were monitored through laboratory internal standards (LSMSF: MSAG, M2 and SAAG2; University of Southampton: sulfanilamide and a protein standard), as well as two common standards run in both laboratories to ensure consistent measurements (fish muscle standard and an in‐house glutamic acid standard). Blanks were also run in each batch. Stable isotope compositions are expressed as δ^13^C, δ^15^N and δ^34^S values in per mille (‰) relative to the international standards (Vienna‐PeeDee Belemnite, air and Vienna‐Canyon Diablo Troilite).

Lipids are depleted in ^13^C compared to proteins and carbohydrates; therefore δ^13^C values in two cod samples with a high lipid content (C:N ratios >3.4) as defined by Skinner et al^46^ were corrected arithmetically using the methodology described by Kiljunen et al.^47^ Samples from the Barents Sea were collected a year prior to all other samples, so the δ^13^C values for these were corrected for the Suess effect by applying a −0.022‰ per year adjustment.^48^

### 2.4 Statistical analysis

All analyses were performed using R version 3.6.2.^49^

Multiple methods of discrete assignment were compared. First, a comparison between isotopic compositions in each ‘unknown’ sample and the reference populations was conducted based on multivariate normal probability distributions. A stratified jack‐knifing approach was applied to estimate the potential accuracy of assignment based on the isotopic separation among potential origin populations. A random subset of 25% of the samples from each location was extracted from the dataset and used as a test subset to be assigned, and the remaining 75% of the samples were used as the reference datasets. The carbon, nitrogen and sulphur stable isotope data for reference samples from each location were used to fit a multivariate normal probability distribution for that location. Each individual from the test subset was assigned to its most likely origin using the multivariate normal probability distributions of the reference dataset. The ‘dmvnorm’ function within the ‘mvtnorm’ package^50^ in R statistical software was used to calculate the probability density function of the multivariate normal distribution and therefore give the likelihoods of the samples having originated from each location. The location with the greatest likelihood is deemed the most likely origin for that sample. This was repeated 1000 times with different random subsets of test and reference data and a mean was calculated for each region (see Data S2 for further details).

The multivariate probability density function used to calculate the likelihoods for multiple variables is defined as:

$$p\left(x\;\right|\;\mu ,\Sigma )=\frac{1}{\sqrt{{\left(2\pi \right)}^{d}}\left|\Sigma \right|}\exp\left(-\frac{1}{2}{\left(x-\mu \right)}^{T}\Sigma^{-1}\left(x-\mu \right)\right)$$

where x is a random vector of size d, μ is the mean vector, Σ is the symmetric, positive definite covariance matrix of size d ×d, ∣Σ∣ is its determinant and T is its transpose.

As a further test of assignment accuracy using this multivariate normal probability method, leave‐one‐out cross‐validation was conducted by removing each sample in turn and assigning them using all the remaining samples.

Two other assignment approaches were compared: linear discriminant analysis and random forest classification. Linear discriminant analysis was performed using the ‘MASS’ package^51^ in R statistical software. A leave‐one‐out approach was applied, where each sample in the dataset was taken as the test sample and assigned to its most likely origin in turn, with all the remaining data used as the reference subset to train the linear discriminant model.

Random forest classification is based on an ensemble of decision trees, and it is a non‐parametric technique that does not require data to be normally distributed. Classification by random forest was conducted using the ‘randomForest’ package^52^ in R statistical software. A leave‐one‐out approach was again applied. The sample sizes were balanced using the ‘sampsize’ parameter within the randomForest package, which draws a specified number of random samples from each group to create the trees, since unbalanced sample sizes may result in the random forest being biased towards groups with a greater number of samples.^53^ The sample size specified using ‘sampsize’ was set as one less than the number of samples in the smallest class in the training dataset (after removing one as the test sample), to allow for random variation among trees, and the same number of samples was drawn from all other regions. The classification was fine‐tuned by selecting the values for ‘ntree’ (number of trees in the random forest) and ‘mtry’ (the number of variables used at each node split) to give the highest accuracy of assignment. The assignment accuracy was determined as the total number of correctly assigned test samples in each region, and this was also reported as a percentage of the total samples. Random forest classification also estimates the error internally. Each tree within the ensemble is created using a different subset of the data at random, where a proportion of the samples are left out and are not used to construct the tree. This approach is used to give an out‐of‐bag error rate.

### 2.5 Assigning independent known‐origin samples

Each of the approaches to estimate potential assignment accuracy described above suffers from non‐independence of training and test samples, which is a common limitation in many assignment studies, and likely results in overestimated assignment accuracy. In an attempt to address this issue, we assembled an independent dataset of carbon and nitrogen stable isotope ratios measured in cod from a range of sources. Published data from a previous study by Jennings and Cogan^54^ contributed isotope ratios measured in cod during a range of years spanning 2002–2010 from the North Sea, Irish Sea and Celtic Sea. A further dataset was provided by Ifremer from the EVHOE 2014 survey,^55^ which includes isotope ratios of cod caught in 2014 and 2015 in the Celtic Sea. These data were combined with results obtained from cod caught in the Barents Sea by the Institute of Marine Research (IMR) in Norway and Icelandic cod provided by Young's Seafood. The literature δ^13^C data were corrected for the Suess effect by applying a −0.022‰ per year adjustment^48^ before being assigned to their most likely location using the bivariate assignment method described previously with the known‐origin cod in the current study as a reference dataset.

## 3 RESULTS

Measurement error associated with the stable isotope analysis of the fish muscle samples determined as the standard deviation of replicate measurements of two internal standards – a fish muscle standard and a glutamic acid standard – was 0.1–0.4‰ for δ^13^C, 0.1–0.2‰ for δ^15^N and 0.6–0.7‰ for δ^34^S. The internal standards used were consistent between laboratories, allowing comparison between the measurements from the two sites. Means of repeated analyses of fish muscle and glutamic acid standards from both laboratories are within one standard deviation of each other (Table S2). The C:N ratios imply that the different storage method for the Faroes samples (70% ethanol prior to freeze drying) had little effect on the stable isotope values, since the C:N ratios are very similar suggesting that minor loss of lipids occurred.

The stable isotope compositions of Atlantic cod muscle samples differ significantly among the 10 regions (ANOVA; δ^13^C: *f* = 187.3, d.f. = 10, *p* < 2 × 10^−16^; δ^15^N: *f* = 119.2, d.f. = 10, *p* < 2 × 10^−16^; δ^34^S: *f* = 15.62, d.f. = 10, *p* < 2 × 10^−16^). The δ^13^C, δ^15^N and δ^34^S values measured in the cod samples from each region are shown in Figures 1B and S1, and the means and standard deviations for each location are listed in Table S3. The full dataset of stable isotope values measured in the cod muscle samples can be found in Data S1. Overall, δ^13^C values range from −15.6‰ to −22.4‰. The lowest mean δ^13^C value of −21.1‰ was found in the Baltic Sea, but the Norwegian Sea samples also have lower δ^13^C values than those in almost all other regions. The greatest variation in δ^13^C values occurs in the Iceland samples, with a range of 4.5‰. The Irish Sea has the highest mean δ^13^C value of −16.7‰ as well as the highest mean δ^15^N value of 16.9‰. The Celtic and Barents Sea samples also have relatively high δ^15^N values, whereas δ^15^N values for the other locations are more similar, apart from cod from Rockall with a much lower mean δ^15^N value. The mean δ^34^S values show less variation among regions than for carbon and nitrogen isotopes, ranging between 18.1‰ and 19.7‰, so values for many regions overlap. However, the δ^34^S values generally have greater variation among individuals within regions than for δ^13^C or δ^15^N, with the largest ranges of 5.1‰ and 4.9‰ for samples from Iceland and the Baltic Sea, respectively. The Norwegian Sea is the most distinctive in terms of sulphur isotope values, since the samples in this region have the highest mean δ^34^S value and the range is relatively small compared to other locations, although the West Scotland samples also have relatively high δ^34^S values.

### 3.1 Grouping of stable isotope data by region

δ^13^C values separate the samples into two main groups (Figure S2). The first consists of the Baltic, Norwegian and Barents Seas with low δ^13^C values due to low salinity (Baltic) and cold temperatures. The Baltic samples form a distinct cluster, which do not overlap with samples from any other locations. The Norwegian and Barents Sea samples show good separation from the remaining regions, but some samples from these two regions are isotopically similar. The remaining warmer, more southerly regions around the Faroes and UK shelf seas yield cod with higher δ^13^C values. Samples from Iceland show the largest spread of δ^13^C values, overlapping both of these groups.

Samples from Rockall are very distinct (Figure S2), with hardly any overlap with the other regions due to the lower δ^15^N values; however, this is based on a small sample size. Other regions are less distinct in their isotopic composition, including the North Sea, Faroes, West Scotland, Celtic Sea and Iceland, which although form clear clusters, show a larger area of overlap in their δ^13^C and δ^15^N values. However, cod caught in the Irish Sea generally had higher δ^15^N values than the other locations and only overlapped with samples from the Celtic Sea, likely due to the close geographic proximity of the two regions.

The δ^34^S values are very variable and overlap in samples from all regions (Figure S2), indicating that sulphur isotopes would have limited use in distinguishing between samples from these locations.

### 3.2 Assignments to catch location

The maximum potential accuracy of assignment for each region of origin using the three techniques – multivariate normal probability distributions, linear discriminant analysis and random forest classification – are compared in Table 1. Random forest classification gave the highest overall accuracy of assignment to origin (mean 79% for all regions), whereas discriminant analysis (Figure S5) and the multivariate normal probability technique both gave similar, slightly lower accuracies: 70% and 72% mean overall, respectively. Each assignment technique performed better for some regions and worse for others, although random forest classification achieved the highest success rate overall as well as for the most individual regions (Figure S6).

**TABLE 1:** Comparison of assignment accuracy for each geographic region using linear discriminant analysis, multivariate normal probability distributions and random forest classification. For linear discriminant analysis, the mean over 1000 repeat simulations is shown. For multivariate analysis and random forest, results are shown using a leave‐one‐out cross‐validation approach

Location	Assignment accuracy (%)		
	Linear discriminant analysis	Multivariate analysis	Random forest
Barents Sea	79	90	90
Norwegian Sea	99	98	90
Iceland	61	70	64
Faroes	74	86	86
North Sea	88	47	52
West Scotland	0	50	50
Rockall	79	40	100
Baltic Sea	93	98	95
Irish Sea	84	82	84
Celtic Sea	42	63	75
Mean accuracy	70	72	79

Cod from the Norwegian and Baltic Seas were the most accurately assigned across each of the assignment methods. The lowest assignment success was obtained for West Scotland, Rockall, North Sea and Celtic Sea, due to a number of cod samples being assigned to nearby regions of the UK shelf sea or Faroes. Detailed breakdowns of the misassignments associated with each classification approach are provided in Figure S3 and Tables S5, S6 and S7. Most often the incorrect assignments were to the nearest region geographically, such as North Sea samples being assigned to the Faroes or West Scotland, or Irish Sea samples being assigned to the Celtic Sea, since the region in closest proximity is often the most isotopically similar.

δ^34^S values contributed little to the spatial traceability of cod in the regions studied. However, sulphur had more potential informational value for Icelandic cod, for which the assignment accuracy increased from 59% to 73% when δ^34^S was included as an assignment tracer using the multivariate method (Table S4). The ability to distinguish between Iceland and West Scotland samples was particularly improved, since over 50% of Icelandic cod were assigned to West Scotland in some simulations using only carbon and nitrogen isotopes compared with a mean of 2% over all repeat simulations with the addition of sulphur isotopes (Figures S3 and S4). Therefore, in limited cases sulphur isotopes gave an improvement in assignment accuracy, but for most regions sulphur did not provide significantly greater distinguishing power than using carbon and nitrogen isotopes alone.

### 3.3 Assigning independent known‐origin samples to the reference dataset

Isotope data from fully independently collected test samples (Jennings and Cogan,^54^ IMR (Norway), Young's Seafood Ltd and Ifremer^55^) overlapped with our reference data (Figure S7), but did not include samples from the isotopically distinct Baltic region. The assignment successes obtained from fully independent test samples were reduced for all regions compared to the estimates calculated using only the samples from the current study (non‐independent samples; Table 2).

**TABLE 2:** Results of assigning cod samples measured in previous datasets to the sample data collected in our study as a reference dataset (with a correction for the Suess effect on δ^13^C values), using only δ^13^C and δ^15^N. The correct assignments for each region are highlighted in blue. Previous datasets: Barents Sea – collected by IMR (Norway); Iceland – provided by Young's Seafood Ltd; North Sea – from Jennings and Cogan^54^; Irish Sea – from Jennings and Cogan^54^; Celtic Sea – from Jennings and Cogan^54^ and collected by Ifremer^55^ from the EVHOE 2014 survey

True origin region	Assigned region	Cod assigned (count)	Cod assigned (%)
Barents	Barents Sea	2	2
	Norwegian Sea	63	73
	Iceland	3	4
	Faroes	0	0
	North Sea	0	0
	West Scotland	0	0
	Rockall	0	0
	Baltic Sea	18	21
	Irish Sea	0	0
	Celtic Sea	0	0
Iceland	Barents Sea	0	0
	Norwegian Sea	1	10
	Iceland	5	50
	Faroes	1	10
	North Sea	3	30
	West Scotland	0	0
	Rockall	0	0
	Baltic Sea	0	0
	Irish Sea	0	0
	Celtic Sea	0	0
North Sea	Barents Sea	0	0
	Norwegian Sea	0	0
	Iceland	18	12
	Faroes	31	20
	North Sea	19	12
	West Scotland	64	42
	Rockall	18	12
	Baltic Sea	2	1
	Irish Sea	0	0
	Celtic Sea	2	1
Irish Sea	Barents Sea	5	15
	Norwegian Sea	0	0
	Iceland	1	3
	Faroes	3	
	North Sea	3	9
	West Scotland	3	9
	Rockall	0	0
	Baltic Sea	0	0
	Irish Sea	9	27
	Celtic Sea	10	29
Celtic Sea	Barents Sea	0	0
	Norwegian Sea	0	0
	Iceland	1	1
	Faroes	8	11
	North Sea	25	35
	West Scotland	3	4
	Rockall	0	0
	Baltic Sea	0	0
	Irish Sea	9	13
	Celtic Sea	25	35

Assignment accuracies for individual regions decreased the least for Iceland (from 64% to 50%) and the most for the Irish Sea (from 84% to 27%) using independent samples, excluding the Barents Sea samples which were mostly assigned to the Norwegian Sea (Table 2). It is possible that these samples originated in the north Norwegian Sea rather than central Barents Sea, since we do not have a record of the exact catch locations. The reduced assignment successes using independent samples emphasises the importance of independent sampling to determine the realistic assignment accuracy potential.

Further details of the results are available in the supplementary materials (Data S2).

## 4 DISCUSSION

The results of this study demonstrate that stable isotope compositions of Atlantic cod muscle differ systematically among major fishery areas, highlighting the potential use of stable isotopes as a forensic tool to indicate provenance of Atlantic cod products. Cod from the Barents Sea, Norwegian Sea, Baltic Sea and Rockall could be traced to their catch area with high confidence (90–100%), whereas other regions were less isotopically distinct, such as Iceland, the North Sea and West Scotland, which resulted in lower assignment accuracies (64%, 52% and 50%, respectively). The Baltic Sea samples were one of the most reliably discriminated, as is expected due to the different environmental conditions – the Baltic Sea is brackish because of freshwater runoff and has a shallow sill at the entrance to the Atlantic Ocean resulting in limited water exchange.

### 4.1 Comparison of assignment methods

Random forest classification performed the best overall for assignment accuracy to region of origin, achieving a significantly higher success rate than the other methods for several regions. The multivariate normal probability technique and linear discriminant analysis gave very similar assignment accuracies overall. Although these two techniques did not have such a high overall success rate as random forest classification, they had the advantage of performing better for certain discrete regions. Cross‐validation gave very similar results to the multivariate technique, since the same multivariate assignment method was used, but for regions with a small sample size this sometimes led to a higher assignment accuracy because fewer samples are removed at a time, leaving a larger reference subset to assign the test sample against. Therefore, random forest classification appears to be the best overall choice of assignment method for this dataset and may improve further if more balanced sample sizes were obtained, but all approaches gave relatively similar results.

Sulphur stable isotope ratios have rarely been used in the marine environment for tracing the origin of animals, but they have been found to increase the ability to distinguish among catch locations of scampi (*Nephrops norvegicus*).^30^ In the current study, the inclusion of sulphur isotope ratios did not increase the proportion of cod correctly traced to origin for the majority of fishery regions. In fact, the addition of sulphur isotope measurements reduced the assignment accuracy in many cases due to the large and overlapping ranges of δ^34^S values in cod from almost all areas. Of all the regions analysed, only Icelandic cod benefitted significantly from including sulphur isotopes, with an increase in assignment accuracy from 59% using only carbon and nitrogen isotopes to 73% when sulphur isotopes were added.

### 4.2 Comparison of stable isotope analysis with genetic techniques applied to restricted, pre‐selected populations

Nielsen et al^44^ used SNPs to assign Atlantic cod to three populations of origin in the North Atlantic. In the current study using stable isotope ratios, if the same cod populations as in Nielsen et al^44^ are selected from the dataset and all other sampled locations excluded (combining the Barents and Norwegian samples into one group – the northeast Arctic), the assignment accuracy using the same multivariate assignment methodology as previously described is 100% for the northeast Arctic, 99% for the North Sea and 94% for the Baltic Sea, and therefore very close to the high success rates achieved using genetic techniques for the same regions (98–100%). Using either the genetic or isotopic techniques, the Baltic Sea had the lowest assignment accuracy of the three regions. For the genetic assignments, the likelihood of the test sample originating from the true population of origin was at least six times higher than for the second most likely population of origin.^44^ In our study using stable isotopes, the majority of assignments also had a probability at least six times greater for the true region of origin than the second most likely region – only between zero and eight samples out of a total of 82 test samples had a probability less than six times greater, varying with repeat simulations using different test subsets. If only ‘strong’ assignments are included, where the likelihood of the true location is at least six times greater than the second most likely origin, and others are discarded, the assignment accuracy increases to 100% for the northeast Arctic, 99% for the North Sea and 96% for the Baltic Sea. Furthermore, using random forest classification, high assignment accuracies of 94%, 100% and 100% for the northeast Arctic, North Sea and Baltic Sea were achieved, respectively. Only three samples were misclassified in total, all from the northeast Arctic. Therefore, it is demonstrated that when datasets are reduced to include only samples from highly distinct populations, isotopic and genetic assignment techniques perform similarly well. It is likely that combining the two approaches would reduce errors and give a higher confidence in tracing provenance than either technique used in isolation.^16^

### 4.3 Assignment of independent known‐origin samples

Apparent assignment potential is inflated when estimating accuracy from the same reference samples used to define classification rules. Assigning samples based on independent sampling resulted in reduced apparent assignment success in every region. This lower assignment accuracy compared to assigning non‐independent samples reflects a combination of additional variance effects associated with (but not limited to) location, time and biology of the fish, all of which are uncontrolled in any true forensic test. Therefore, the jack‐knifing or leave‐one‐out approach gives the maximum possible discrimination, whereas the independent samples test the realistic assignment potential.

Temporal variation in the spatial distribution of δ^13^C and δ^15^N over seasonal and yearly timescales results from changes in the rate of hydrodynamic and biogeochemical processes with time,^18^ which is then transferred up the food web. The independent samples from the Barents Sea and Icelandic waters were collected 1 year prior to those in the current study so the cod from these regions show similar isotopic compositions in both datasets (assuming the Barents Sea samples were actually collected in the north Norwegian Sea as discussed previously), whereas the Jennings and Cogan^54^ samples were collected 8–16 years earlier than in this study, so for these the temporal effect on assignment accuracy was more significant. Matching the timescale of reference sample collection to that of the unknown samples is likely to result in more reliable assignments. The difference between the datasets could also partly be attributed to the differences in the exact locations of sampling within the same region. The samples in Jennings and Cogan^54^ from the North and Celtic Seas were collected from similar locations to our reference samples, but those from the Irish Sea cover a much wider area (Figure S8). Creating a reference dataset using samples collected from across the whole of each target region better encompasses the true variance for the region and would likely result in a higher assignment accuracy.

### 4.4 Limitations and future research

Ogden^43^ laid out the considerations of moving genetic tools from fisheries research to fish forensics. Most considerations also apply to stable isotopes. Ogden^43^ particularly draws attention to the requirement that reference samples are to be independently derived from the test or assignment samples.

There are limitations in the use of stable isotope ratios for provenance traceability related to the movement of individuals over time. If an individual migrates across multiple regions, the isotopic signature will be integrated across these boundaries and the results will be difficult to interpret. This is particularly a consideration for animals where the tissue turnover rate (time taken to assimilate the isotope values of the diet into tissue) is slow relative to the migratory movements of the animal. In this case, assignment to origin will be difficult because the individual moves across isotopic gradients faster than new tissue is formed. Likewise, the location at which an animal feeds may not necessarily be the location where it is caught for very mobile animals, or animals could be feeding on mobile prey that have themselves crossed isotope gradients. Most cod populations in the northeast Atlantic form regionally discrete populations,^57^ but other species with less geographically structured populations would be less successfully assigned to origin using stable isotopes. Temporal variations in the isotopic composition of plankton at the base of the food web could lead to seasonal variations in fish tissue. However, most production in marine food webs is fuelled from seasonal plankton growth, and fish in temperate regions have correspondingly distinct growth seasons. Furthermore, muscle is a relatively slow‐turnover tissue averaging over several months. It is therefore likely that seasonal variations in plankton baselines are significantly dampened in cod muscle.

Marine food webs are commonly structured by size, and tissue δ^15^N and δ^13^C values typically increase systematically with body size.^58^, ^59^ In order to infer location from the isotopic compositions of fish of different species or body sizes a correction is necessary to account for trophic level and/or body size effects, but this can be difficult to determine and introduces additional uncertainty.^60^ In this study, body size was not recorded (although all fish were of commercial size) since the aim was to develop a tool that could be used in trade or retail situations, where the size before processing is unlikely to be known. Fish of a range of sizes were sampled from all regions, so that the isotopic variation due to body size was taken into account. However, it is possible that errors could occur if an individual outside the size range of the reference dataset is assigned to origin.

There are also several advantages of using stable isotope techniques for establishing origin of fish. They can be used on uncooked processed products (muscle tissue), being mindful of contamination with other added ingredients. Furthermore, sampling and storage requirements are minimal – it is possible to collect samples from markets or auctions and muscle samples can be frozen or dried within a day or two of collection.

In this study, discrete assignments were used to determine the most likely origin. Discrete assignments require a reference dataset of tissue isotope values from each of the potential origin areas. Therefore, discrete assignments assume that all possible origin regions have been sampled and are part of the reference dataset. Any test individuals from an origin not contained in the reference dataset will be incorrectly assigned. The accuracy of assignment also relies on the reference dataset sufficiently encompassing the true variance of the individuals from that region. To further test the use of stable isotopes for tracing the provenance of Atlantic cod, the regions sampled in this study could be expanded to cover all possible catch regions on a spatial scale relevant to ICES fishery areas, and for some regions already sampled to increase the sample numbers to ensure the true variance has been sampled. Due to the temporal variation in stable isotope ratios, the reference dataset should also have been acquired during a similar timeframe as the test samples if they are to be reliably compared, since products from the same location could show different isotopic compositions if they were collected in different years or seasons. For terrestrial food products, new reference data are collected each year from the different geographic regions.^45^ However, for marine food products with large latitudinal harvesting ranges, baselines are unlikely to need frequent resampling. For example, MacKenzie et al^61^ have shown that regional marine carbon and nitrogen isoscapes are stable over at least 10–15 years.

Considerable effort is required to build reference datasets for tracer‐based spatial assignment. Predicting whether isotope methods are likely to be effective before committing resources to sampling would be a considerable advantage. Isotope‐enabled global biogeochemical models can provide *a priori* estimates of which fishery areas are likely to be isotopically distinct, and therefore where isotope markers may have predictive value.^16^ Cusa et al^16^ predicted the ability to discriminate among Atlantic cod from MSC certified fishery areas based on isotope‐enabled global biogeochemical models, and found that cod from the Barents Sea, North Sea and Icelandic waters could be assigned to origin with predicted accuracies of 90%, 89% and 50%, respectively. Here we corroborate the high assignment potential for Barents Sea cod, but achieved lower assignment accuracy for North Sea cod and higher for Icelandic cod.

## 5 CONCLUSIONS

Samples of Atlantic cod muscle from the northeast Atlantic could be assigned to known fishery origin based on stable isotope analyses, but the accuracy achievable is highly sensitive to the locations considered. Assignment accuracies approaching 100% can be achieved when comparing between fisheries with clear isotopic separation (e.g. between the Barents and Baltic populations), but isotope methods alone cannot effectively differentiate among cod fisheries surrounding the British Isles.

Apparent assignment accuracies are strongly influenced by dependence of reference and test samples. Samples analysed to assess the accuracy of forensic assignment approaches should be collected independently from those used to define reference datasets.

Stable isotope analyses offer cost‐effective tools to assist forensic assessments of catch origin for Atlantic cod, but their use requires careful consideration of the provenance question asked.

## AUTHOR CONTRIBUTIONS

**Juliet S.E. Wilson:** Conceptualization; data curation; formal analysis; investigation; methodology; project administration; visualization; writing—review and editing; writing—original draft; funding acquisition. **Rona A.R. McGill:** Investigation; methodology; supervision; validation; writing—review and editing. **Petur Steingrund:** Writing—review and editing; resources. **Clive N. Trueman:** Conceptualization; funding acquisition; methodology; supervision; writing—review and editing.

### PEER REVIEW

The peer review history for this article is available at https://www.webofscience.com/api/gateway/wos/peer-review/10.1002/rcm.9861.

## Supporting information


**Data S1.** Supporting Information

**Figure S1** Frequency distributions of δ^13^C, δ^15^N and δ^34^S values from Atlantic cod caught within each of the ten sampled regions.

**Figure S2** Carbon, nitrogen and sulfur stable isotope values for each individual cod sampled, coloured by region of origin. The 90% data ellipses are also shown for each geographic region.

**Figure S3** Assignment results using carbon, nitrogen and sulfur stable isotope data, showing the percentage of individuals from each known location assigned to all the possible regions over 1000 repeat simulations. The coloured boxes show the correct regions of origin.

**Figure S4** Assignment results using only carbon and nitrogen stable isotope data, showing the percentage of individuals from each known location assigned to all the possible regions over 1000 repeat simulations. The coloured boxes show the correct regions of origin.

**Figure S5** Linear discriminant analysis (LD1 and LD2) using the carbon, nitrogen and sulfur stable isotope compositions measured in cod muscle tissue from each of the sampled regions.

**Figure S6** Locations of sampling stations within ICES sub area boundaries (grey lines), and assignment success rates in each region using random forest classification with a leave‐one‐out cross validation approach.

**Figure S7** Carbon and nitrogen stable isotope values measured in cod samples collected in this study (reference samples) compared with those collected from previous years and studies (test samples), after applying Suess correction on δ^13^C values. Previous data: Barents 2017 – collected by Institute of Marine Research (Norway); Iceland 2017 – provided by Young's Seafood Ltd.; North Sea 2002‐2006 – from Jennings and Cogan^54^; Irish Sea 2010 – from Jennings and Cogan^54^; Celtic Sea 2010 – from Jennings and Cogan^54^, Celtic Sea 2014‐2015 – collected by Ifremer^55^ from the EVHOE 2014 survey.

**Figure S8** Locations where independent cod samples were collected previously by Jennings and Cogan^54^ compared to the locations where samples were collected for the current study in 2018 in the corresponding regions.

**Data S2.** Supporting Information

**Data S3.** Supporting Information

**Table S1** Total number of individual Atlantic cod collected from each geographic region, the number of stations sampled per region, and the source from which samples were obtained.

**Table S2** Comparison of the mean values and uncertainties (standard deviations) in the stable isotope measurements of two internal standards measured at both laboratories where samples were analysed.

**Table S3** Means and standard deviations of carbon (lipid corrected), nitrogen and sulfur stable isotope ratios from Atlantic cod caught in each of the sampled geographic regions.

**Table S4** Mean percentage of individuals assigned to the correct origin region over
1000 repeat simulations using three isotopes (δ^13^C, δ^15^N and δ^34^S) and two isotopes
(δ^13^C and δ^15^N).

**Table S5** Leave‐one‐out cross validation results using the multivariate normal probability method, showing the number of samples assigned to each of the geographic regions as well as the percentage of correct assignments for each region. True known origins are shown in the columns and the assigned most likely origins are shown in the rows.

**Table S6** Assignment results to each of the geographic regions using linear discriminant analysis, showing the mean percentage over 1000 repeat simulations. The correct assignments for each region are shown in bold.

**Table S7** Leave‐one‐out cross validation results using random forest classification, showing the number of samples assigned to each of the geographic regions as well as the percentage of correct assignments for each region shown in bold.

## Data Availability

The data that support the findings of this study are available in the supplementary materials of this article.
