# Supplementary figures and images for: Tracing the geographic origin of Atlantic cod products using stable isotope analysis
Source: Rapid Commun Mass Spectrom. 2024 Jul 22;39(Suppl 1):e9861. doi: 10.1002/rcm.9861 (PMC12062778; doi:10.1002/rcm.9861)

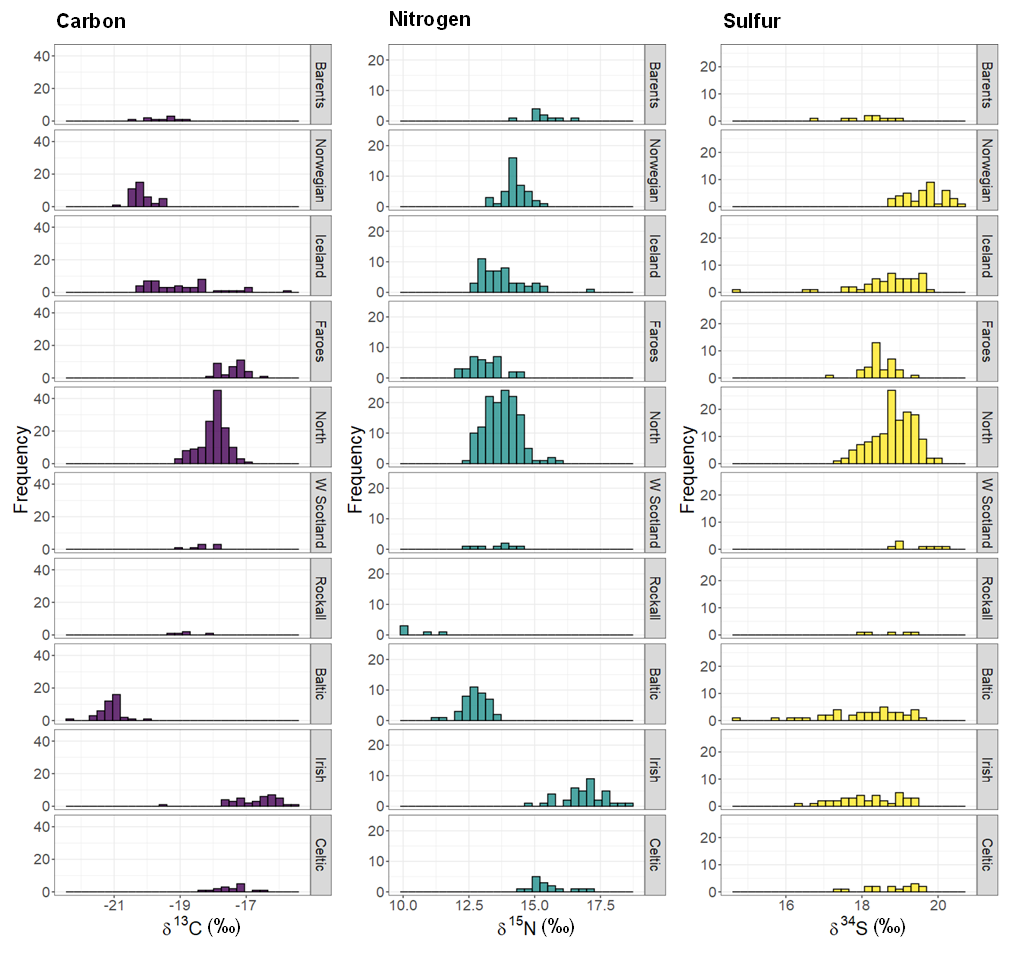

Supplement: Supplementary file 2 — Figure S1 Frequency distributions of δ13C, δ15N and δ34S values from Atlantic cod caught within each of the ten sampled regions. [file RCM-39-e9861-s017.png]

### Carbon and Nitrogen

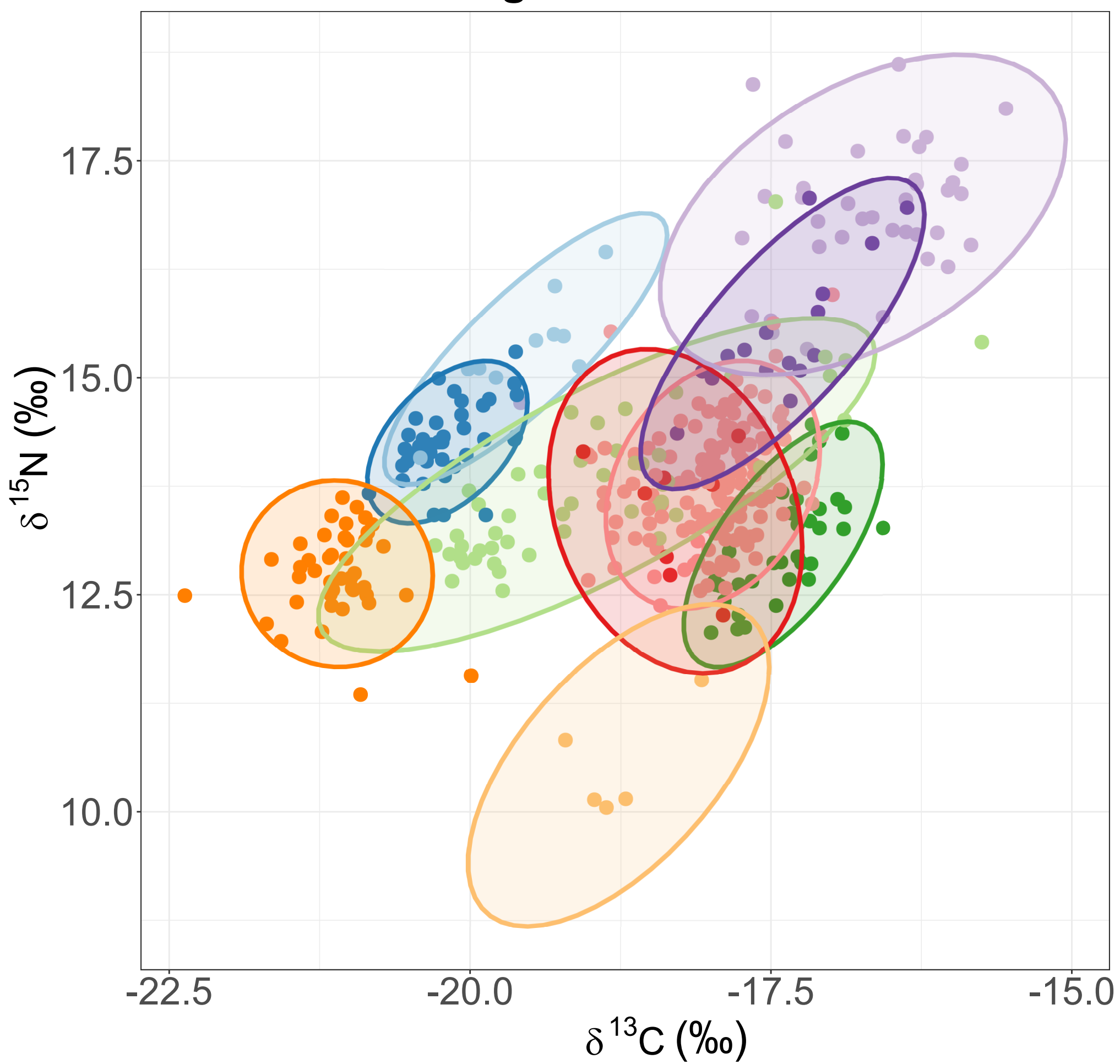

### Carbon and Sulfur

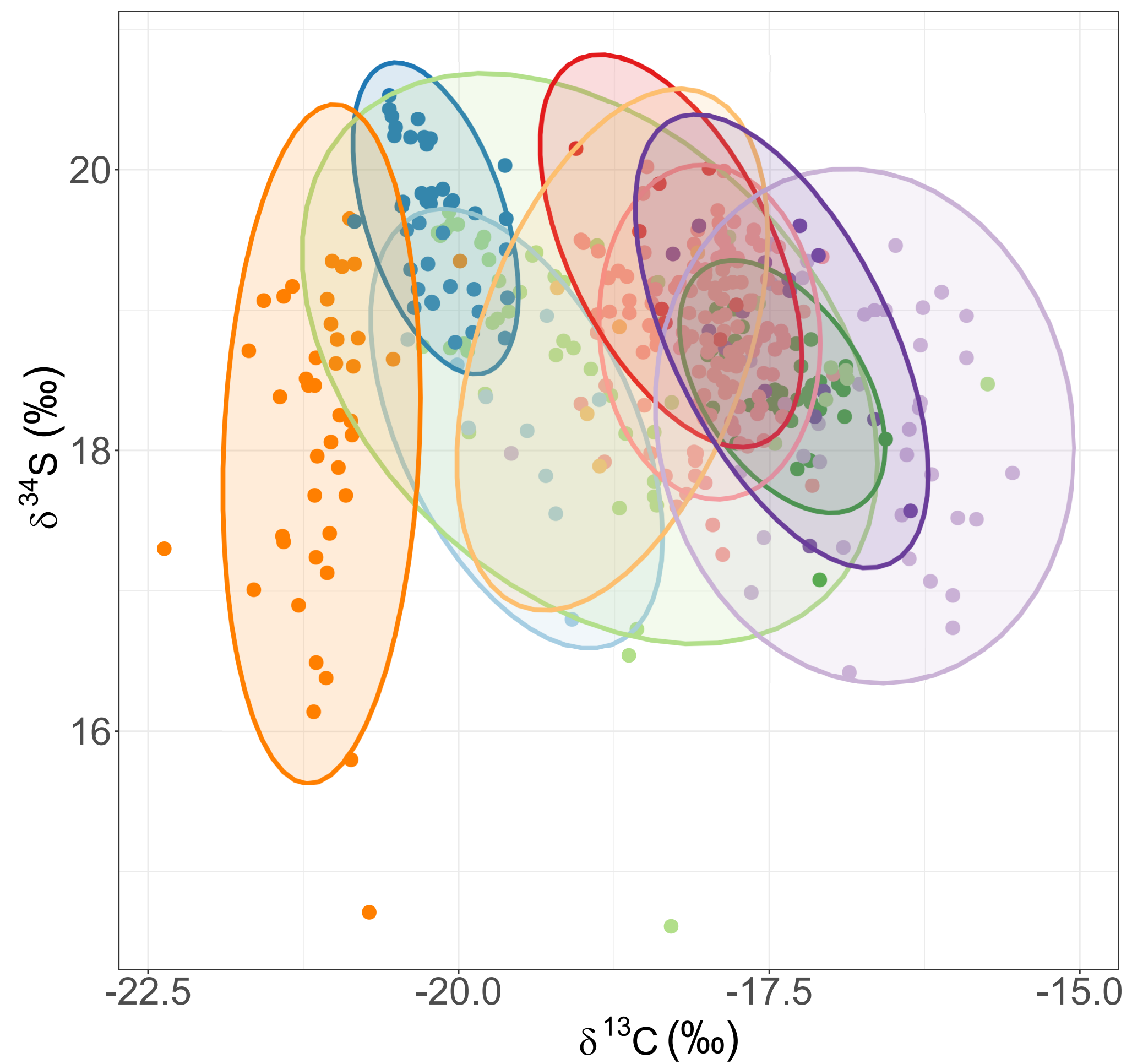

### Nitrogen and Sulfur

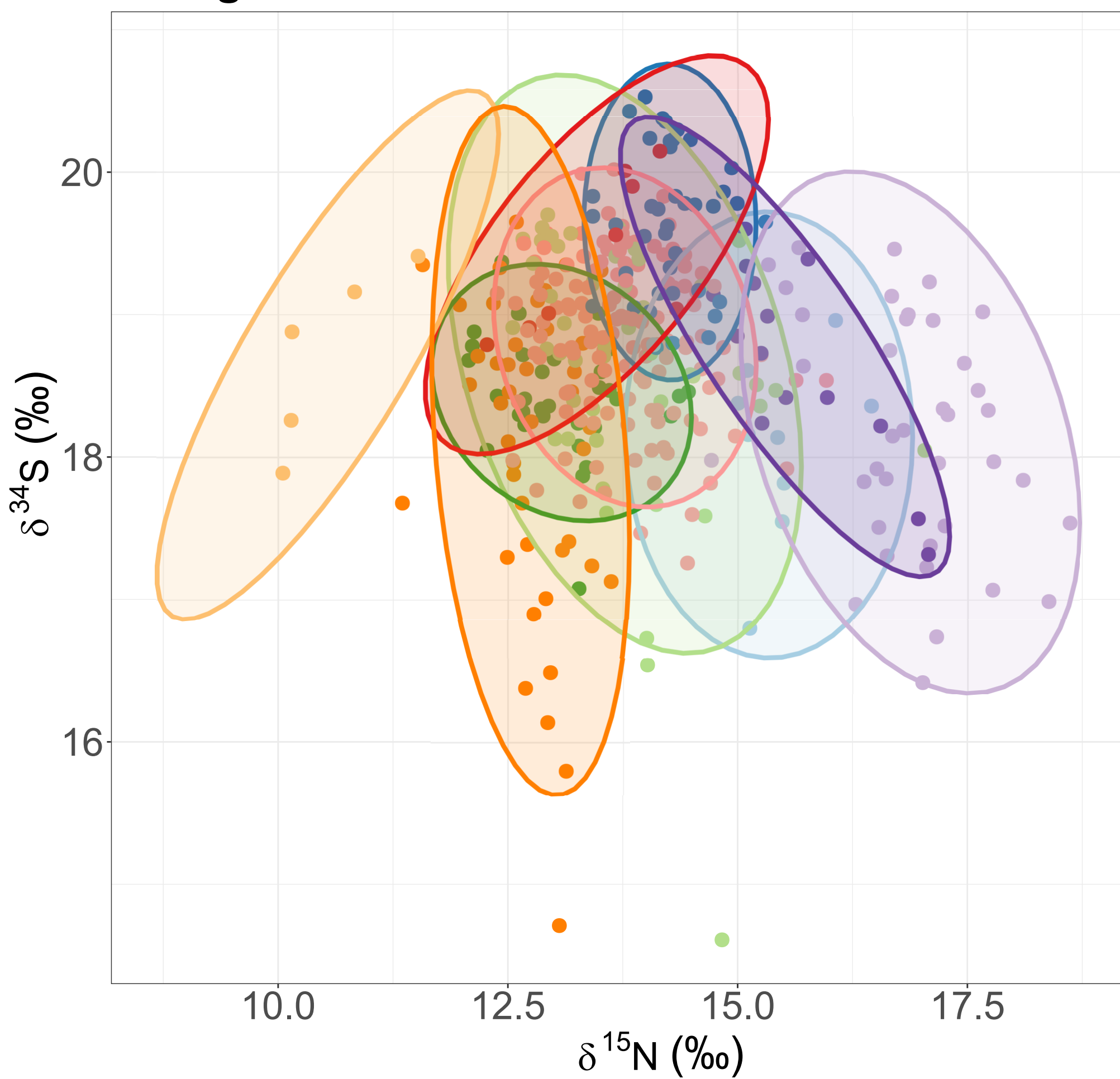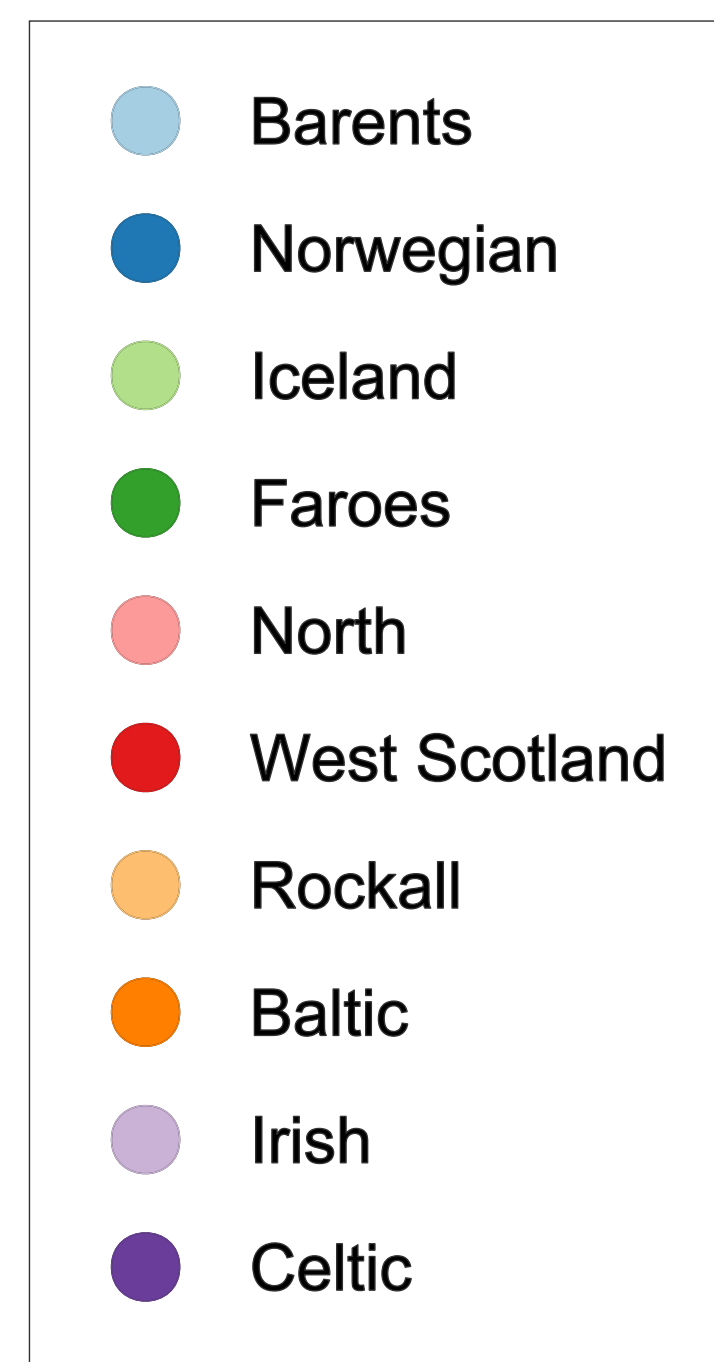

Supplement: Supplementary file 3 — Figure S2 Carbon, nitrogen and sulfur stable isotope values for each individual cod sampled, coloured by region of origin. The 90% data ellipses are also shown for each geographic region. [file RCM-39-e9861-s004.pdf]

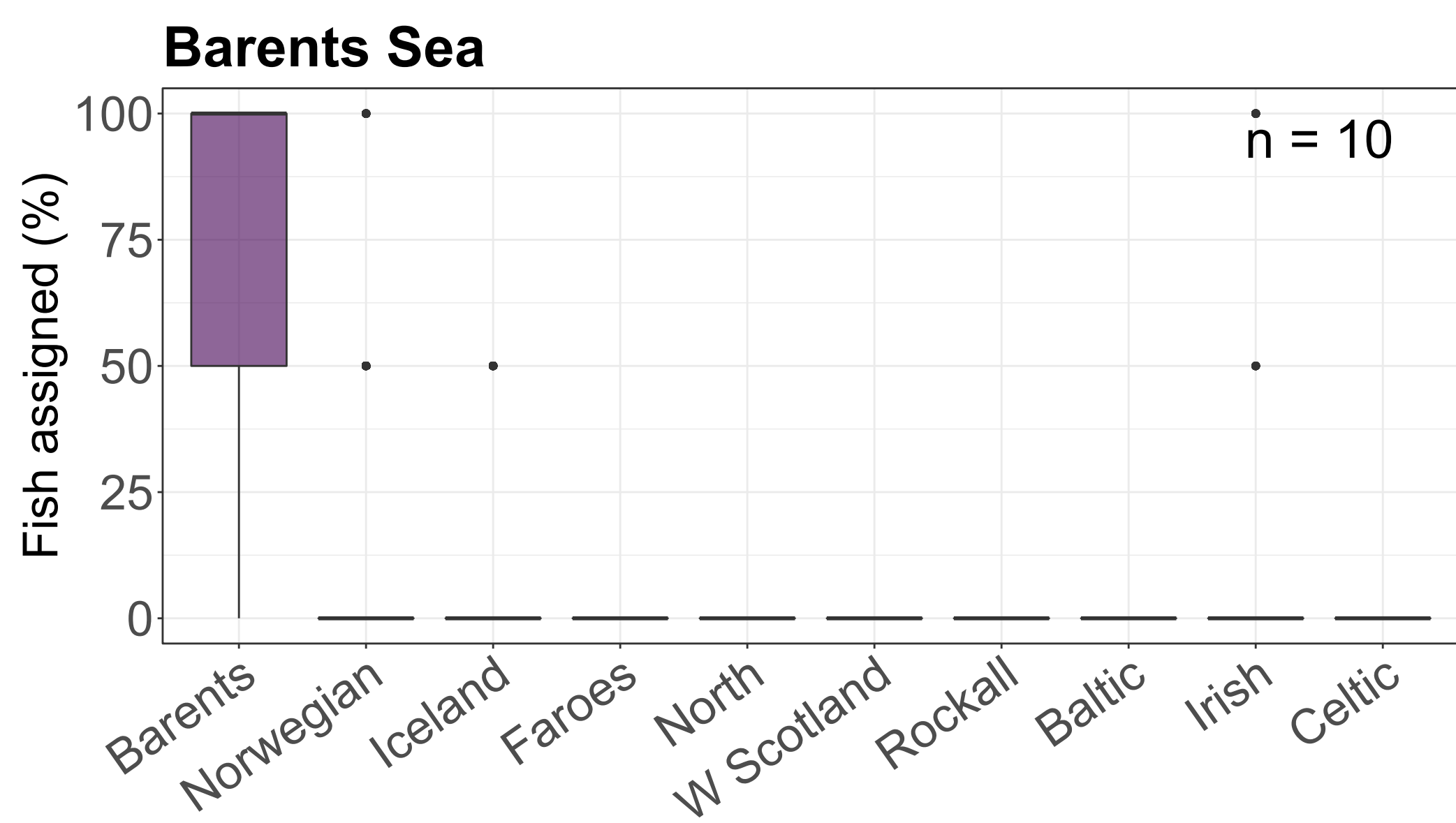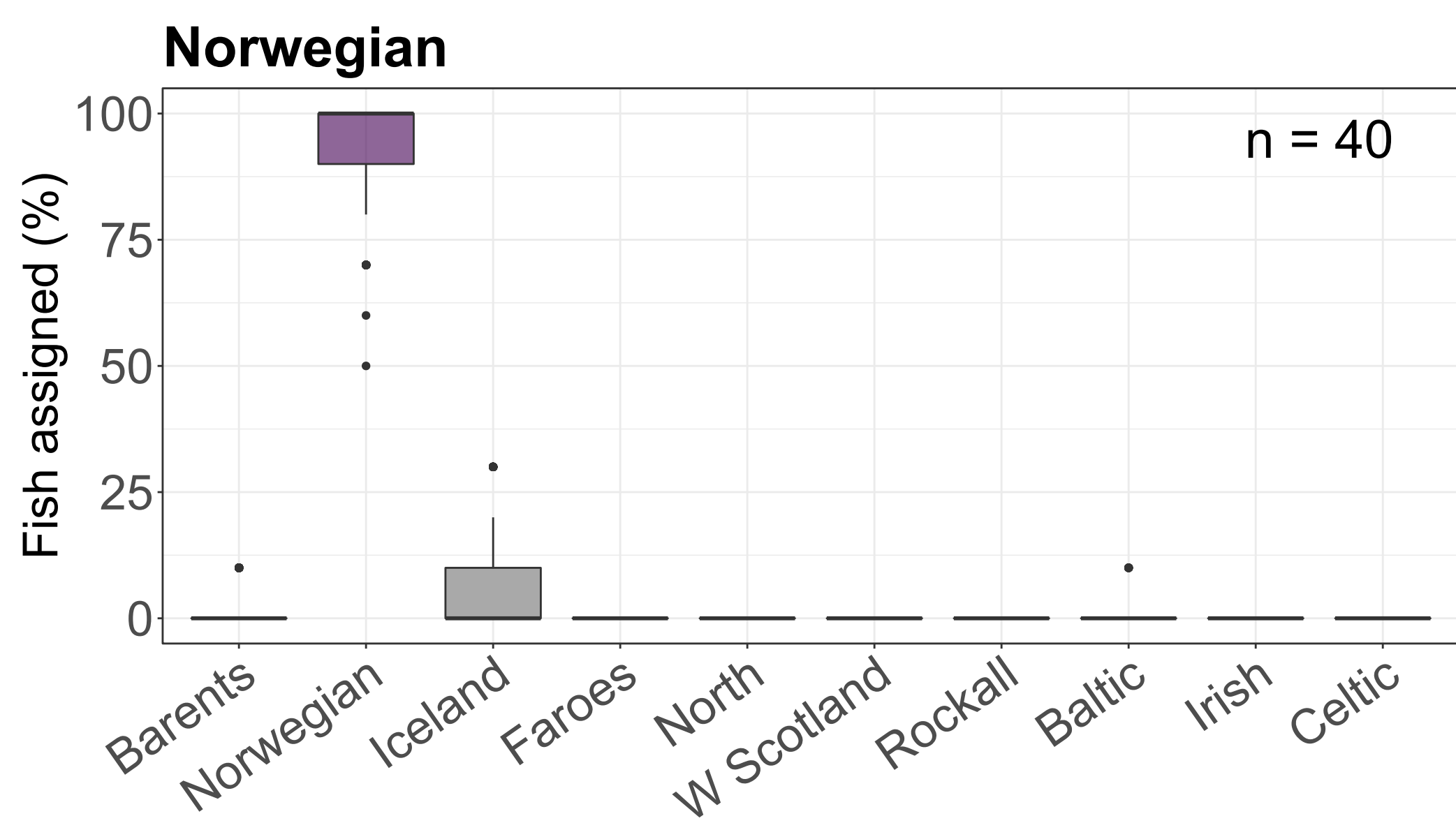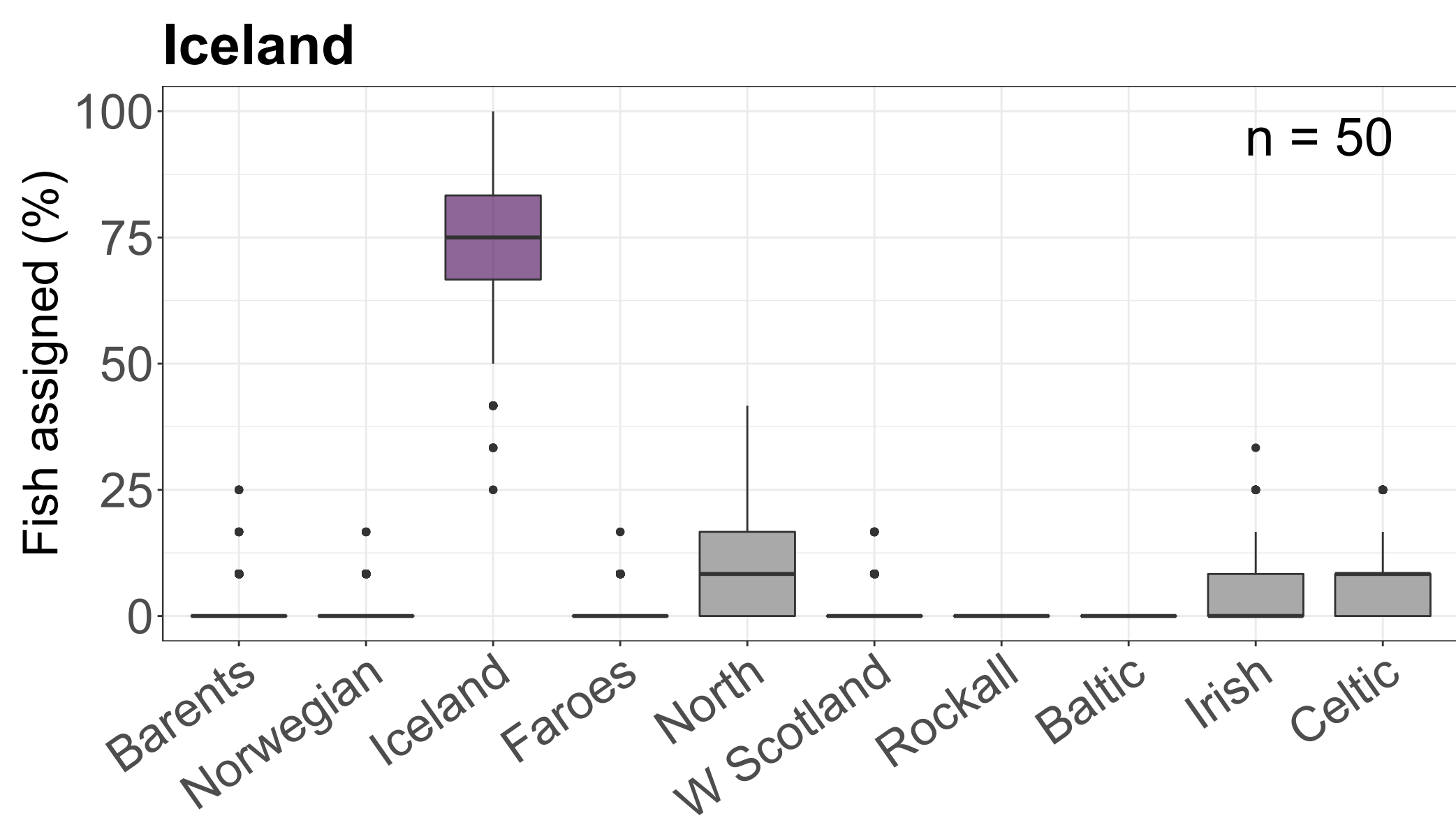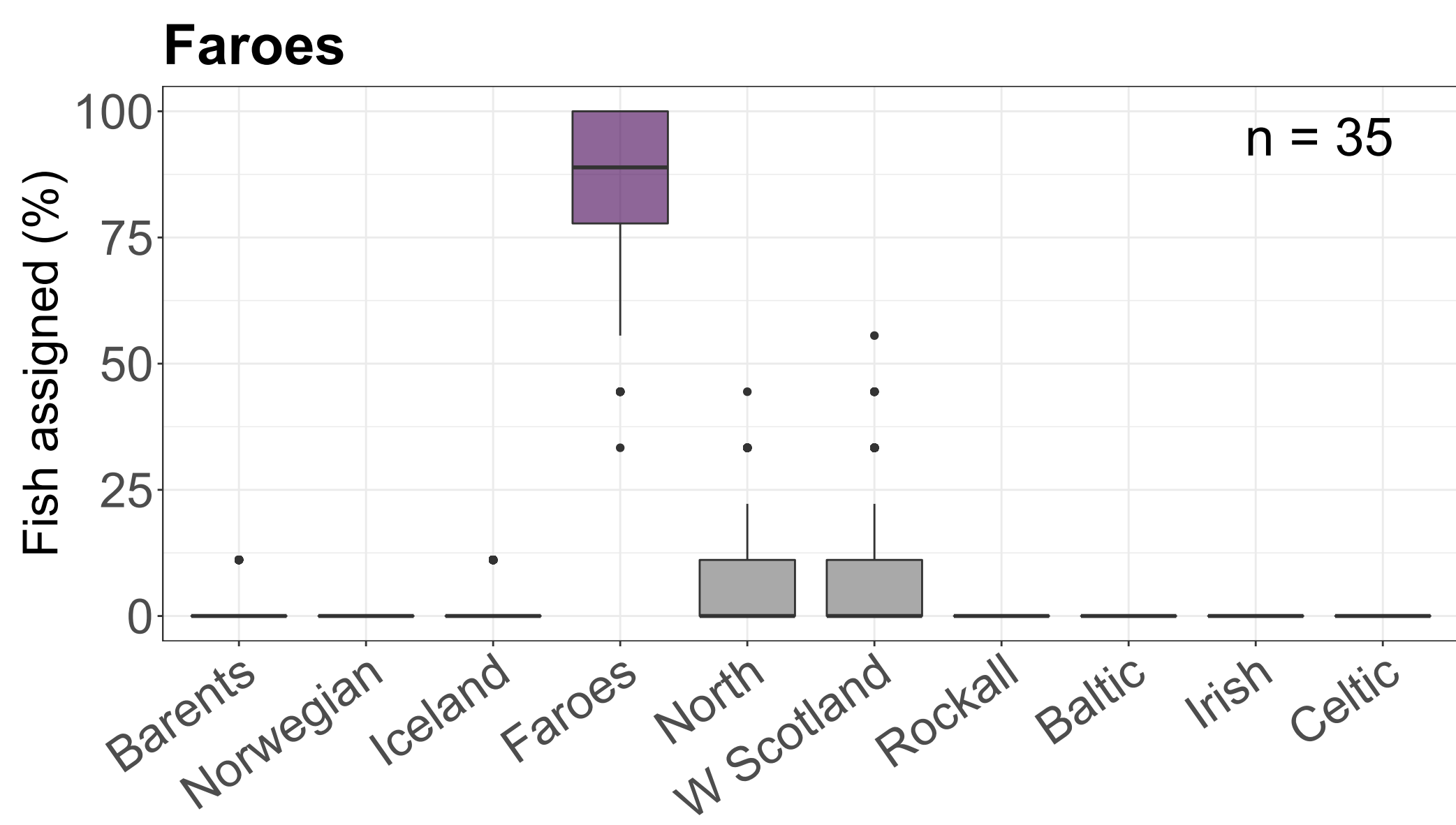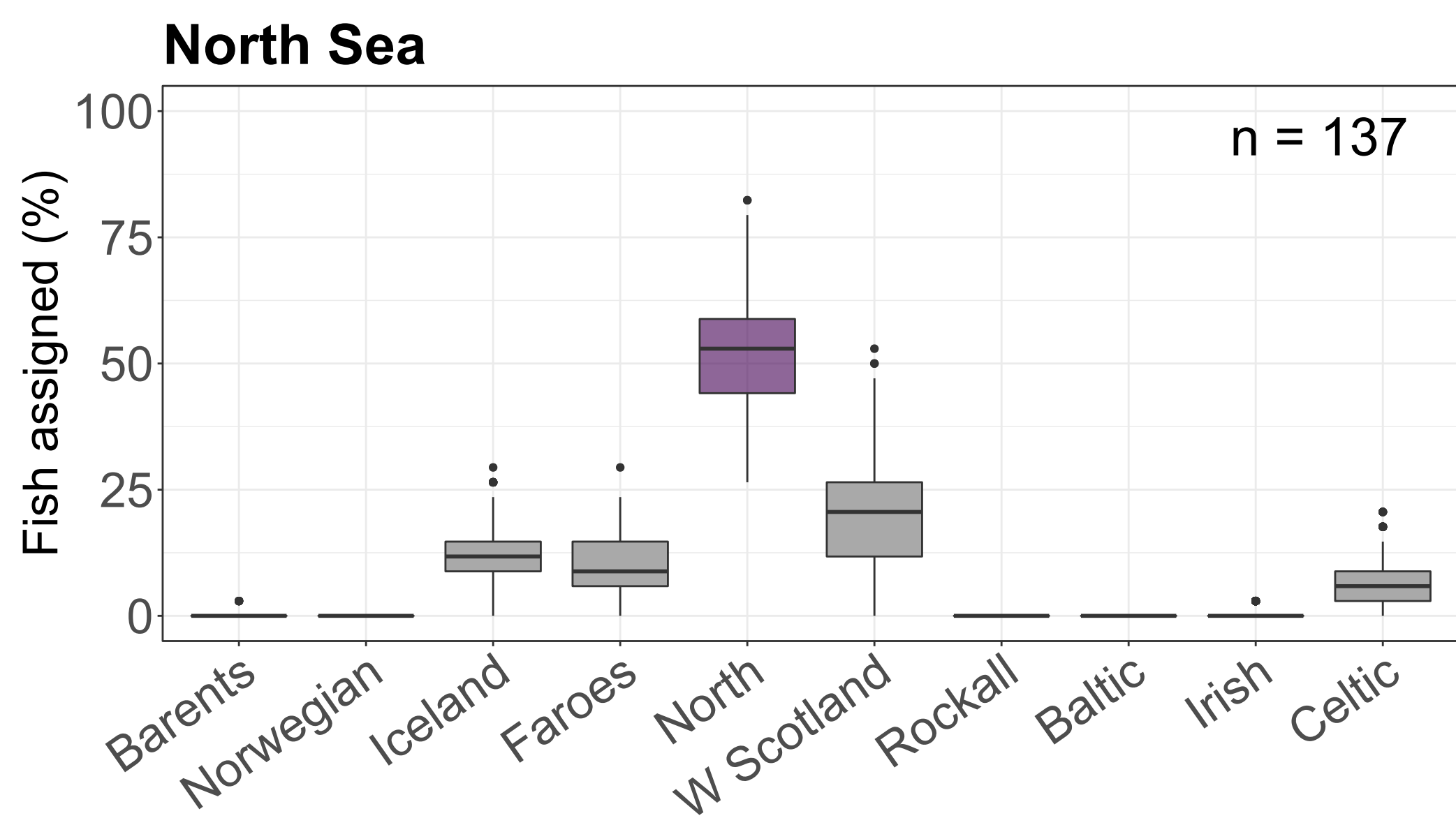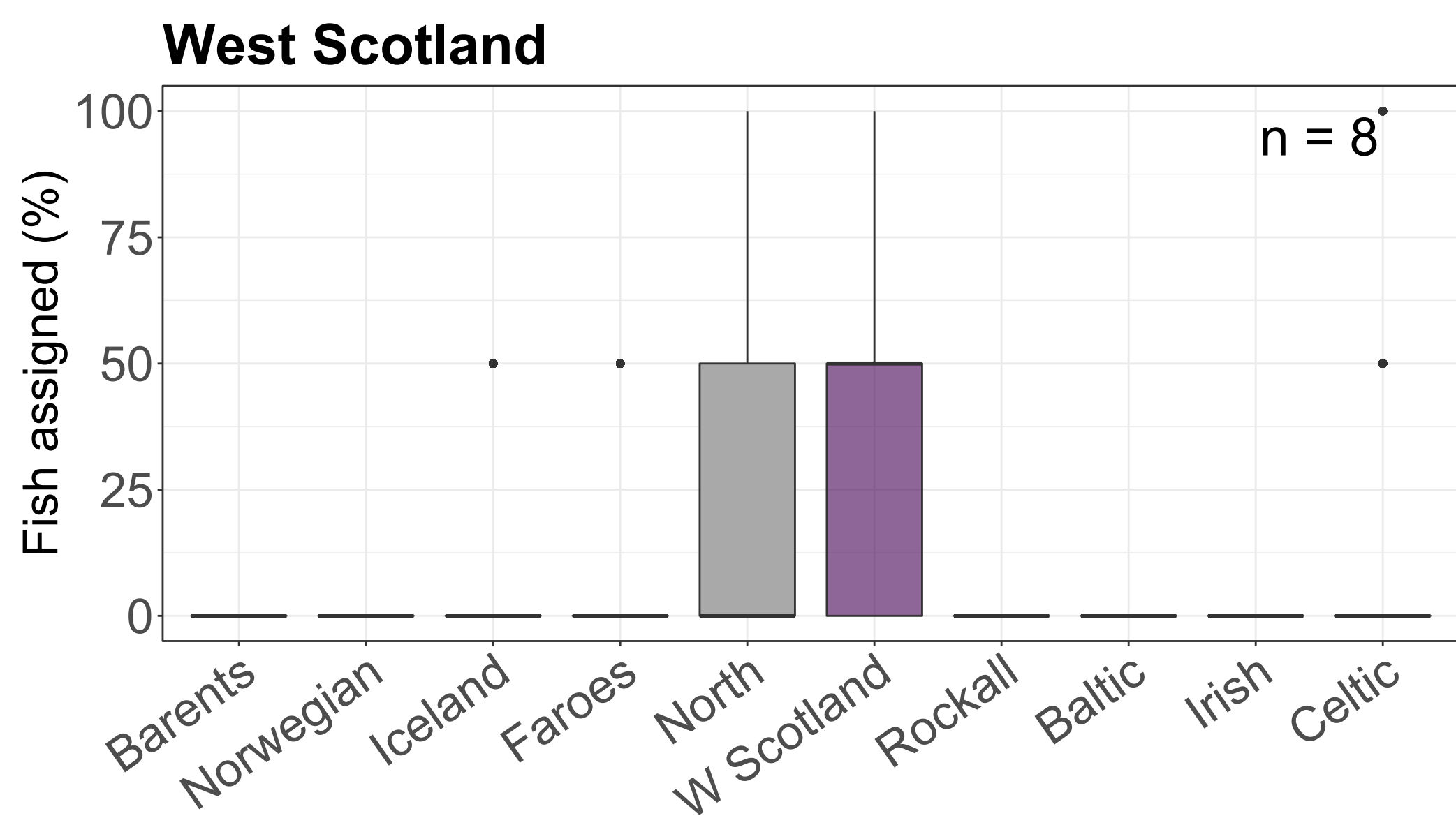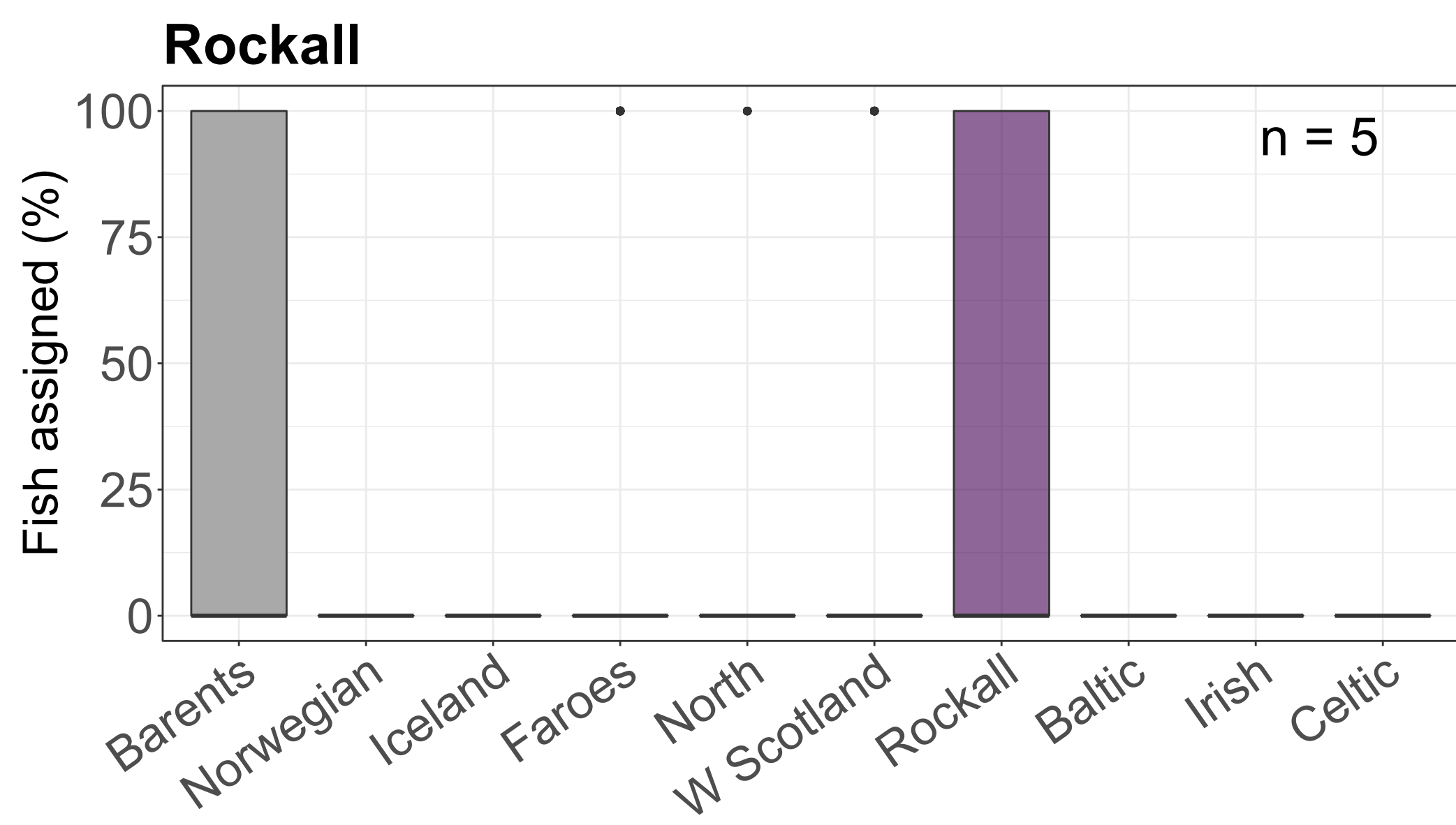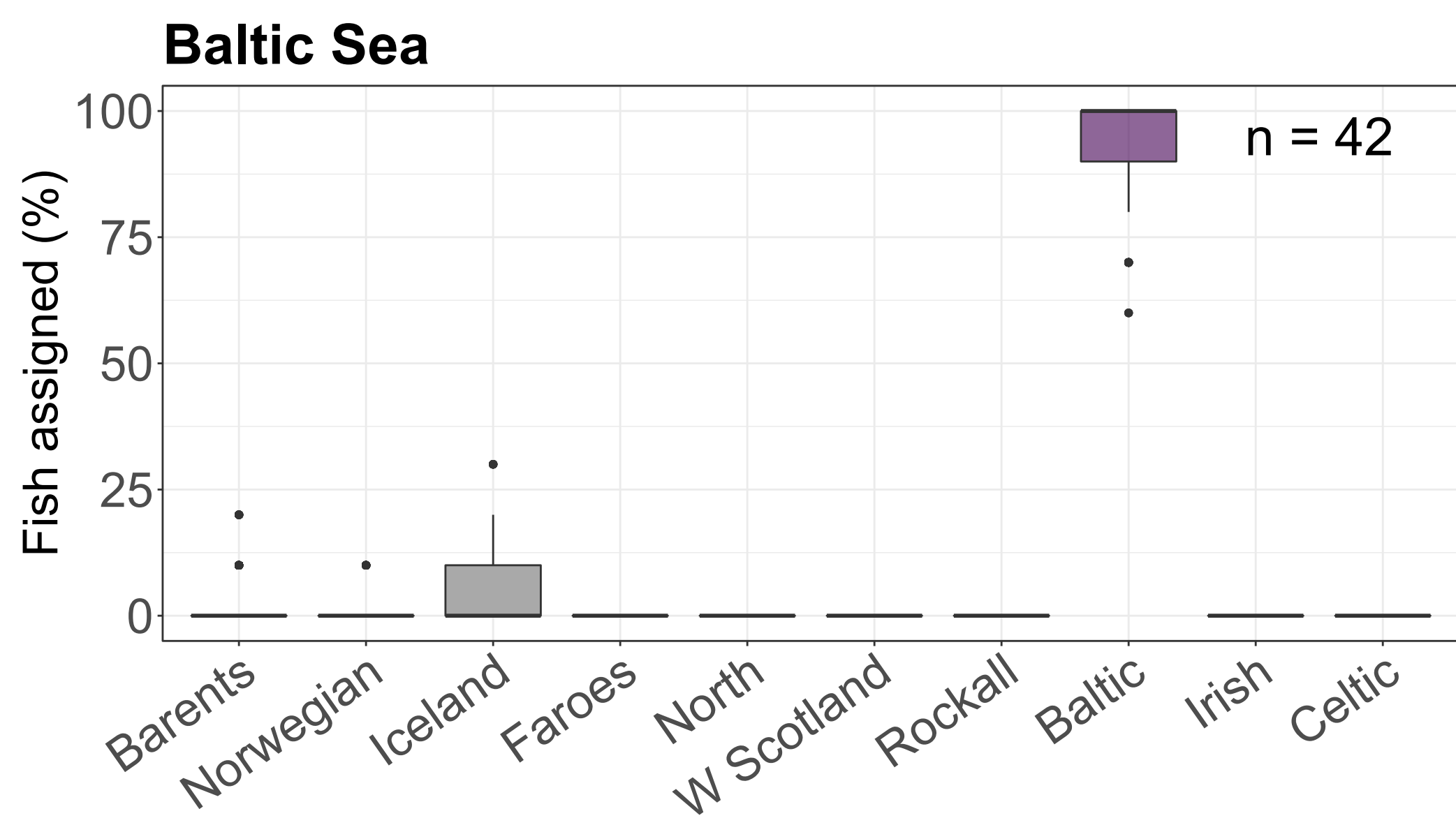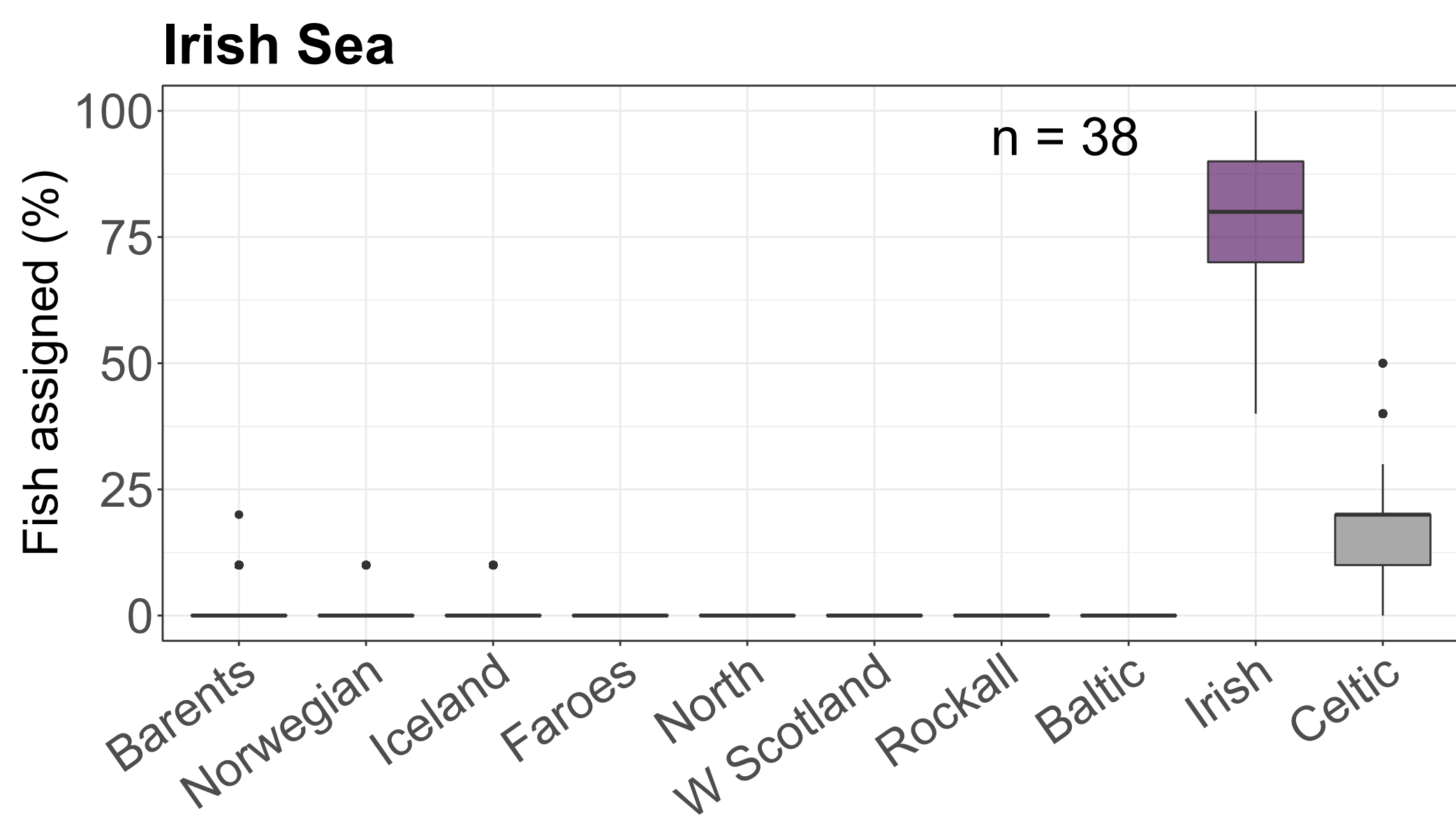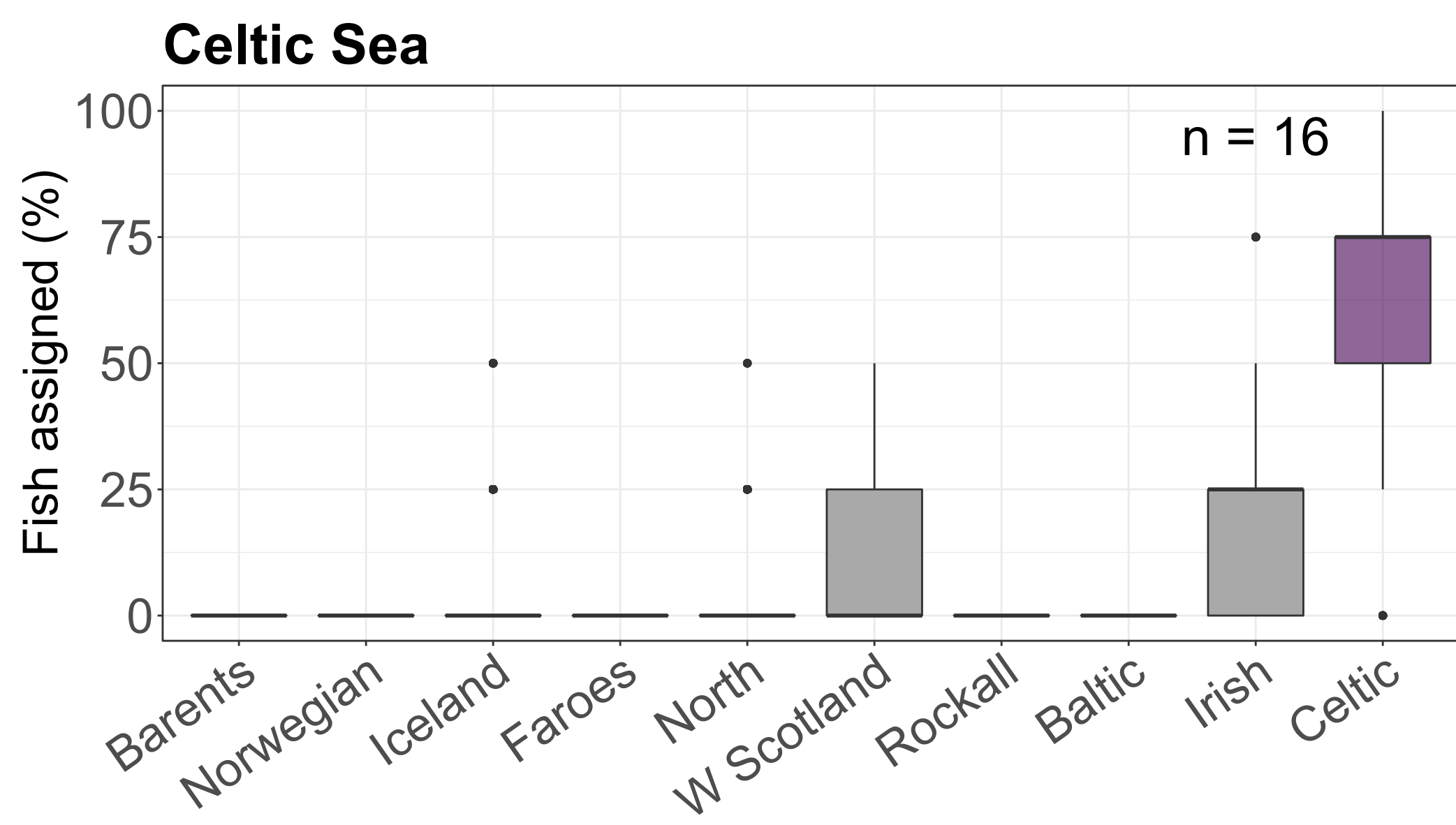

Supplement: Supplementary file 4 — Figure S3 Assignment results using carbon, nitrogen and sulfur stable isotope data, showing the percentage of individuals from each known location assigned to all the possible regions over 1000 repeat simulations. The coloured boxes show the correct regions of origin. [file RCM-39-e9861-s011.pdf]

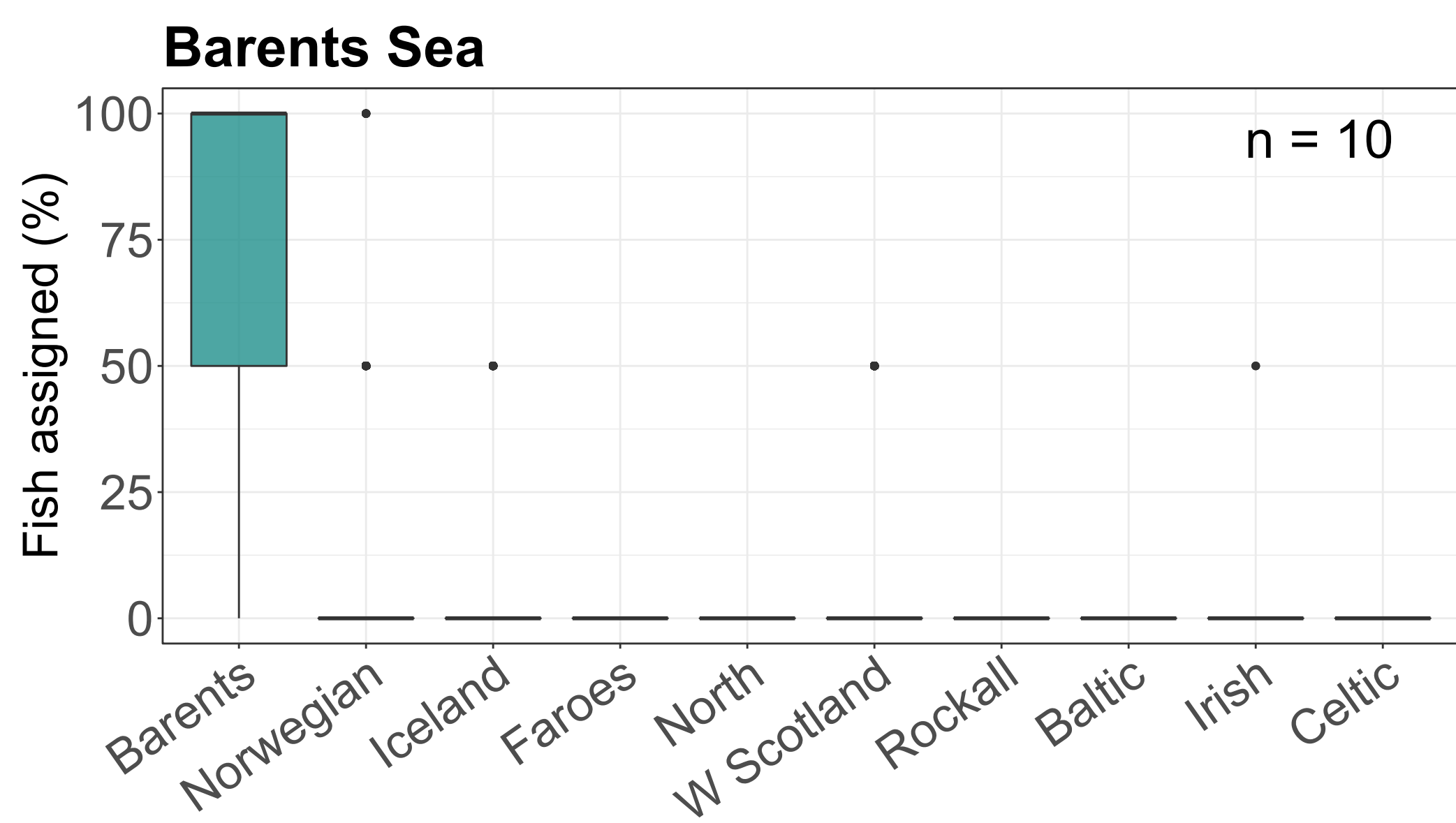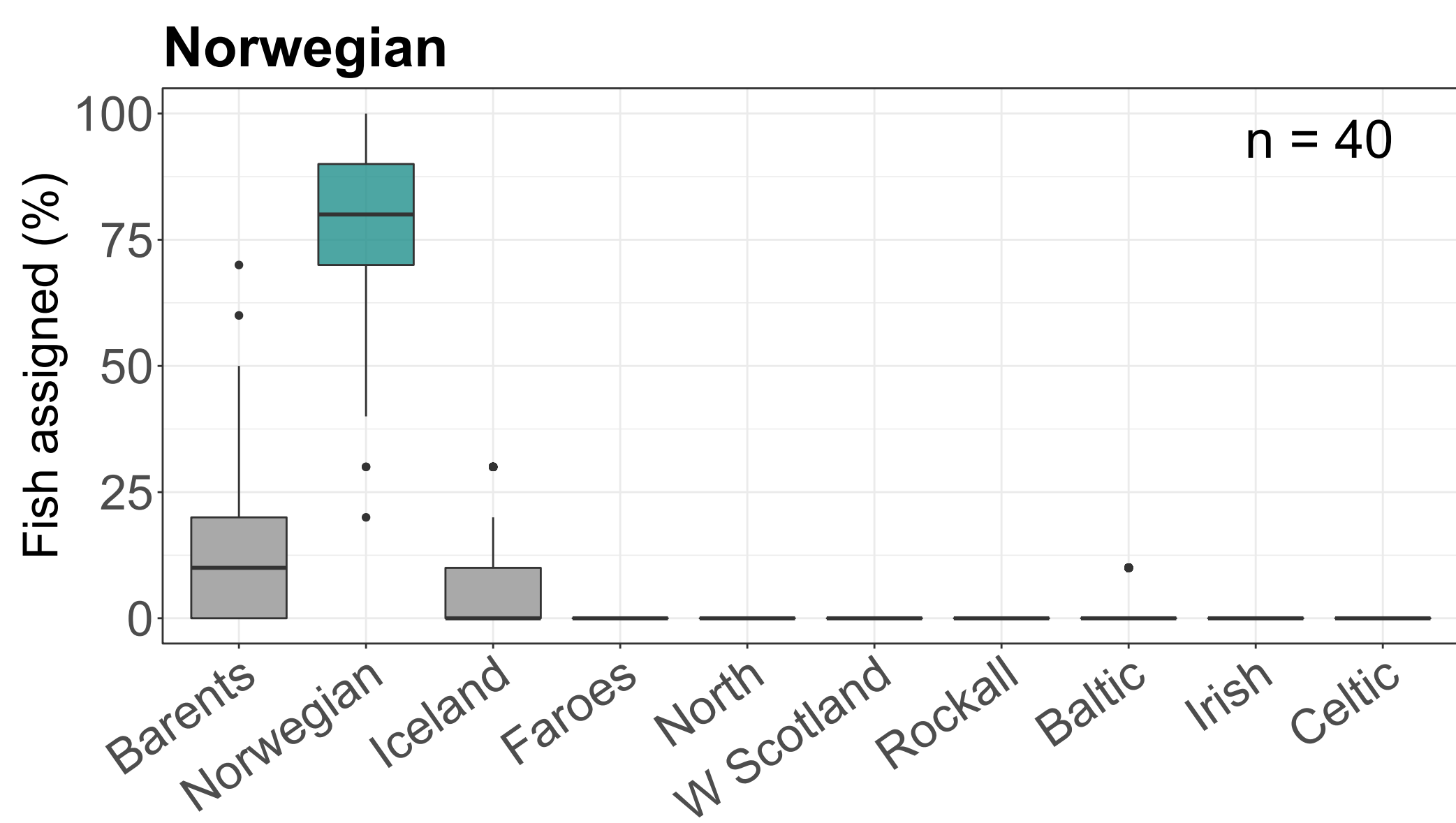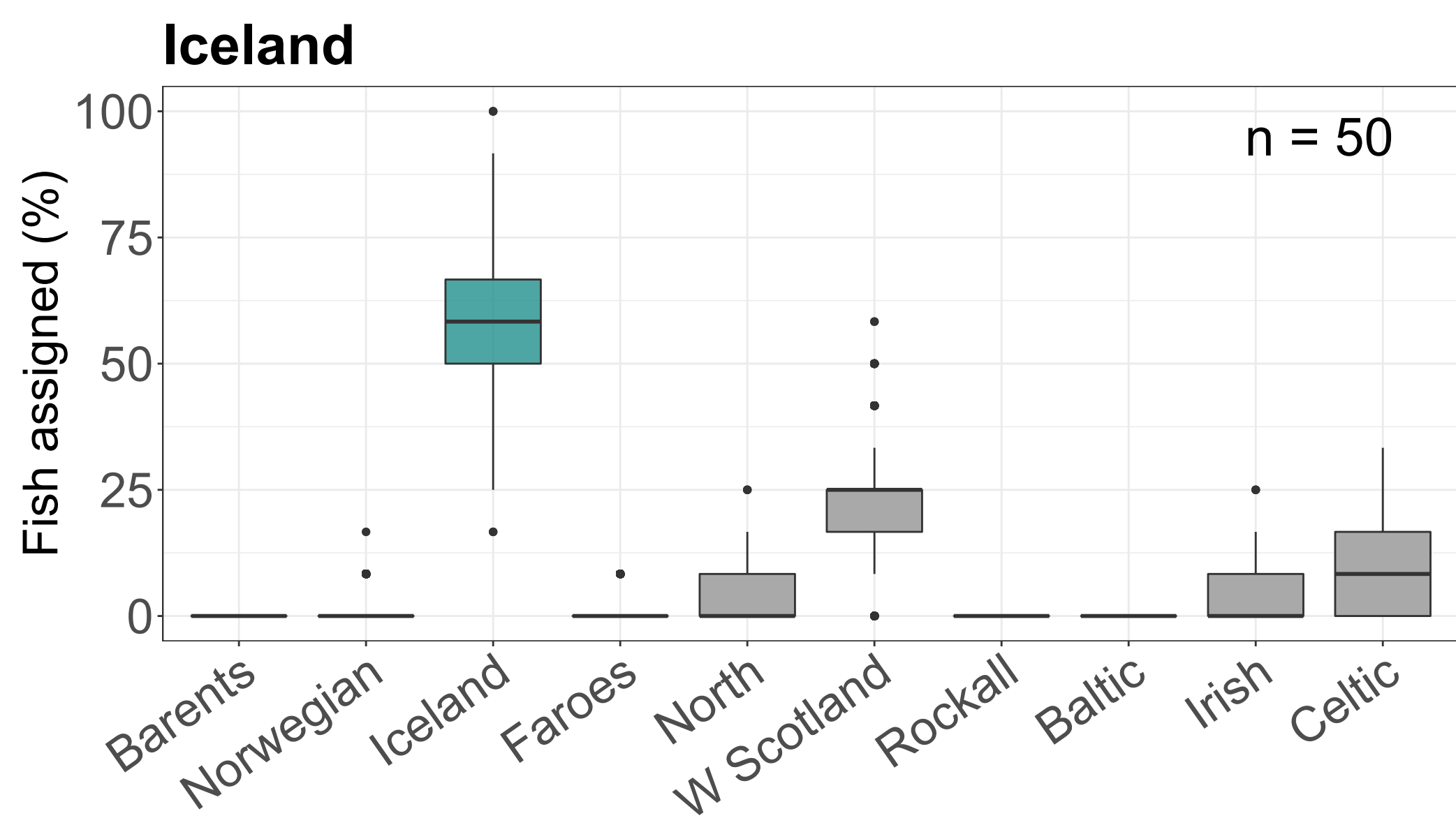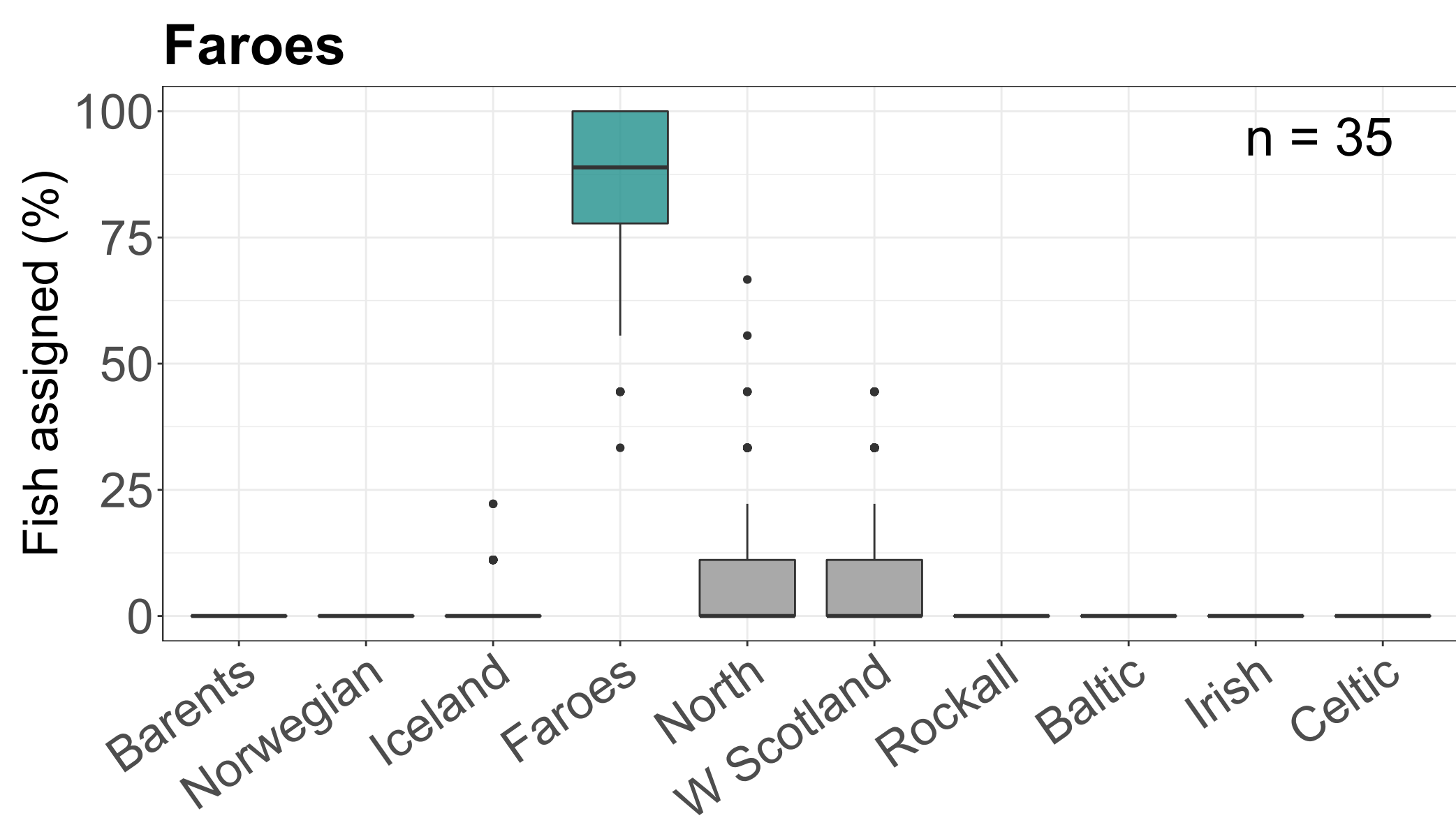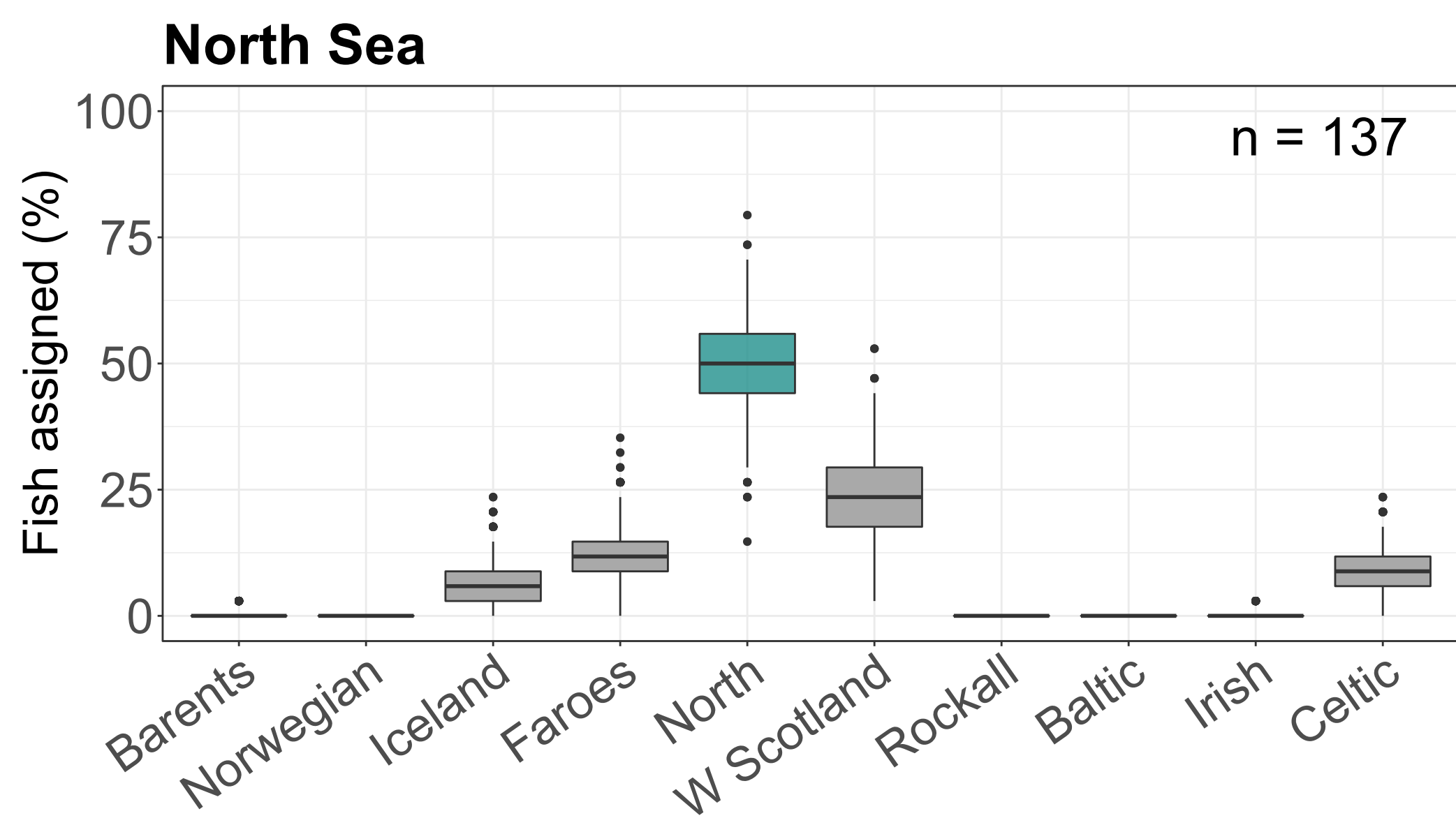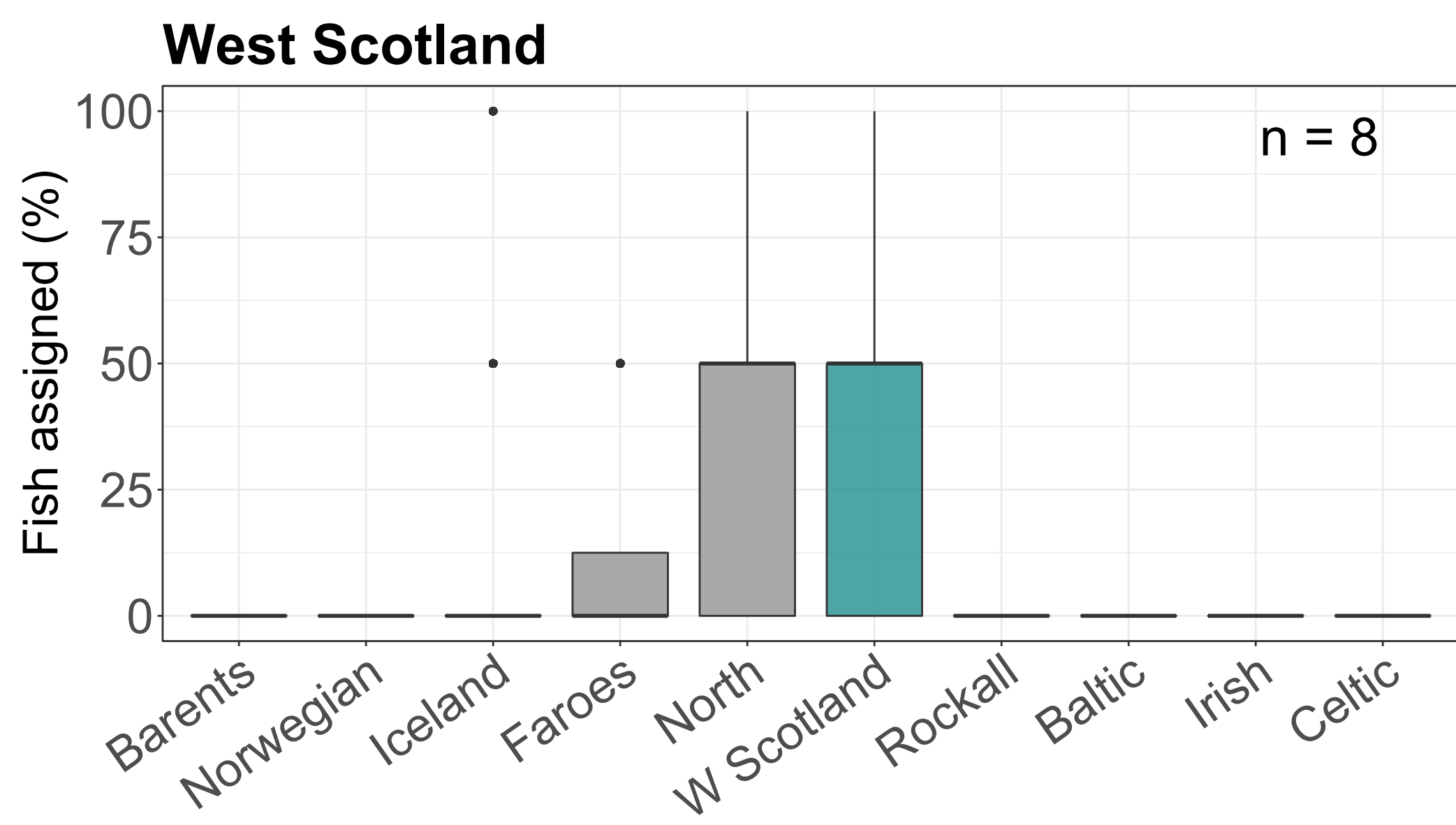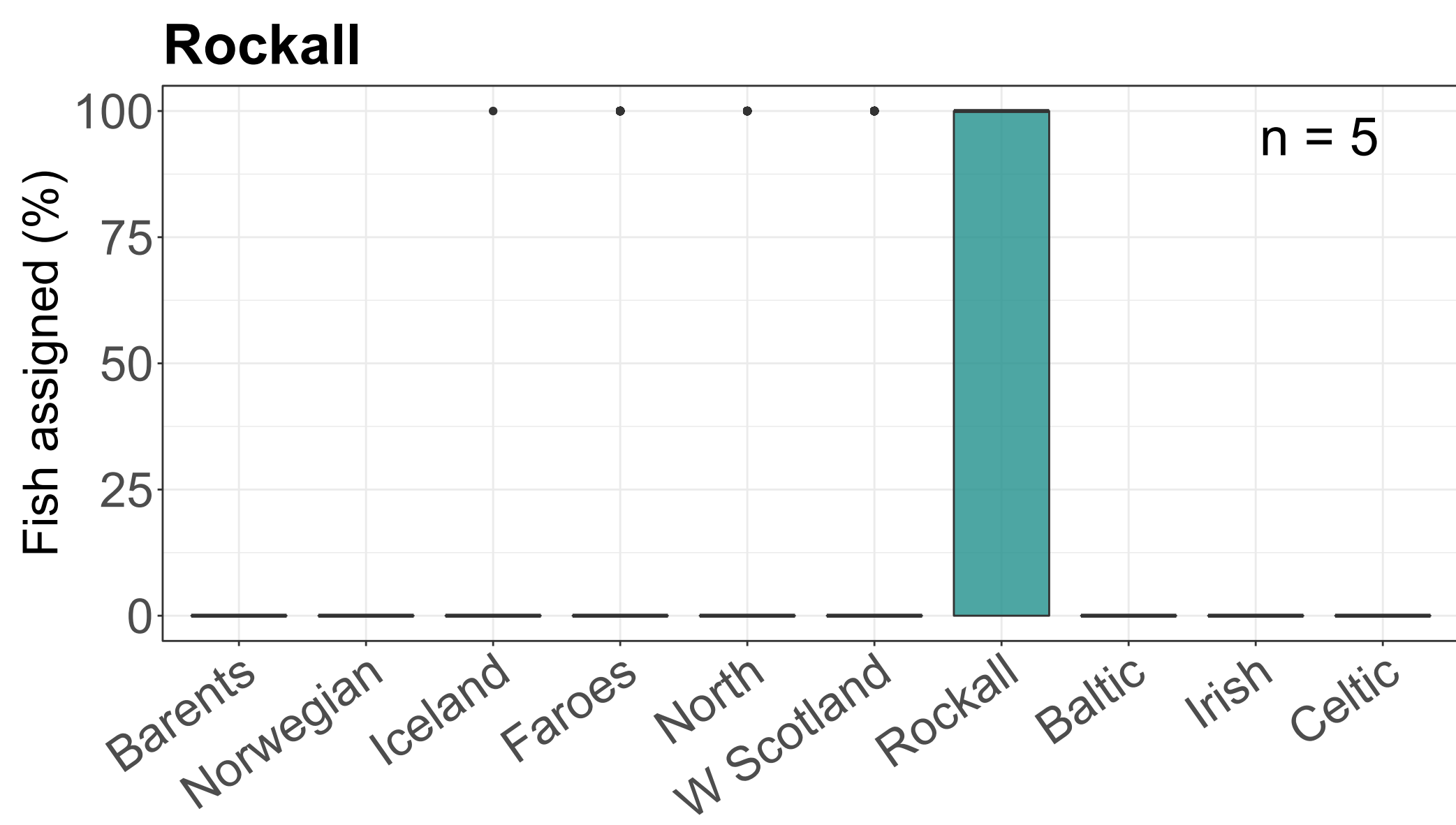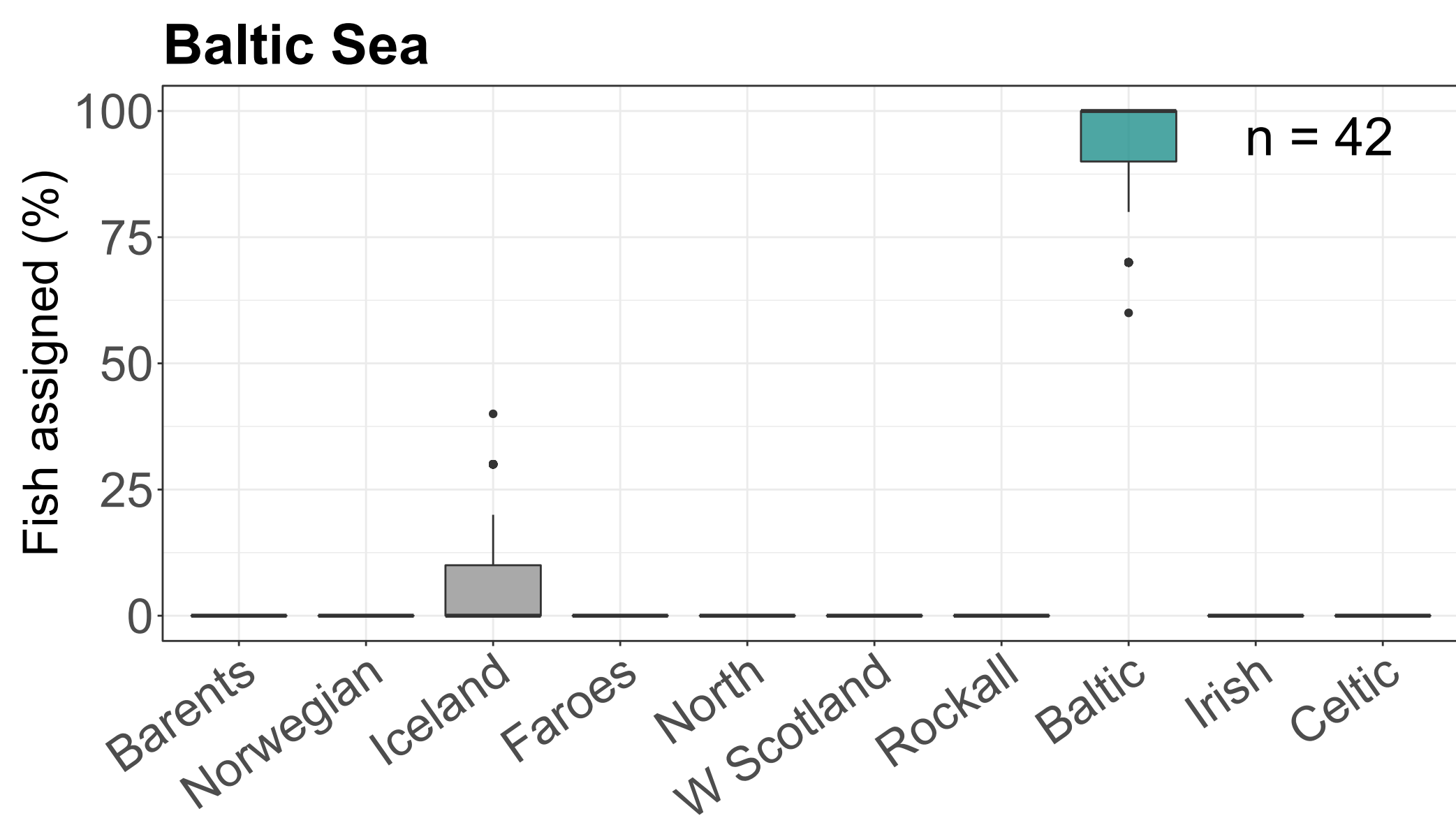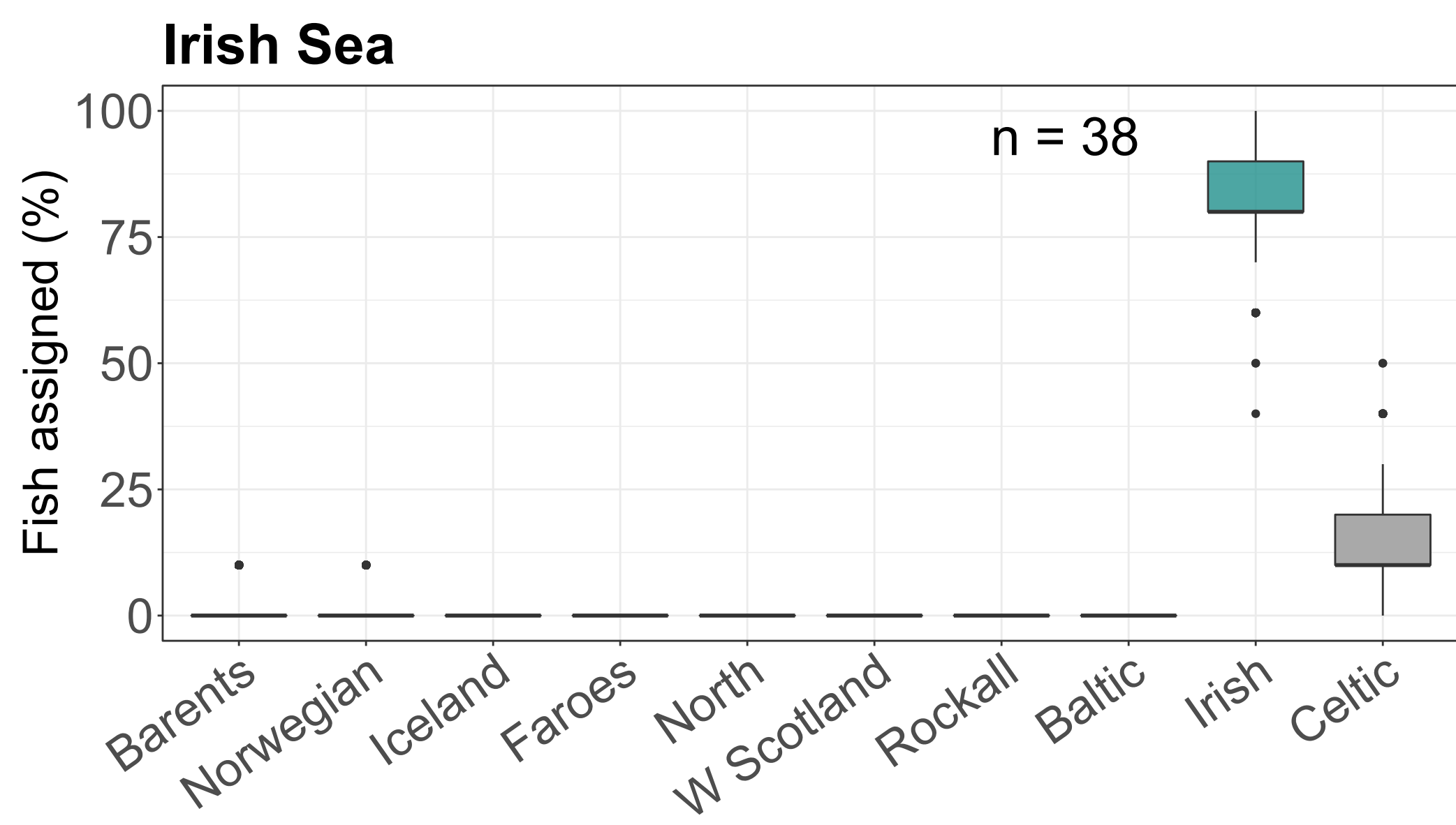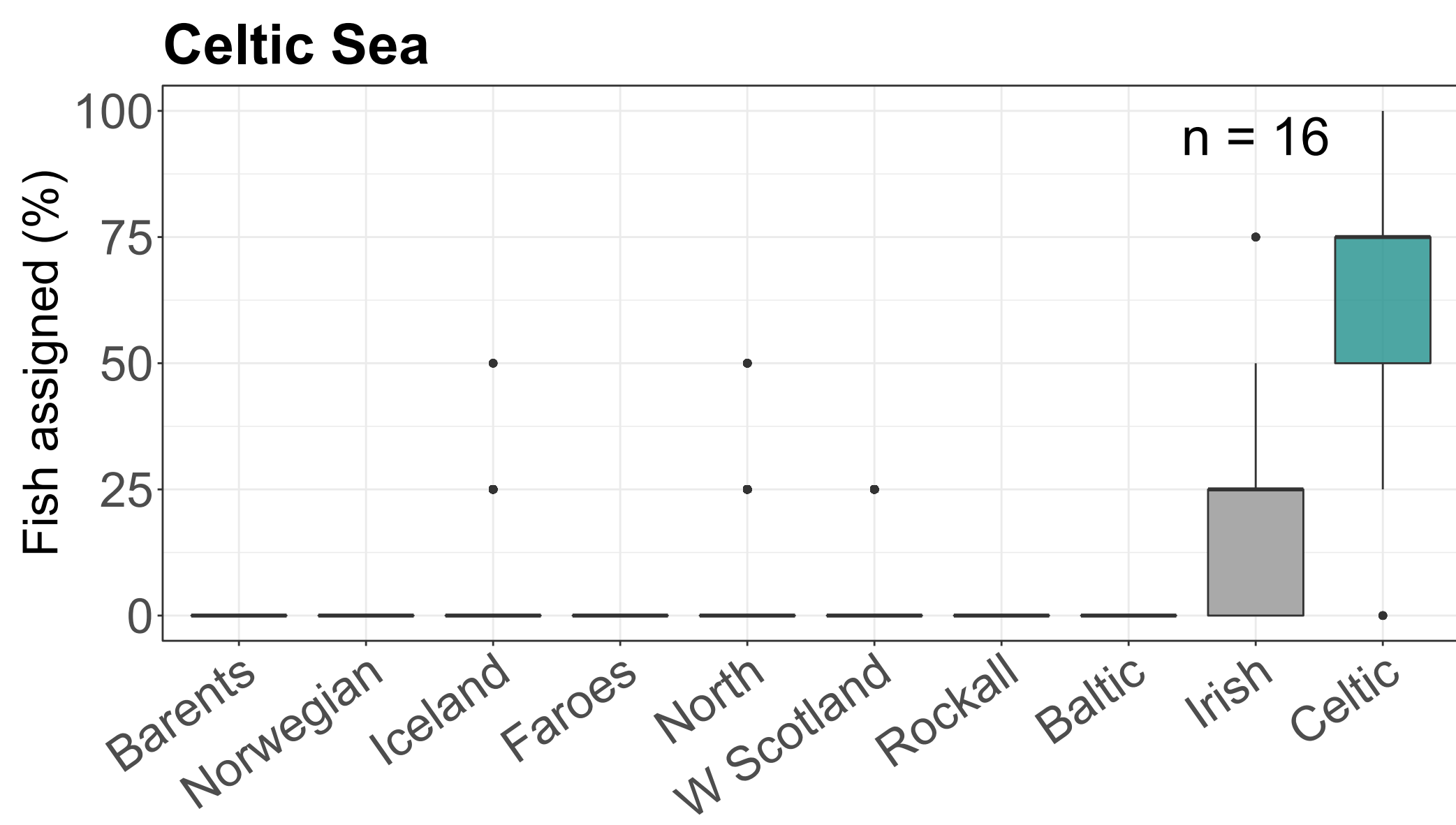

Supplement: Supplementary file 5 — Figure S4 Assignment results using only carbon and nitrogen stable isotope data, showing the percentage of individuals from each known location assigned to all the possible regions over 1000 repeat simulations. The coloured boxes show the correct regions of origin. [file RCM-39-e9861-s012.pdf]

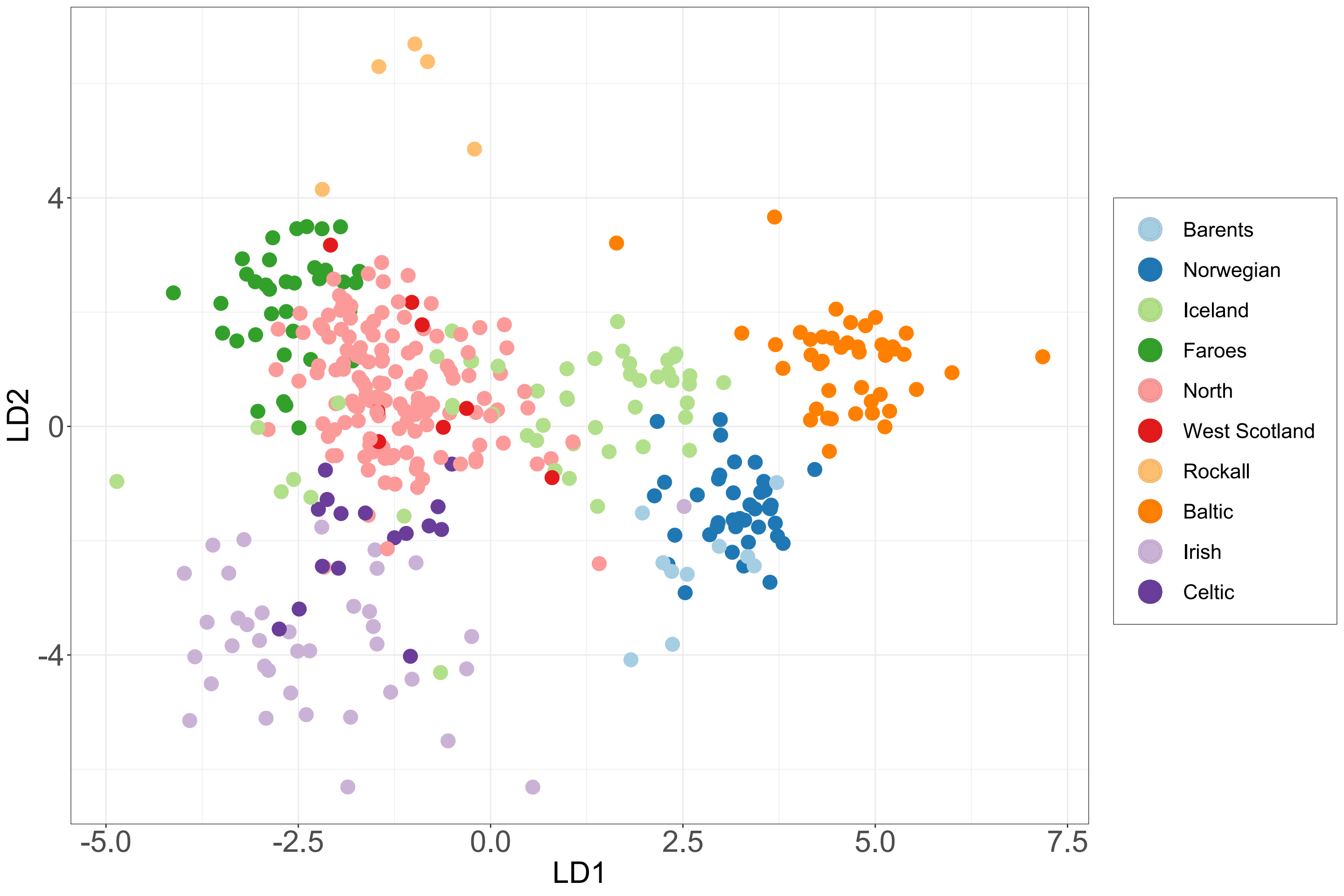

Supplement: Supplementary file 6 — Figure S5 Linear discriminant analysis (LD1 and LD2) using the carbon, nitrogen and sulfur stable isotope compositions measured in cod muscle tissue from each of the sampled regions. [file RCM-39-e9861-s015.pdf]

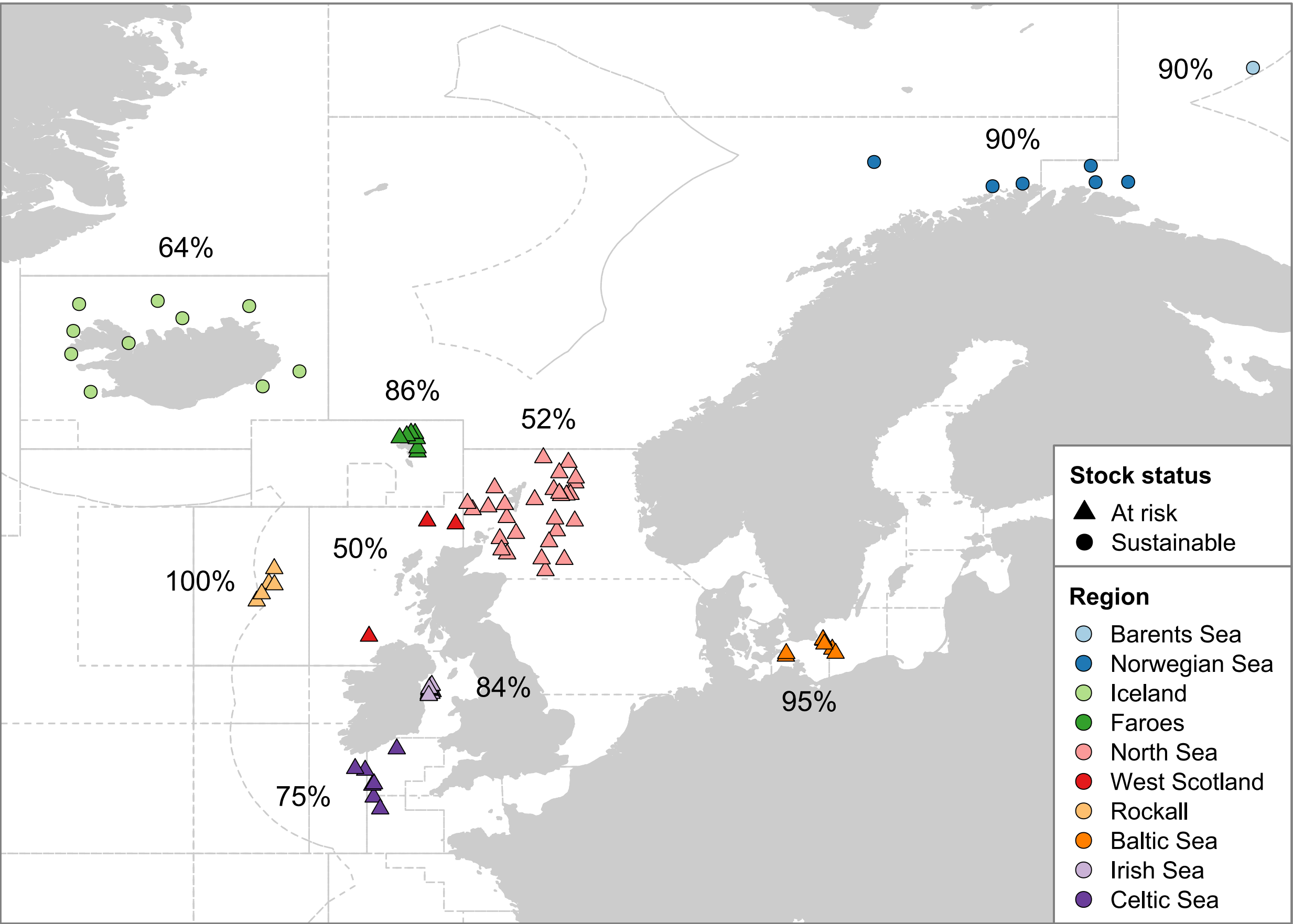

Supplement: Supplementary file 7 — Figure S6 Locations of sampling stations within ICES sub area boundaries (grey lines), and assignment success rates in each region using random forest classification with a leave‐one‐out cross validation approach. [file RCM-39-e9861-s002.pdf]

**Barents**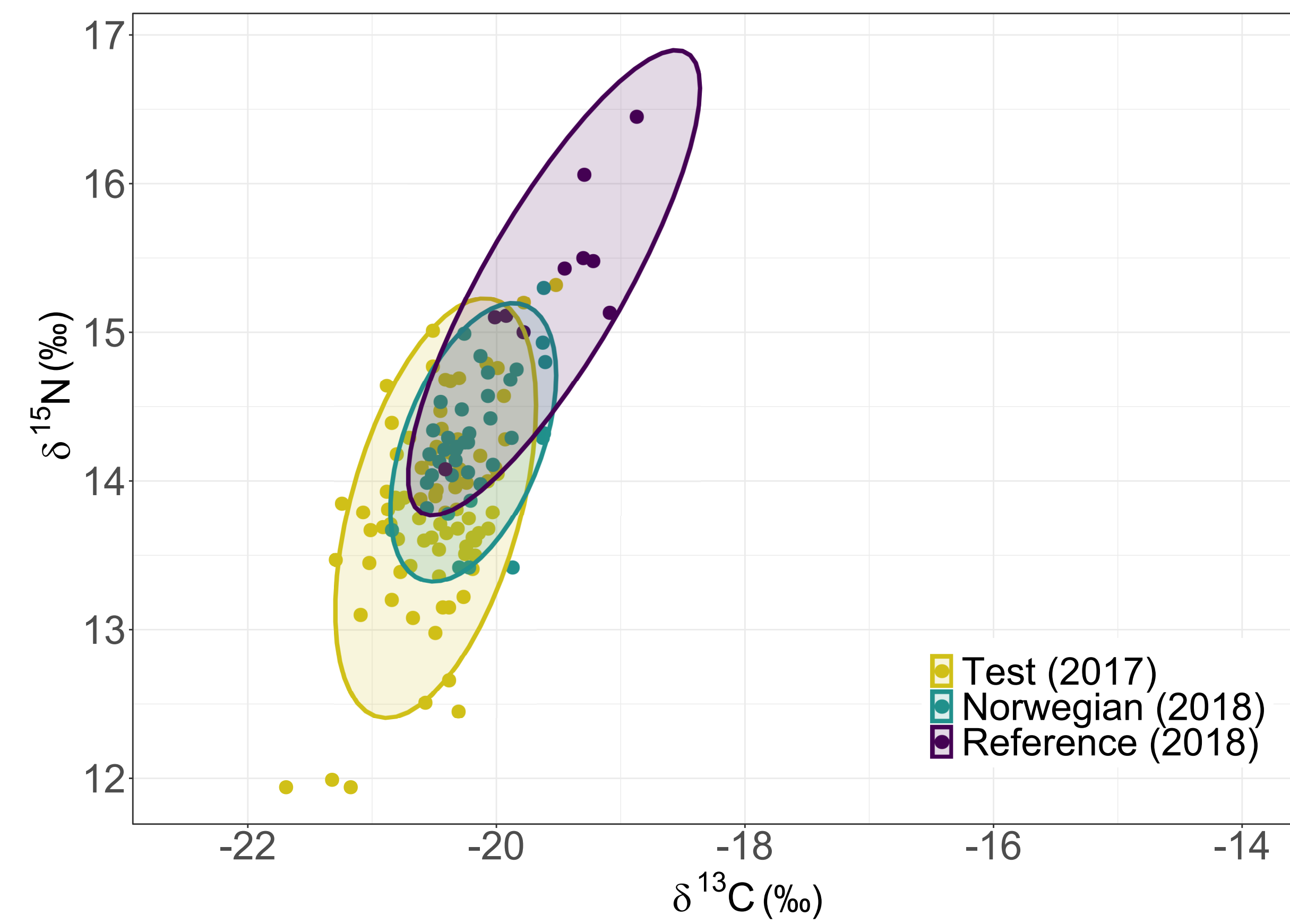**Iceland**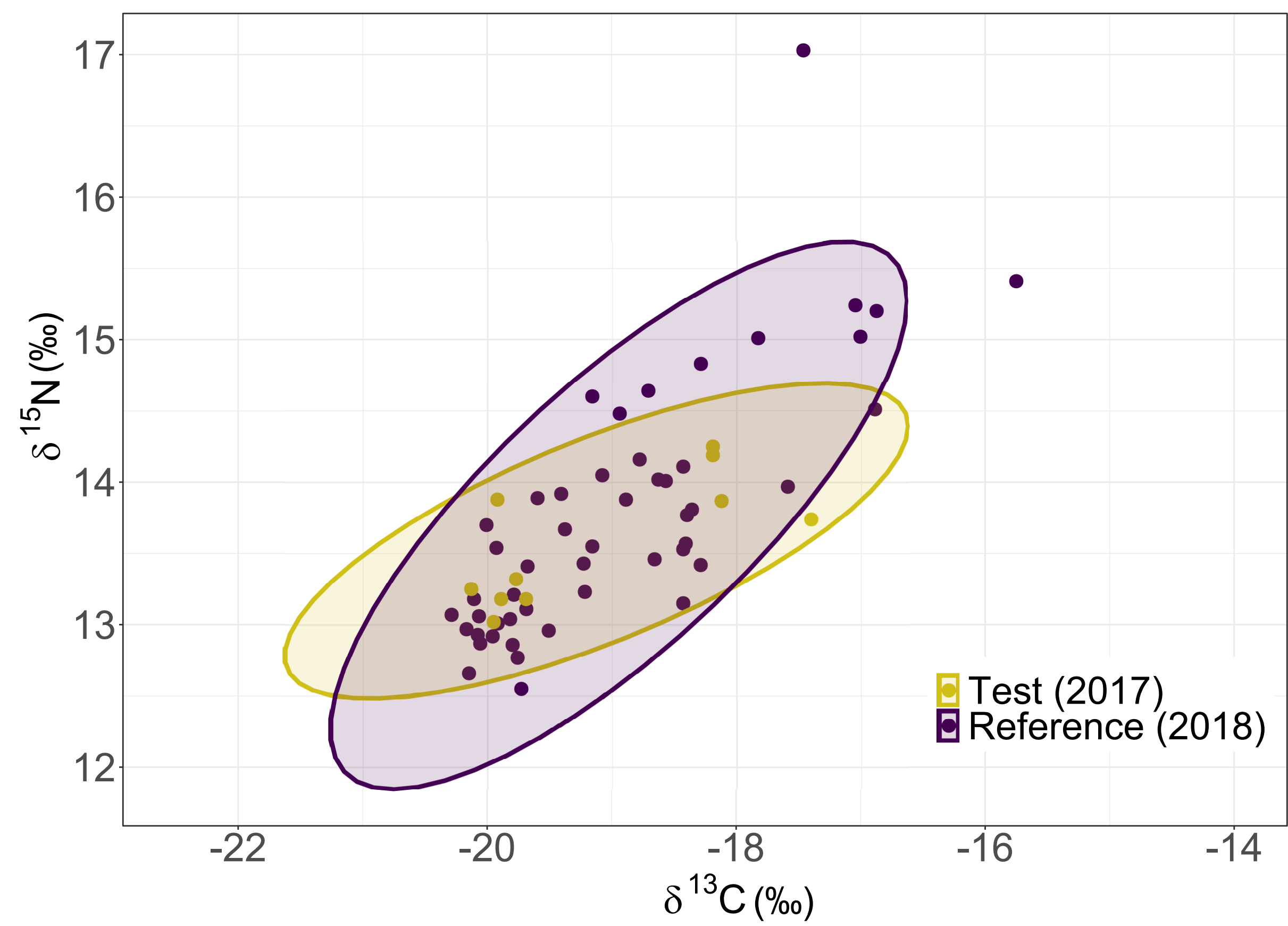**North**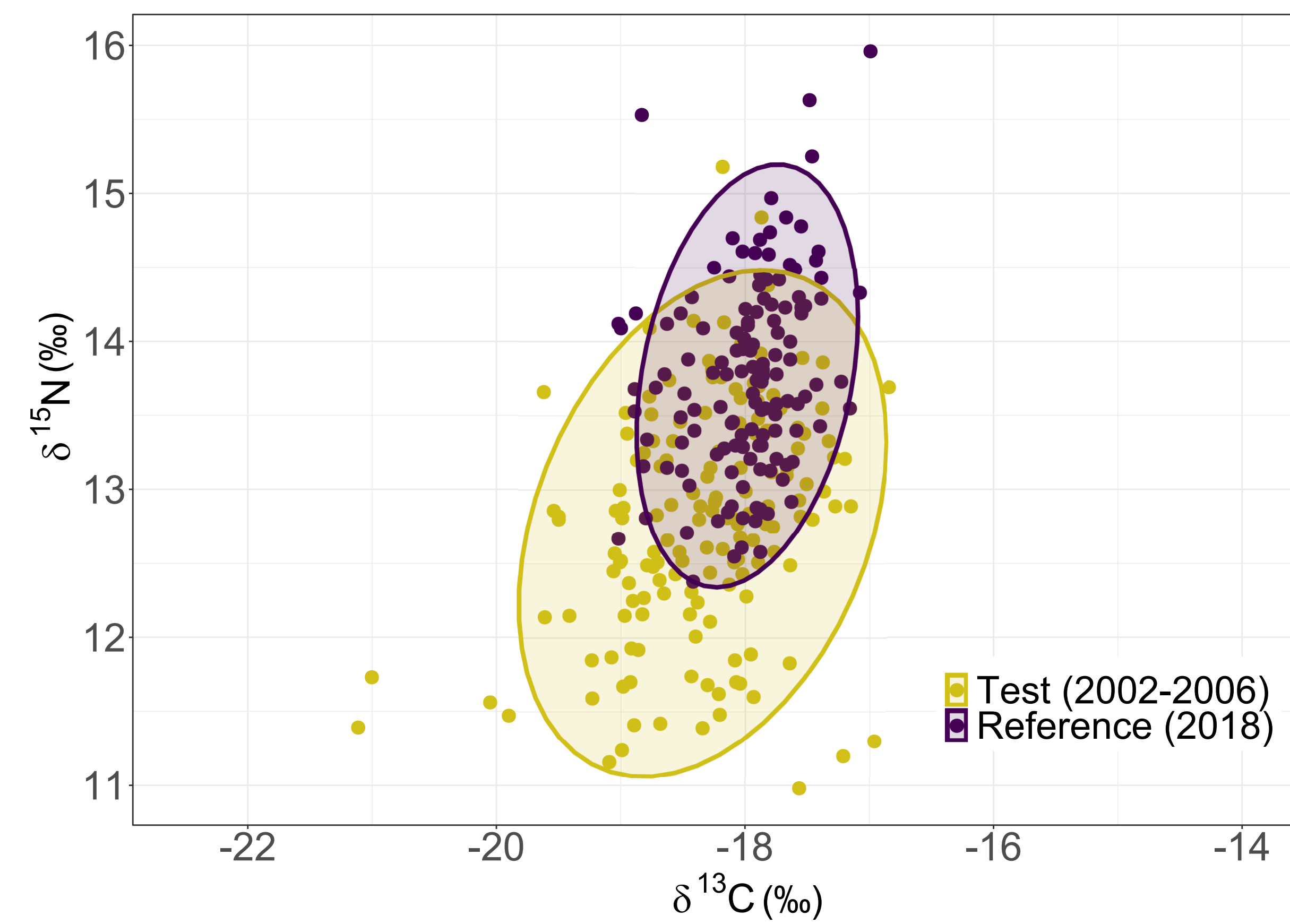**Irish**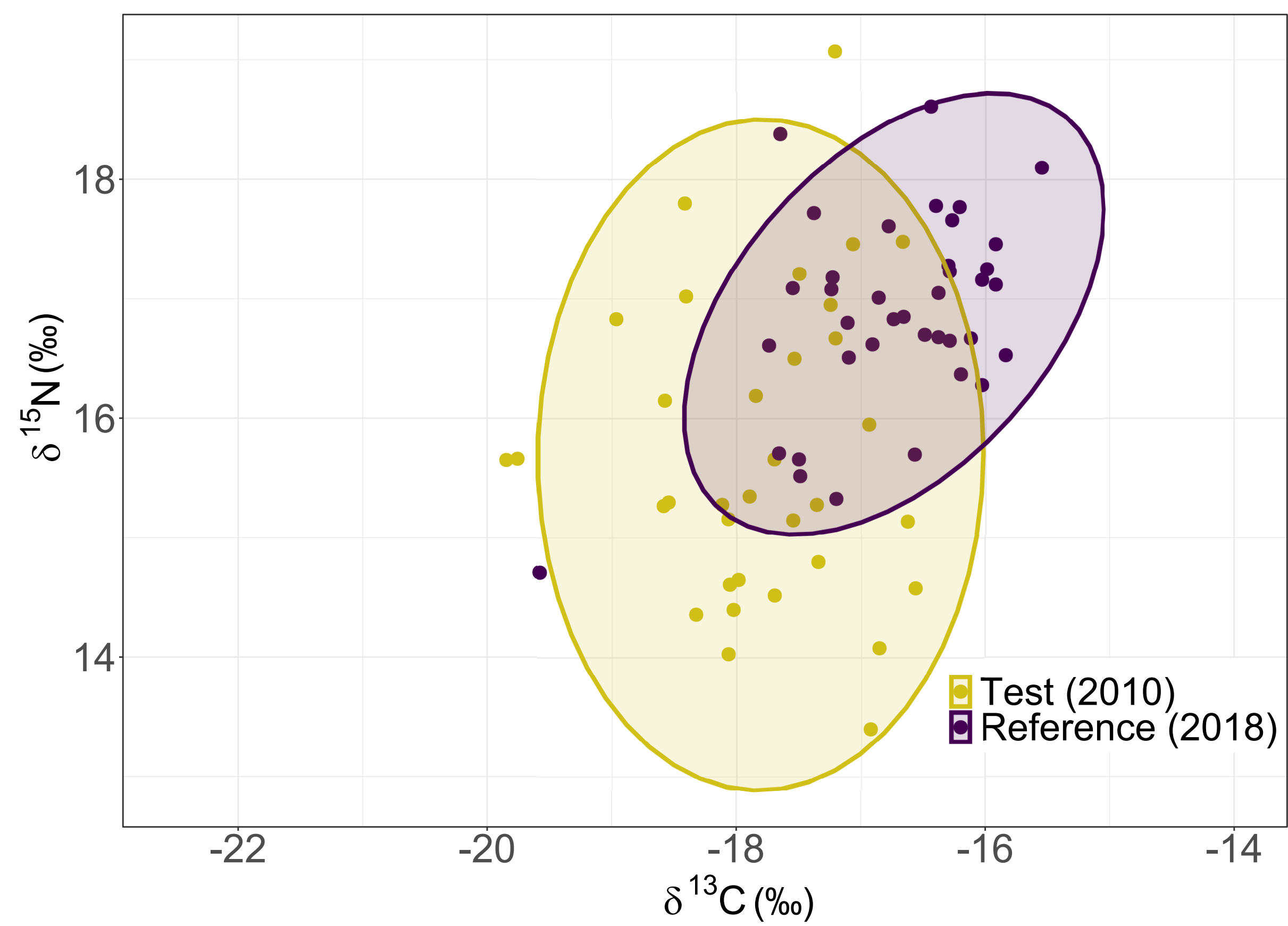**Celtic**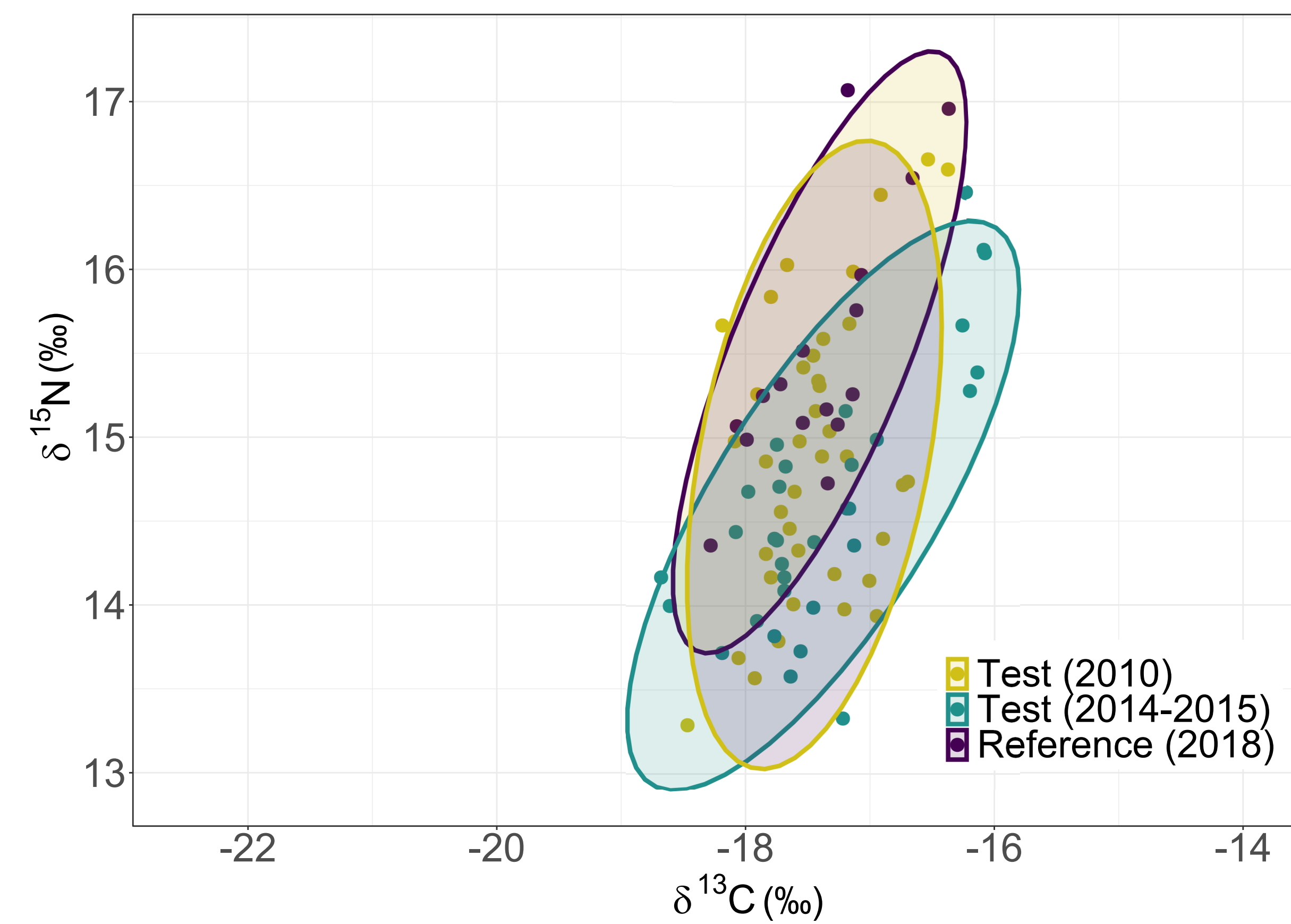

Supplement: Supplementary file 8 — Figure S7 Carbon and nitrogen stable isotope values measured in cod samples collected in this study (reference samples) compared with those collected from previous years and studies (test samples), after applying Suess correction on δ13C values. Previous data: Barents 2017 – collected by Institute of Marine Research (Norway); Iceland 2017 – provided by Young's Seafood Ltd.; North Sea 2002‐2006 – from Jennings and Cogan54; Irish Sea 2010 – from Jennings and Cogan54; Celtic Sea 2010 – from Jennings and Cogan54, Celtic Sea 2014‐2015 – collected by Ifremer55 from the EVHOE 2014 survey. [file RCM-39-e9861-s005.pdf]

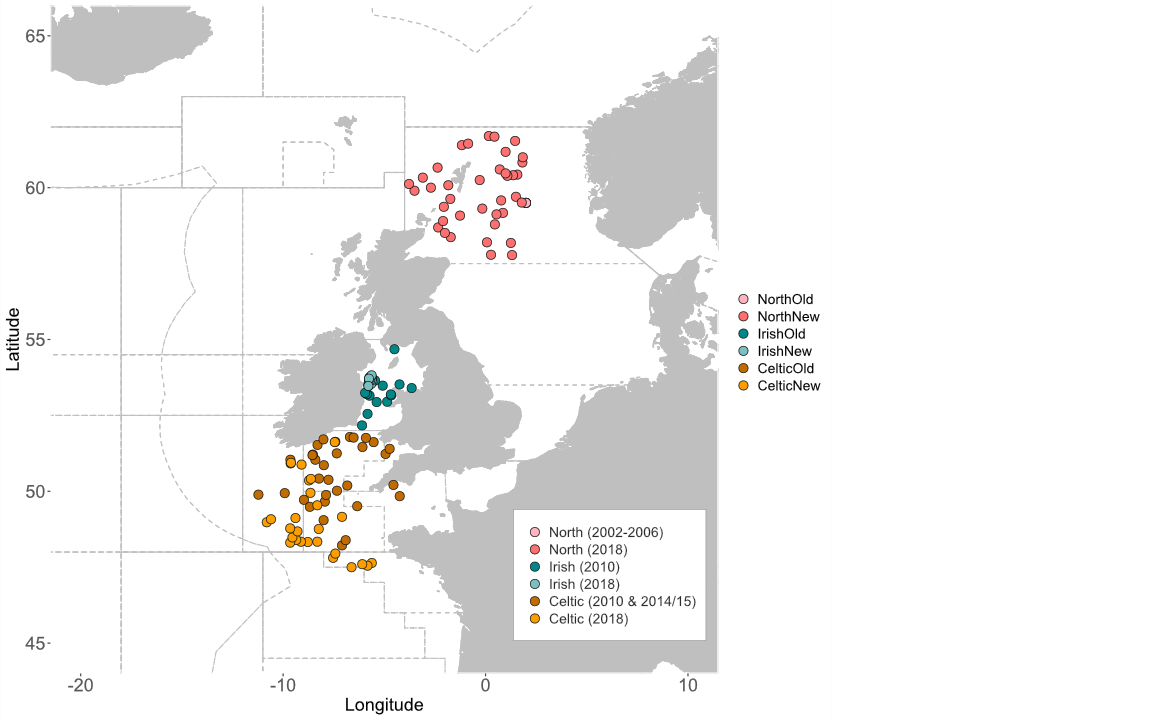

Supplement: Supplementary file 9 — Figure S8 Locations where independent cod samples were collected previously by Jennings and Cogan54 compared to the locations where samples were collected for the current study in 2018 in the corresponding regions. [file RCM-39-e9861-s010.png]
