# Supplementary material for: Tracing the geographic origin of Atlantic cod products using stable isotope analysis
Source: Rapid Commun Mass Spectrom. 2024 Jul 22;39(Suppl 1):e9861. doi: 10.1002/rcm.9861 (PMC12062778; doi:10.1002/rcm.9861)
Supplement: Supplementary file 10 — Data S2. Supporting Information [file RCM-39-e9861-s007.docx]

**SUPPLEMENTARY MATERIAL**

**EXPERIMENTAL**

**Cod muscle samples**

Samples of Atlantic cod muscle tissue were collected from nine different regions in the northeast Atlantic Ocean, and the sample set was extended with additional samples provided by Young’s Seafood Ltd. from the Barents Sea. Table S1 lists the number of samples collected from each region as well as the number of stations sampled, the year samples were collected and the organisations who provided the samples.

**Table S1** Total number of individual Atlantic cod collected from each geographic region, the number of stations sampled per region, and the source from which samples were obtained.

| Catch region | Number of cod sampled | Number of stations | Year samples collected | Source of samples and organisation name |
| --- | --- | --- | --- | --- |
| Barents Sea | 10 | Unknown | 2017 | Young’s Seafood Ltd. |
| Norwegian Sea | 40 | 6 | 2018 | Annual fisheries survey - Institute of Marine Research |
| Iceland | 50 | 10 | 2018 | Annual fisheries survey - Marine and Freshwater Research Institute |
| Faroe Islands | 35 | 7 | 2018 | Annual fisheries survey - Faroe Marine Research Institute |
| North Sea | 133 | 28 | 2018 | Annual fisheries survey - Marine Scotland |
| West Scotland | 8 | 3 | 2018 | Annual fisheries survey - Marine Scotland |
| Rockall | 5 | 5 | 2018 | Annual fisheries survey - Marine Scotland |
| Baltic Sea | 42 | 7 | 2018 | Fish tagging survey - Technical University of Denmark |
| Irish Sea | 38 | 16 | 2018 | Fish tagging survey - Marine Institute |
| Celtic Sea | 16 | 7 | 2018 | Annual fisheries survey - Ifremer |
| Total | **377** | **89** |  |  |

**Statistical analysis**

Multivariate assignment methods were tested using carbon, nitrogen and sulfur isotope markers and then using only carbon and nitrogen stable isotope data, to allow the importance of sulfur isotopes for spatial traceability to be assessed. The bivariate normal probability distributions for each location were fitted using the carbon and nitrogen isotope data, and then a subset of test samples was compared to these. Again, the ‘dmvnorm’ function within the R package ‘mvtnorm’^50^ was used for this, calculating the probability density function of the bivariate normal distribution and therefore the likelihoods of the samples having originated from each location. The location with the greatest likelihood was deemed the most likely origin for each sample. A jack-knifing approach was employed, where 1000 repeat simulations were carried out using different random subsets of test and training data.

**RESULTS**

To determine the measurement error associated with the stable isotope analysis of fish muscle samples, replicate measurements of two internal standards – a fish muscle standard and a glutamic acid standard – were taken. The mean values and standard deviations of these replicates are shown in Table S2.

Table S3 lists the means and standard deviations of carbon, nitrogen and sulfur stable isotope ratios measured in Atlantic cod caught in each of the sampled geographic regions.

**Table S2** Comparison of the mean values and uncertainties (standard deviations) in the stable isotope measurements of two internal standards measured at both laboratories where samples were analysed.

| Standard | Laboratory | δ^13^C (‰) | | δ^15^N (‰) | | δ^34^S (‰) | |
| --- | --- | --- | --- | --- | --- | --- | --- |
|  |  | **Mean** | **SD** | **Mean** | **SD** | **Mean** | **SD** |
| Fish muscle | NOCS | -19.3 | 0.11 | 11.4 | 0.17 | 19.3 | 0.64 |
|  | LSMSF | -19.3 | 0.07 | 11.3 | 0.11 | 18.5 | 0.74 |
| Glutamic acid | NOCS | -13.1 | 0.17 | -3.8 | 0.06 | NA | NA |
|  | LSMSF | -13.6 | 0.43 | -3.9 | 0.15 | NA | NA |

**Table S3** Means and standard deviations of carbon (lipid corrected), nitrogen and sulfur stable isotope ratios from Atlantic cod caught in each of the sampled geographic regions.

| Region | Number of samples | δ^13^C (‰) | | δ^15^N (‰) | | δ^34^S (‰) | |
| --- | --- | --- | --- | --- | --- | --- | --- |
|  |  | **Mean** | **SD** | **Mean** | **SD** | **Mean** | **SD** |
| Barents Sea | 10 | -19.5 | 0.48 | 15.3 | 0.64 | 18.2 | 0.64 |
| Norwegian Sea | 40 | -20.2 | 0.30 | 14.3 | 0.42 | 19.7 | 0.50 |
| Iceland | 50 | -19.0 | 1.05 | 13.8 | 0.87 | 18.7 | 0.92 |
| Faroe Islands | 35 | -17.4 | 0.37 | 13.1 | 0.64 | 18.5 | 0.41 |
| North Sea | 133 | -18.0 | 0.42 | 13.8 | 0.66 | 19.0 | 0.50 |
| West Scotland | 8 | -18.3 | 0.41 | 13.5 | 0.73 | 19.4 | 0.55 |
| Rockall | 5 | -18.8 | 0.43 | 10.5 | 0.63 | 18.7 | 0.63 |
| Baltic Sea | 42 | -21.1 | 0.36 | 12.7 | 0.49 | 18.1 | 1.10 |
| Irish Sea | 38 | -16.7 | 0.76 | 16.9 | 0.83 | 18.2 | 0.83 |
| Celtic Sea | 16 | -17.4 | 0.51 | 15.5 | 0.77 | 18.8 | 0.70 |

Figure S1 shows frequency distributions for the carbon, nitrogen and sulfur stable isotope data for cod from each region, and demonstrates that the majority of regions show a normal distribution.


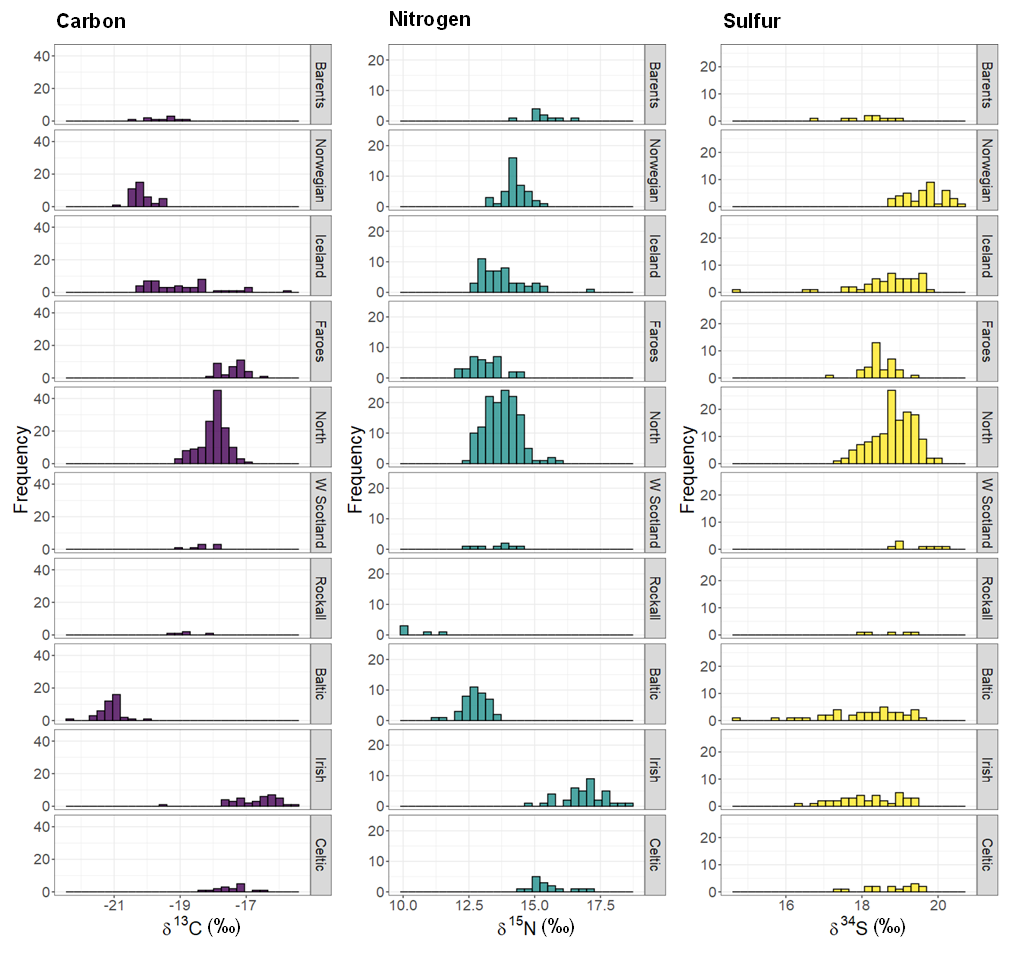


**Figure S1** Frequency distributions of δ^13^C, δ^15^N and δ^34^S values from Atlantic cod caught within each of the ten sampled regions.

### Grouping of stable isotope data by region

Figure S2 shows that the cod samples are grouped into two main clusters based on δ^13^C values – one contains the Baltic, Norwegian and Barents Seas with low δ^13^C values due to low salinity (Baltic) and cold temperatures, and the other contains warmer, more southerly regions around the Faroes and UK shelf seas that yield cod with higher δ^13^C values.


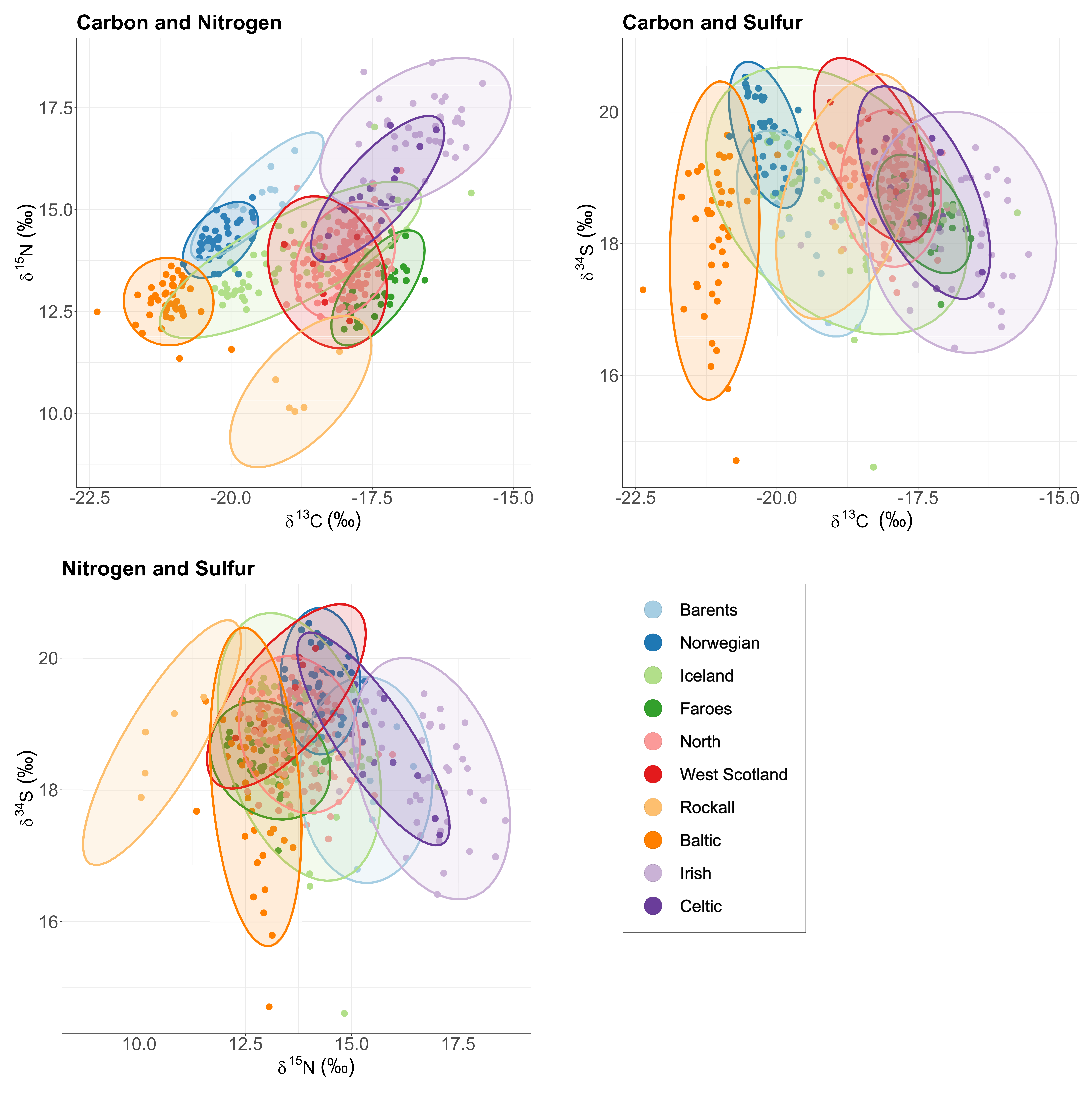


**Figure S2** Carbon, nitrogen and sulfur stable isotope values for each individual cod sampled, coloured by region of origin. The 90% data ellipses are also shown for each geographic region.

**Assignments to catch location**

The results of multivariate assignment using all three isotopes – carbon, nitrogen and sulfur – are displayed in Figure S3, which shows the percentage of individuals from each known location assigned to all the possible regions over 1000 repeat simulations.

The results of multivariate assignment using only carbon and nitrogen isotopes, and not including sulfur isotopes, are shown in Figure S4. This resulted in an average assignment accuracy of 71% among all regions. This is almost exactly the same as the accuracy using all three isotopes, indicating that δ^34^S contributes little to the spatial traceability of cod in the regions studied. For most locations the correct assignments were very similar when using δ^13^C and δ^15^N compared with using all three isotopes (Table S4). There was even lower assignment success with sulfur included in some cases, such as the Celtic Sea and Rockall where more samples were assigned to West Scotland and the Barents Sea respectively (Figure S3). However, fewer West Scotland cod were assigned to Iceland and the Faroes when sulfur was included.

**Figure S3** Assignment results using carbon, nitrogen and sulfur stable isotope data, showing the percentage of individuals from each known location assigned to all the possible regions over 1000 repeat simulations. The coloured boxes show the correct regions of origin.


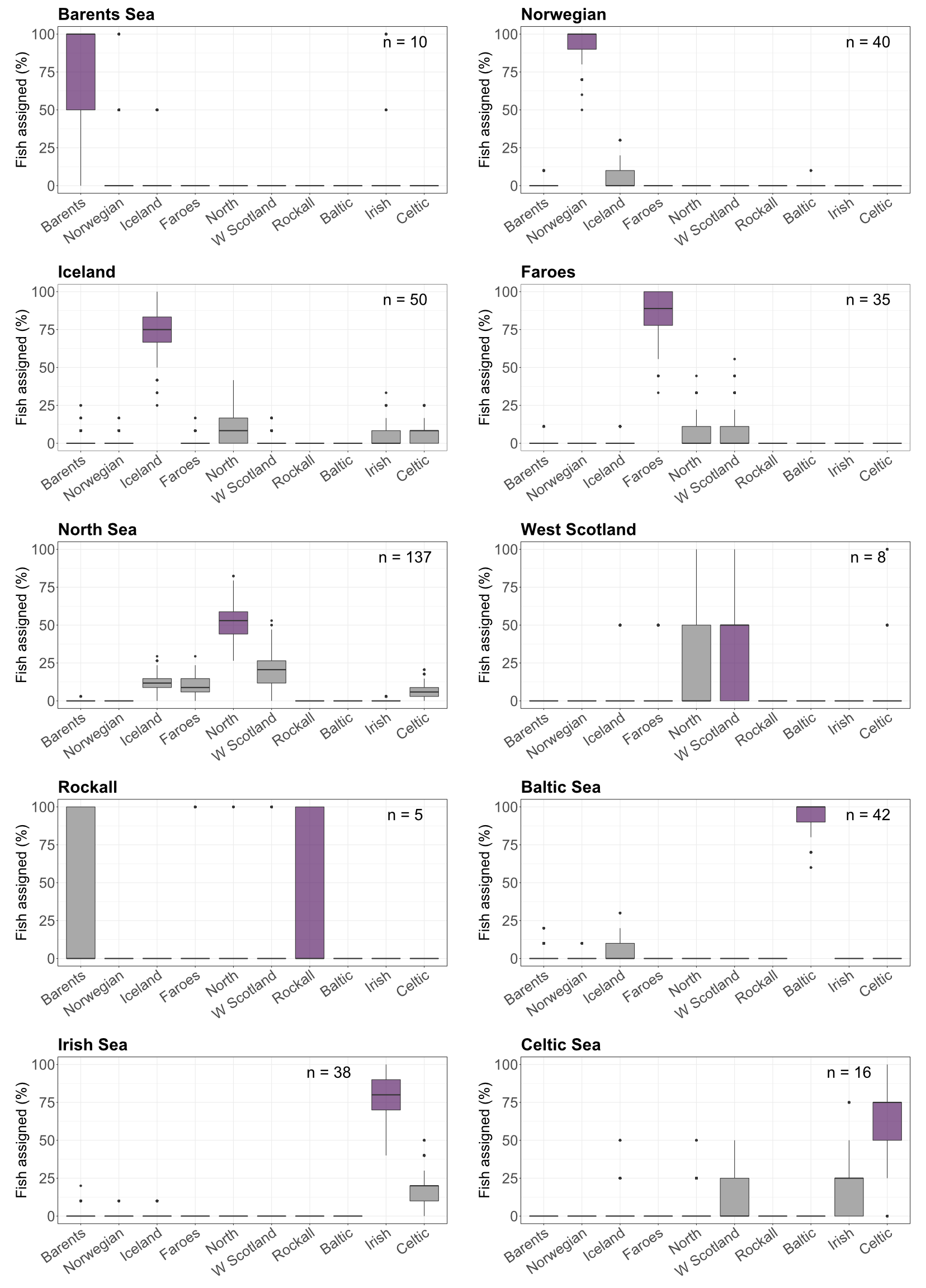


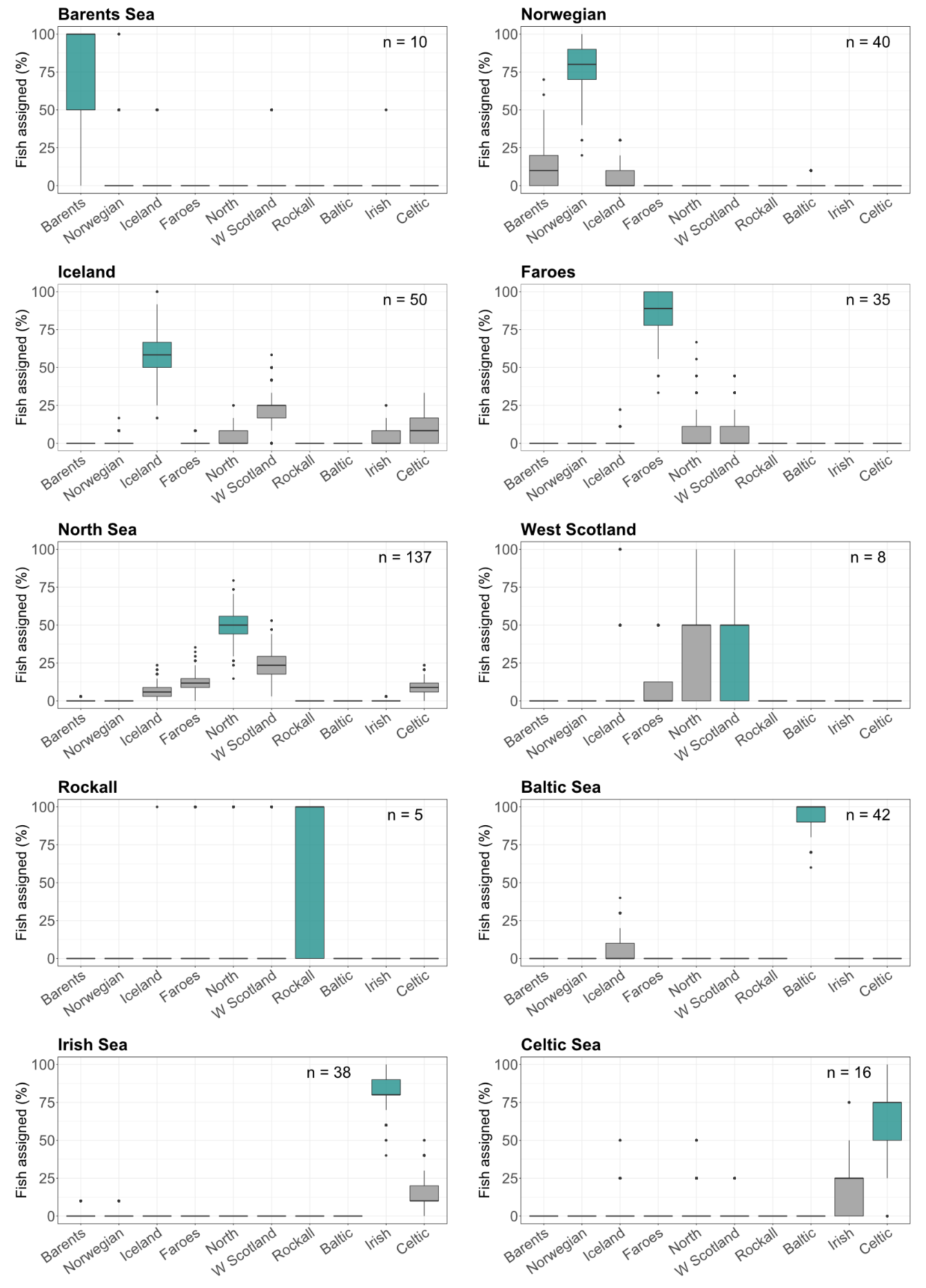


**Figure S4** Assignment results using only carbon and nitrogen stable isotope data, showing the percentage of individuals from each known location assigned to all the possible regions over 1000 repeat simulations. The coloured boxes show the correct regions of origin.

**Table S4** Mean percentage of individuals assigned to the correct origin region over
1000 repeat simulations using three isotopes (δ^13^C, δ^15^N and δ^34^S) and two isotopes
(δ^13^C and δ^15^N).

| Region | Mean correct assignments (%) | |
| --- | --- | --- |
|  | **CNS** | **CN** |
| Barents | 83 | 80 |
| Norwegian | 95 | 82 |
| Iceland | 73 | 59 |
| Faroes | 85 | 87 |
| North Sea | 52 | 50 |
| West Scotland | 44 | 40 |
| Rockall | 40 | 61 |
| Baltic | 94 | 94 |
| Irish | 80 | 84 |
| Celtic | 62 | 70 |
| Total | **71%** | **71%** |

Leave-one-out cross validation using the multivariate normal probability method gave similar results to the stratified jack-knifing multivariate technique using all three stable isotopes, with an overall success rate of 72% (Table S5), since the two methods are very similar. However, cross validation performed better than the original multivariate technique for some regions and slightly worse for others. For the Barents Sea and West Scotland, the assignment accuracy increased to 90% and 50% respectively using cross validation, whereas the Iceland and North Sea assignment accuracies decreased to 70% and 47%. However, the results showed a maximum difference of 7% between the two methods, which gives confidence that the inferences are robust to the multivariate technique used, whether stratified jack-knifing or leave-one-out cross validation.

**Table S5** Leave-one-out cross validation results using the multivariate normal probability method, showing the number of samples assigned to each of the geographic regions as well as the percentage of correct assignments for each region. True known origins are shown in the columns and the assigned most likely origins are shown in the rows.

| Assigned origin | True origin region – number assigned | | | | | | | | | |
| --- | --- | --- | --- | --- | --- | --- | --- | --- | --- | --- |
|  | Barents | Norwegian | Iceland | Faroes | North Sea | West Scotland | Rockall | Baltic | Irish | Celtic |
| Barents | **9** | 0 | 1 | 0 | 0 | 0 | 0 | 0 | 1 | 0 |
| Norwegian | 1 | **39** | 1 | 0 | 0 | 0 | 0 | 0 | 0 | 0 |
| Iceland | 0 | 1 | **35** | 1 | 15 | 1 | 0 | 1 | 0 | 1 |
| Faroes | 0 | 0 | 1 | **30** | 14 | 1 | 3 | 0 | 0 | 0 |
| North Sea | 0 | 0 | 4 | 2 | **64** | 2 | 0 | 0 | 0 | 0 |
| West Scotland | 0 | 0 | 2 | 2 | 33 | **4** | 0 | 0 | 0 | 1 |
| Rockall | 0 | 0 | 0 | 0 | 0 | 0 | **2** | 0 | 0 | 0 |
| Baltic | 0 | 0 | 0 | 0 | 0 | 0 | 0 | **41** | 0 | 0 |
| Irish | 0 | 0 | 2 | 0 | 1 | 0 | 0 | 0 | **31** | 4 |
| Celtic | 0 | 0 | 4 | 0 | 10 | 0 | 0 | 0 | 6 | **10** |
| Percentage correct | **90%** | **98%** | **70%** | **86%** | **47%** | **50%** | **40%** | **98%** | **82%** | **63%** |

Linear discriminant analysis resulted in clear clustering of the stable isotope data by catch location, with relatively little overlap for many regions (Figure S5). Certain regions do show overlap with other clusters, in general with the regions that are closest geographically, such as the Barents and Norwegian Seas or West Scotland and the North Sea.


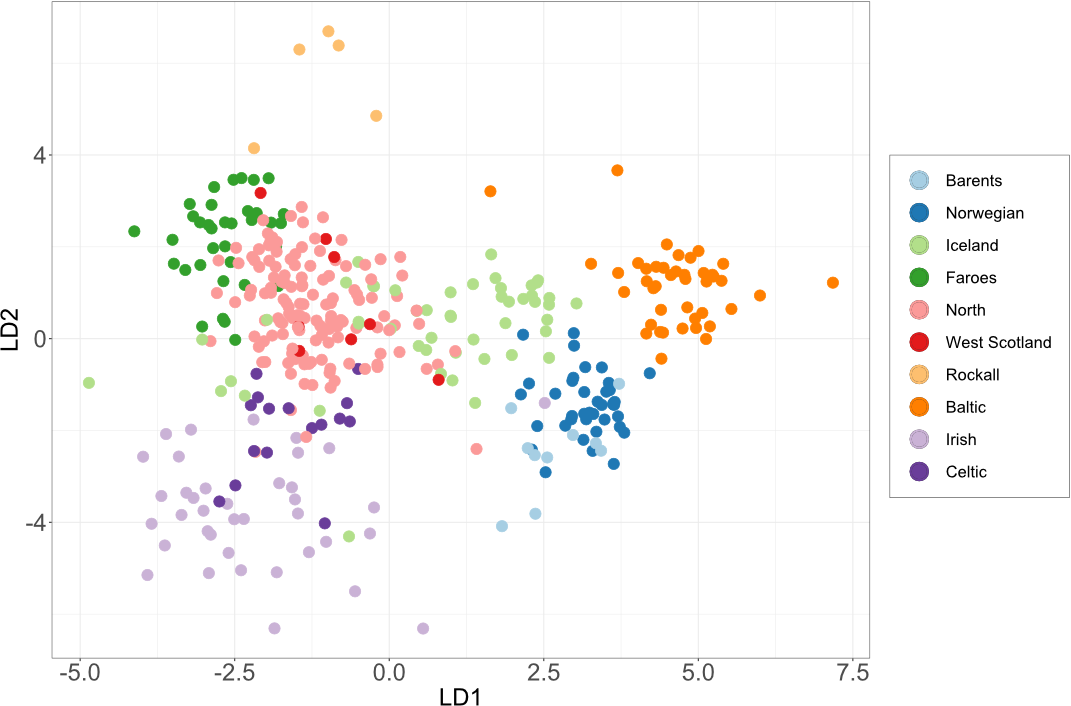


**Figure S5** Linear discriminant analysis (LD1 and LD2) using the carbon, nitrogen and sulfur stable isotope compositions measured in cod muscle tissue from each of the sampled regions.

The assignments to all regions using linear discriminant analysis and random forest classification are shown in more detail in Tables S6 and S7 respectively. Random forest classification gave the highest overall accuracy of assignment to origin (mean 79% for all regions), whereas discriminant analysis and the multivariate normal probability technique both gave similar, slightly lower accuracies – 70% and 72% mean overall respectively. Linear discriminant analysis performed poorly for West Scotland (0% correct) and the Celtic Sea (42% correct), but gave the highest assignment accuracy of all three techniques for the Norwegian and North Seas (99% and 88% correct). The multivariate technique was the most successful for the Iceland assignments, achieving at least 6% greater accuracy than the other two methods, whereas it gave a low assignment accuracy for the Rockall samples (40% correct compared to 100% using random forest). Random forest classification was the most successful method for the Celtic Sea, giving either 13% or 33% greater accuracy than the other techniques for these regions, whereas for the Barents Sea, Faroes and West Scotland samples, the multivariate technique and random forest performed equally well (90%, 86% and 50% respectively).

**Table S6** Assignment results to each of the geographic regions using linear discriminant analysis, showing the mean percentage over 1000 repeat simulations. The correct assignments for each region are shown in bold.

| Assigned origin | True origin region – percentage assigned (%) | | | | | | | | | |
| --- | --- | --- | --- | --- | --- | --- | --- | --- | --- | --- |
|  | Barents | Norwegian | Iceland | Faroes | North Sea | West Scotland | Rockall | Baltic | Irish | Celtic |
| Barents | **79** | 0 | 0 | 0 | 1 | 0 | 0 | 0 | 3 | 0 |
| Norwegian | 22 | **99** | 6 | 0 | 0 | 0 | 0 | 5 | 0 | 0 |
| Iceland | 0 | 0 | **61** | 0 | 5 | 13 | 0 | 2 | 0 | 0 |
| Faroes | 0 | 0 | 0 | **74** | 4 | 14 | 21 | 0 | 0 | 0 |
| North Sea | 0 | 0 | 25 | 26 | **88** | 74 | 0 | 0 | 0 | 33 |
| West Scotland | 0 | 0 | 0 | 0 | 0 | **0** | 0 | 0 | 0 | 0 |
| Rockall | 0 | 0 | 0 | 0 | 0 | 0 | **79** | 0 | 0 | 0 |
| Baltic | 0 | 0 | 2 | 0 | 0 | 0 | 0 | **93** | 0 | 0 |
| Irish | 0 | 0 | 2 | 0 | 1 | 0 | 0 | 0 | **84** | 25 |
| Celtic | 0 | 0 | 3 | 0 | 2 | 0 | 0 | 0 | 13 | **42** |

**Table S7** Leave-one-out cross validation results using random forest classification, showing the number of samples assigned to each of the geographic regions as well as the percentage of correct assignments for each region shown in bold.

| Assigned origin | True origin region – number assigned | | | | | | | | | |
| --- | --- | --- | --- | --- | --- | --- | --- | --- | --- | --- |
|  | Barents | Norwegian | Iceland | Faroes | North Sea | West Scotland | Rockall | Baltic | Irish | Celtic |
| Barents | **9** | 1 | 0 | 0 | 1 | 0 | 0 | 0 | 1 | 0 |
| Norwegian | 1 | **36** | 4 | 0 | 0 | 1 | 0 | 0 | 0 | 0 |
| Iceland | 0 | 2 | **32** | 0 | 10 | 0 | 0 | 1 | 0 | 0 |
| Faroes | 0 | 0 | 1 | **30** | 22 | 1 | 0 | 0 | 0 | 0 |
| North Sea | 0 | 0 | 3 | 2 | **71** | 2 | 0 | 0 | 0 | 0 |
| West Scotland | 0 | 0 | 3 | 3 | 25 | **4** | 0 | 0 | 0 | 1 |
| Rockall | 0 | 0 | 0 | 0 | 0 | 0 | **5** | 1 | 0 | 0 |
| Baltic | 0 | 1 | 0 | 0 | 0 | 0 | 0 | **40** | 0 | 0 |
| Irish | 0 | 0 | 1 | 0 | 0 | 0 | 0 | 0 | **32** | 3 |
| Celtic | 0 | 0 | 6 | 0 | 8 | 0 | 0 | 0 | 5 | **12** |
| Percentage correct | **90%** | **90%** | **64%** | **86%** | **52%** | **50%** | **100%** | **95%** | **84%** | **75%** |

Figure S6 shows the assignment accuracy of cod to the ICES subareas within FAO region 27 using random forest classification, to demonstrate the potential to distinguish among these regions in a fisheries management context.


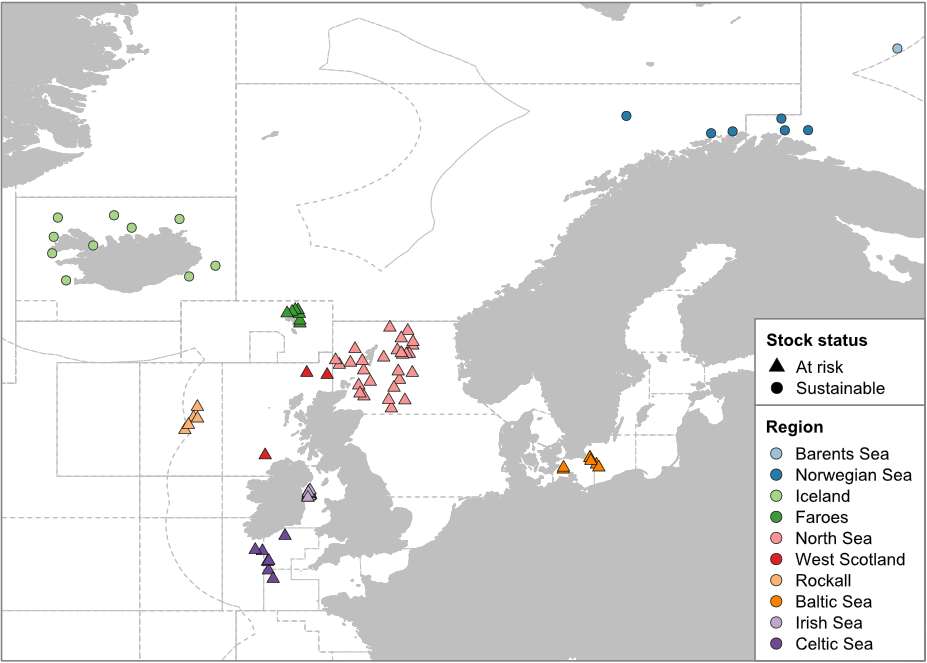


**Figure S6** Locations of sampling stations within ICES sub area boundaries (grey lines), and assignment success rates in each region using random forest classification with a leave-one-out cross validation approach.

64%

84%

75%

50%

100%

86%

52%

90%

90%

95%

###

### Assigning independent known origin samples to the reference dataset

Isotope data from fully independently collected test samples (Jennings and Cogan^54^, Institute of Marine Research (IMR, Norway), Young’s Seafood Ltd. and Ifremer^55^ are shown in Figure S7. This demonstrates that the independent samples overlap with our reference data, although no independent data were obtained for the Baltic Sea.


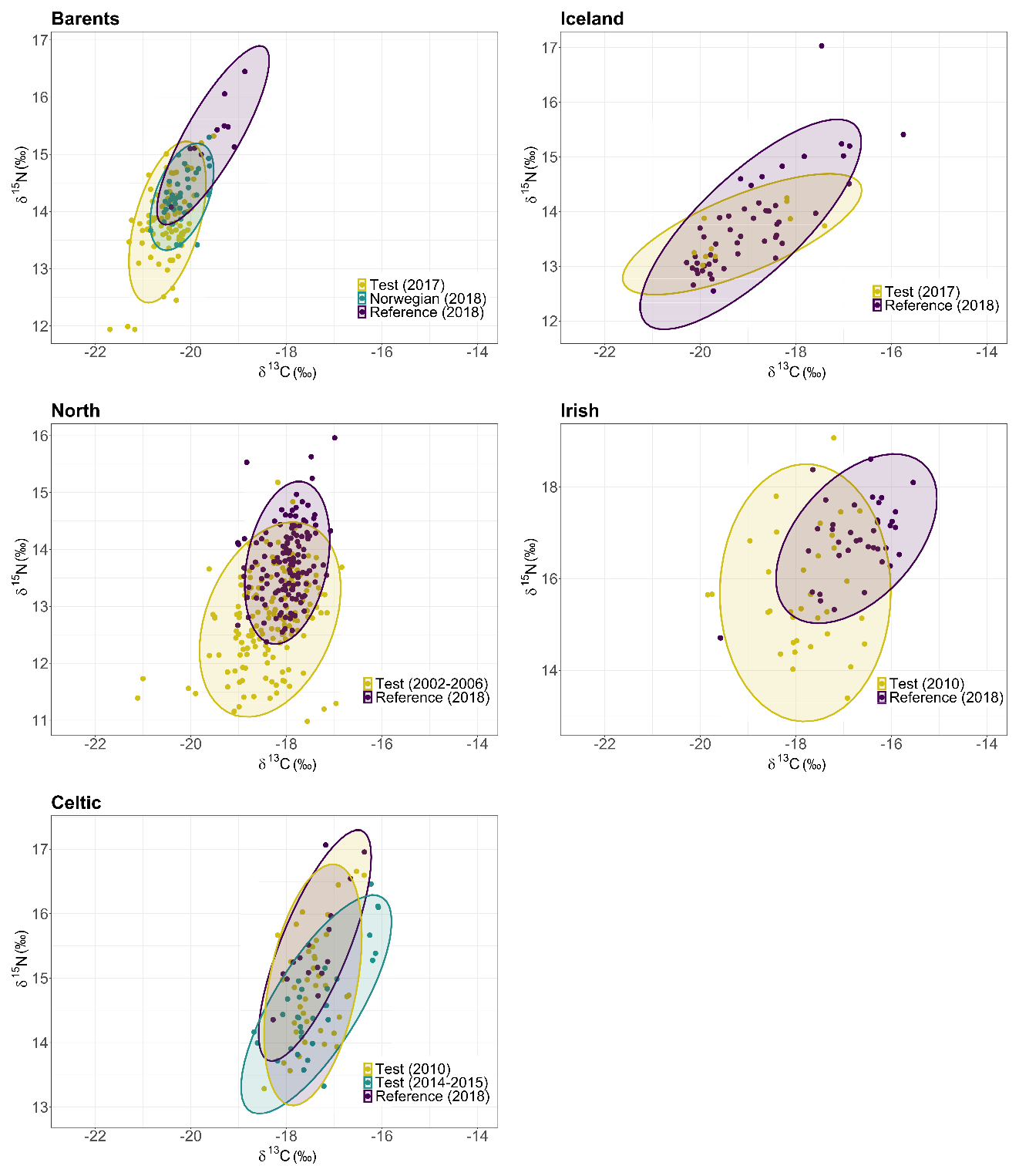


**Figure S7** Carbon and nitrogen stable isotope values measured in cod samples collected in this study (reference samples) compared with those collected from previous years and studies (test samples), after applying Suess correction on δ^13^C values. Previous data: Barents 2017 – collected by Institute of Marine Research (Norway); Iceland 2017 – provided by Young’s Seafood Ltd.; North Sea 2002-2006 – from Jennings and Cogan^54^; Irish Sea 2010 – from Jennings and Cogan^54^; Celtic Sea 2010 – from Jennings and Cogan^54^, Celtic Sea 2014-2015 – collected by Ifremer^55^ from the EVHOE 2014 survey.

**DISCUSSION**

**Assignment of independent known origin samples**

The locations of the independently collected samples from Jennings and Cogan^54^ relative to the stations sampled in the current study are displayed in Figure S8. The samples in Jennings and Cogan^54^ from the North and Celtic seas were collected from similar locations to our reference samples, but those from the Irish Sea cover a much wider area.


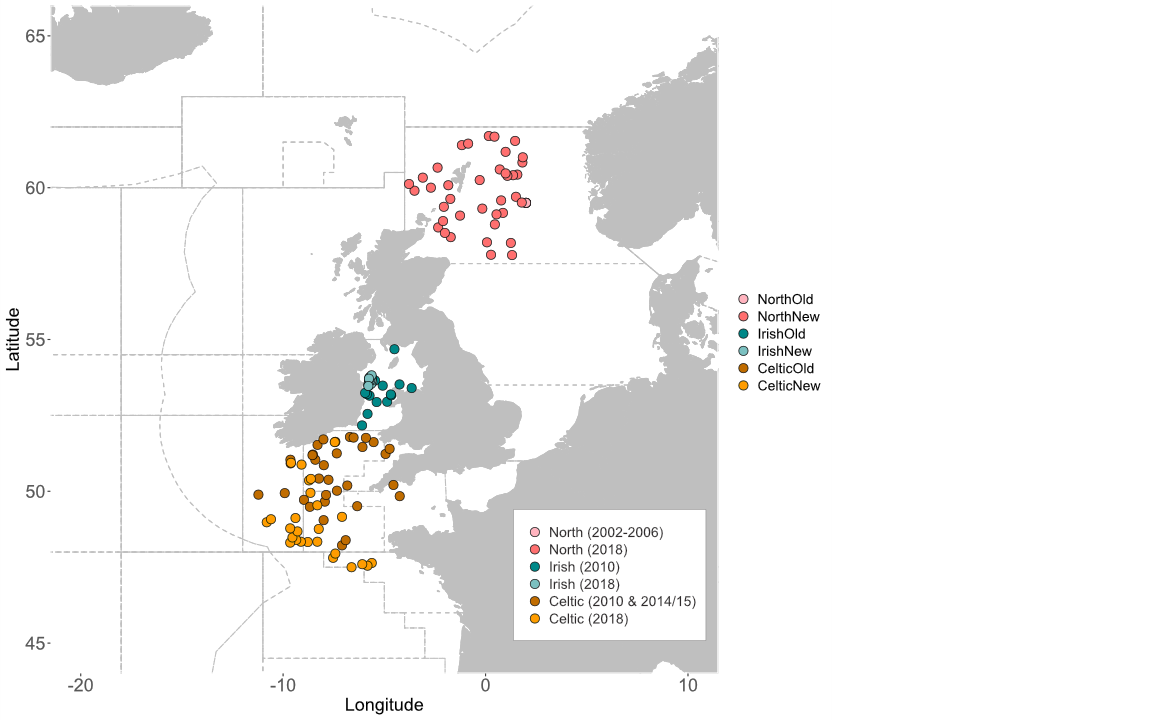


**Figure S8** Locations where independent cod samples were collected previously by Jennings and Cogan^54^ compared to the locations where samples were collected for the current study in 2018 in the corresponding regions.
