# Supplementary material for: Tracing the geographic origin of Atlantic cod products using stable isotope analysis
Source: Rapid Commun Mass Spectrom. 2024 Jul 22;39(Suppl 1):e9861. doi: 10.1002/rcm.9861 (PMC12062778; doi:10.1002/rcm.9861)
Supplement: Supplementary file 12 — Table S1 Total number of individual Atlantic cod collected from each geographic region, the number of stations sampled per region, and the source from which samples were obtained. [file RCM-39-e9861-s003.docx]

**Table S1** Total number of individual Atlantic cod collected from each geographic region, the number of stations sampled per region, and the source from which samples were obtained.

| Catch region | Number of cod sampled | Number of stations | Year samples collected | Source of samples and organisation name |
| --- | --- | --- | --- | --- |
| Barents Sea | 10 | Unknown | 2017 | Young’s Seafood Ltd. |
| Norwegian Sea | 40 | 6 | 2018 | Annual fisheries survey - Institute of Marine Research |
| Iceland | 50 | 10 | 2018 | Annual fisheries survey - Marine and Freshwater Research Institute |
| Faroe Islands | 35 | 7 | 2018 | Annual fisheries survey - Faroe Marine Research Institute |
| North Sea | 133 | 28 | 2018 | Annual fisheries survey - Marine Scotland |
| West Scotland | 8 | 3 | 2018 | Annual fisheries survey - Marine Scotland |
| Rockall | 5 | 5 | 2018 | Annual fisheries survey - Marine Scotland |
| Baltic Sea | 42 | 7 | 2018 | Fish tagging survey - Technical University of Denmark |
| Irish Sea | 38 | 16 | 2018 | Fish tagging survey - Marine Institute |
| Celtic Sea | 16 | 7 | 2018 | Annual fisheries survey - Ifremer |
| Total | **377** | **89** |  |  |
