# Supplementary material for: Tracing the geographic origin of Atlantic cod products using stable isotope analysis
Source: Rapid Commun Mass Spectrom. 2024 Jul 22;39(Suppl 1):e9861. doi: 10.1002/rcm.9861 (PMC12062778; doi:10.1002/rcm.9861)
Supplement: Supplementary file 13 — Table S2 Comparison of the mean values and uncertainties (standard deviations) in the stable isotope measurements of two internal standards measured at both laboratories where samples were analysed. [file RCM-39-e9861-s016.docx]

**Table S2** Comparison of the mean values and uncertainties (standard deviations) in the stable isotope measurements of two internal standards measured at both laboratories where samples were analysed.

| Standard | Laboratory | δ^13^C (‰) | | δ^15^N (‰) | | δ^34^S (‰) | |
| --- | --- | --- | --- | --- | --- | --- | --- |
|  |  | **Mean** | **SD** | **Mean** | **SD** | **Mean** | **SD** |
| Fish muscle | NOCS | -19.3 | 0.11 | 11.4 | 0.17 | 19.3 | 0.64 |
|  | LSMSF | -19.3 | 0.07 | 11.3 | 0.11 | 18.5 | 0.74 |
| Glutamic acid | NOCS | -13.1 | 0.17 | -3.8 | 0.06 | NA | NA |
|  | LSMSF | -13.6 | 0.43 | -3.9 | 0.15 | NA | NA |
