# Supplementary material for: Tracing the geographic origin of Atlantic cod products using stable isotope analysis
Source: Rapid Commun Mass Spectrom. 2024 Jul 22;39(Suppl 1):e9861. doi: 10.1002/rcm.9861 (PMC12062778; doi:10.1002/rcm.9861)
Supplement: Supplementary file 14 — Table S3 Means and standard deviations of carbon (lipid corrected), nitrogen and sulfur stable isotope ratios from Atlantic cod caught in each of the sampled geographic regions. [file RCM-39-e9861-s008.docx]

**Table S3** Means and standard deviations of carbon (lipid corrected), nitrogen and sulfur stable isotope ratios from Atlantic cod caught in each of the sampled geographic regions.

| Region | Number of samples | δ^13^C (‰) | | δ^15^N (‰) | | δ^34^S (‰) | |
| --- | --- | --- | --- | --- | --- | --- | --- |
|  |  | **Mean** | **SD** | **Mean** | **SD** | **Mean** | **SD** |
| Barents Sea | 10 | -19.5 | 0.48 | 15.3 | 0.64 | 18.2 | 0.64 |
| Norwegian Sea | 40 | -20.2 | 0.30 | 14.3 | 0.42 | 19.7 | 0.50 |
| Iceland | 50 | -19.0 | 1.05 | 13.8 | 0.87 | 18.7 | 0.92 |
| Faroe Islands | 35 | -17.4 | 0.37 | 13.1 | 0.64 | 18.5 | 0.41 |
| North Sea | 133 | -18.0 | 0.42 | 13.8 | 0.66 | 19.0 | 0.50 |
| West Scotland | 8 | -18.3 | 0.41 | 13.5 | 0.73 | 19.4 | 0.55 |
| Rockall | 5 | -18.8 | 0.43 | 10.5 | 0.63 | 18.7 | 0.63 |
| Baltic Sea | 42 | -21.1 | 0.36 | 12.7 | 0.49 | 18.1 | 1.10 |
| Irish Sea | 38 | -16.7 | 0.76 | 16.9 | 0.83 | 18.2 | 0.83 |
| Celtic Sea | 16 | -17.4 | 0.51 | 15.5 | 0.77 | 18.8 | 0.70 |
