# Supplementary material for: Tracing the geographic origin of Atlantic cod products using stable isotope analysis
Source: Rapid Commun Mass Spectrom. 2024 Jul 22;39(Suppl 1):e9861. doi: 10.1002/rcm.9861 (PMC12062778; doi:10.1002/rcm.9861)
Supplement: Supplementary file 15 — Table S4 Mean percentage of individuals assigned to the correct origin region over 1000 repeat simulations using three isotopes (δ13C, δ15N and δ34S) and two isotopes (δ13C and δ15N). [file RCM-39-e9861-s001.docx]

**Table S4** Mean percentage of individuals assigned to the correct origin region over
1000 repeat simulations using three isotopes (δ^13^C, δ^15^N and δ^34^S) and two isotopes
(δ^13^C and δ^15^N).

| Region | Mean correct assignments (%) | |
| --- | --- | --- |
|  | **CNS** | **CN** |
| Barents | 83 | 80 |
| Norwegian | 95 | 82 |
| Iceland | 73 | 59 |
| Faroes | 85 | 87 |
| North Sea | 52 | 50 |
| West Scotland | 44 | 40 |
| Rockall | 40 | 61 |
| Baltic | 94 | 94 |
| Irish | 80 | 84 |
| Celtic | 62 | 70 |
| Total | **71%** | **71%** |
