# Supplementary material for: Tracing the geographic origin of Atlantic cod products using stable isotope analysis
Source: Rapid Commun Mass Spectrom. 2024 Jul 22;39(Suppl 1):e9861. doi: 10.1002/rcm.9861 (PMC12062778; doi:10.1002/rcm.9861)
Supplement: Supplementary file 16 — Table S5 Leave‐one‐out cross validation results using the multivariate normal probability method, showing the number of samples assigned to each of the geographic regions as well as the percentage of correct assignments for each region. True known origins are shown in the columns and the assigned most likely origins are shown in the rows. [file RCM-39-e9861-s013.docx]

**Table S5** Leave-one-out cross validation results using the multivariate normal probability method, showing the number of samples assigned to each of the geographic regions as well as the percentage of correct assignments for each region. True known origins are shown in the columns and the assigned most likely origins are shown in the rows.

| Assigned origin | True origin region – number assigned | | | | | | | | | |
| --- | --- | --- | --- | --- | --- | --- | --- | --- | --- | --- |
|  | Barents | Norwegian | Iceland | Faroes | North Sea | West Scotland | Rockall | Baltic | Irish | Celtic |
| Barents | **9** | 0 | 1 | 0 | 0 | 0 | 0 | 0 | 1 | 0 |
| Norwegian | 1 | **39** | 1 | 0 | 0 | 0 | 0 | 0 | 0 | 0 |
| Iceland | 0 | 1 | **35** | 1 | 15 | 1 | 0 | 1 | 0 | 1 |
| Faroes | 0 | 0 | 1 | **30** | 14 | 1 | 3 | 0 | 0 | 0 |
| North Sea | 0 | 0 | 4 | 2 | **64** | 2 | 0 | 0 | 0 | 0 |
| West Scotland | 0 | 0 | 2 | 2 | 33 | **4** | 0 | 0 | 0 | 1 |
| Rockall | 0 | 0 | 0 | 0 | 0 | 0 | **2** | 0 | 0 | 0 |
| Baltic | 0 | 0 | 0 | 0 | 0 | 0 | 0 | **41** | 0 | 0 |
| Irish | 0 | 0 | 2 | 0 | 1 | 0 | 0 | 0 | **31** | 4 |
| Celtic | 0 | 0 | 4 | 0 | 10 | 0 | 0 | 0 | 6 | **10** |
| Percentage correct | **90%** | **98%** | **70%** | **86%** | **47%** | **50%** | **40%** | **98%** | **82%** | **63%** |
