# Supplementary material for: Tracing the geographic origin of Atlantic cod products using stable isotope analysis
Source: Rapid Commun Mass Spectrom. 2024 Jul 22;39(Suppl 1):e9861. doi: 10.1002/rcm.9861 (PMC12062778; doi:10.1002/rcm.9861)
Supplement: Supplementary file 17 — Table S6 Assignment results to each of the geographic regions using linear discriminant analysis, showing the mean percentage over 1000 repeat simulations. The correct assignments for each region are shown in bold. [file RCM-39-e9861-s014.docx]

**Table S6** Assignment results to each of the geographic regions using linear discriminant analysis, showing the mean percentage over 1000 repeat simulations. The correct assignments for each region are shown in bold.

| Assigned origin | True origin region – percentage assigned (%) | | | | | | | | | |
| --- | --- | --- | --- | --- | --- | --- | --- | --- | --- | --- |
|  | Barents | Norwegian | Iceland | Faroes | North Sea | West Scotland | Rockall | Baltic | Irish | Celtic |
| Barents | **79** | 0 | 0 | 0 | 1 | 0 | 0 | 0 | 3 | 0 |
| Norwegian | 22 | **99** | 6 | 0 | 0 | 0 | 0 | 5 | 0 | 0 |
| Iceland | 0 | 1 | **61** | 0 | 5 | 13 | 0 | 2 | 0 | 0 |
| Faroes | 0 | 0 | 0 | **74** | 4 | 14 | 21 | 0 | 0 | 0 |
| North Sea | 0 | 0 | 25 | 26 | **88** | 74 | 0 | 0 | 0 | 33 |
| West Scotland | 0 | 0 | 0 | 0 | 0 | **0** | 0 | 0 | 0 | 0 |
| Rockall | 0 | 0 | 0 | 0 | 0 | 0 | **79** | 0 | 0 | 0 |
| Baltic | 0 | 0 | 2 | 0 | 0 | 0 | 0 | **93** | 0 | 0 |
| Irish | 0 | 0 | 2 | 0 | 1 | 0 | 0 | 0 | **84** | 25 |
| Celtic | 0 | 0 | 3 | 0 | 2 | 0 | 0 | 0 | 13 | **42** |
