# Supplementary material for: Tracing the geographic origin of Atlantic cod products using stable isotope analysis
Source: Rapid Commun Mass Spectrom. 2024 Jul 22;39(Suppl 1):e9861. doi: 10.1002/rcm.9861 (PMC12062778; doi:10.1002/rcm.9861)
Supplement: Supplementary file 18 — Table S7 Leave‐one‐out cross validation results using random forest classification, showing the number of samples assigned to each of the geographic regions as well as the percentage of correct assignments for each region shown in bold. [file RCM-39-e9861-s006.docx]

**Table S7** Leave-one-out cross validation results using random forest classification, showing the number of samples assigned to each of the geographic regions as well as the percentage of correct assignments for each region shown in bold.

| Assigned origin | True origin region – number assigned | | | | | | | | | |
| --- | --- | --- | --- | --- | --- | --- | --- | --- | --- | --- |
|  | Barents | Norwegian | Iceland | Faroes | North Sea | West Scotland | Rockall | Baltic | Irish | Celtic |
| Barents | **9** | 1 | 0 | 0 | 1 | 0 | 0 | 0 | 1 | 0 |
| Norwegian | 1 | **36** | 4 | 0 | 0 | 1 | 0 | 0 | 0 | 0 |
| Iceland | 0 | 2 | **32** | 0 | 10 | 0 | 0 | 1 | 0 | 0 |
| Faroes | 0 | 0 | 1 | **30** | 22 | 1 | 0 | 0 | 0 | 0 |
| North Sea | 0 | 0 | 3 | 2 | **71** | 2 | 0 | 0 | 0 | 0 |
| West Scotland | 0 | 0 | 3 | 3 | 25 | **4** | 0 | 0 | 0 | 1 |
| Rockall | 0 | 0 | 0 | 0 | 0 | 0 | **5** | 1 | 0 | 0 |
| Baltic | 0 | 1 | 0 | 0 | 0 | 0 | 0 | **40** | 0 | 0 |
| Irish | 0 | 0 | 1 | 0 | 0 | 0 | 0 | 0 | **32** | 3 |
| Celtic | 0 | 0 | 6 | 0 | 8 | 0 | 0 | 0 | 5 | **12** |
| Percentage correct | **90%** | **90%** | **64%** | **86%** | **52%** | **50%** | **100%** | **95%** | **84%** | **75%** |
